# TRIAGE: Trustworthy Reporting and Assessment for Clinical Gain and Effectiveness of AI Models

**DOI:** 10.3390/diagnostics16050666

**Published:** 2026-02-25

**Authors:** Farzaneh Fazilati, Mohammad Zakaria Rajabi, Nima Alihosseini, Mohaddeseh Esmaeili Farsani, Seyed Hasan Sandid, Shadi Zamani, Mehrshad Alirezaei Farahani, Fateme Biriaei, Fateme Sadeghipour, Mohammad Taha Mirshamsi, Mottahareh Fahami, Hamid Reza Marateb

**Affiliations:** 1 Department of Biomedical Engineering, Faculty of Engineering, University of Isfahan, Isfahan 8174673441, Iran; farzanehfazilati@gmail.com (F.F.); nimaalihosseini@eng.ui.ac.ir (N.A.); mohaddesehesmaeilifarsani@eng.ui.ac.ir (M.E.F.); hasan.shs110@gmail.com (S.H.S.); shadizamani710@gmail.com (S.Z.); m.alirezaei@eng.ui.ac.ir (M.A.F.); fa671biriyaei@gmail.com (F.B.); sadeghipourfateme1921@gmail.com (F.S.); mt.mirshamsi@eng.ui.ac.ir (M.T.M.); m.fahami.81@gmail.com (M.F.); 2 Department of Biomedical Engineering, School of Medicine, Tehran University of Medical Science, Tehran 88989487, Iran; mz-rajabib@razi.tums.ac.ir; 3 Institute for Research and Innovation in Health (IRIS), Universitat Politècnica de Catalunya—BarcelonaTech (UPC), 08028 Barcelona, Spain; 4 Center for Innovation and Development of Artificial Intelligence Technologies, University of Isfahan, Isfahan 81746773441, Iran

**Keywords:** machine learning evaluation, performance metrics, robustness, cross-validation, statistical significance testing, representation curves

## Abstract

Machine learning (ML), including deep learning, kernel-based classifiers, and ensemble methods, is increasingly used to support clinical diagnosis in medical imaging, biosignal interpretation, and electronic health record (EHR)-based decision support. Despite rapid progress, many diagnostic AI studies still rely on limited retrospective evaluation and single summary measures (e.g., accuracy or AUC), creating a gap between reported model performance and evidence required for safe clinical adoption. This review proposes TRIAGE, a clinically grounded evaluation framework designed to organize diagnostic AI testing as an evidence pipeline aligned with real clinical use cases (screening, triage, second reading, and confirmatory testing). We summarize core discrimination metrics derived from the confusion matrix (sensitivity, specificity, predictive values, likelihood ratios, diagnostic odds ratio, and F-scores) and highlight the importance of prevalence and spectrum effects for interpreting predictive value and clinical workload. We further review evaluation strategies for multi-class and multi-label diagnostic tasks using appropriate aggregation methods (micro, macro, and weighted averaging) and set-based measures such as Hamming loss, exact match ratio, and Jaccard/IoU. Because diagnostic deployment is threshold-dependent, we integrate representation curves (ROC, precision–recall, lift, and cumulative gain) with calibration assessment and clinical utility analysis, including calibration slope, Brier score, and decision-curve analysis. We also address robustness and fairness evaluation, leakage-resistant validation designs (patient-grouped splits, stratified and temporal validation, and external validation), computational constraints relevant to deployment (latency, throughput, and energy use), and statistically sound model comparison with multiplicity control. A structured TRIAGE checklist table summarizing the evaluation parameters described in this review is provided in the main text to support reproducible and clinically interpretable reporting.

## 1. Introduction

Over the past decade and particularly in recent years, artificial intelligence (AI) and machine learning (ML) have become increasingly integrated into clinical decision-making, including diagnostic imaging, biosignal interpretation, pathology, and electronic health record analysis. As these systems begin to influence high-stakes medical decisions, the need for transparent and clinically meaningful evaluation has become more important than ever. In diagnostic medicine, model performance cannot be adequately summarized by a single metric, especially when clinical consequences depend on prevalence, spectrum effects, and the relative harm of false-positive versus false-negative outcomes [1,2].

Evaluating classification models, whether binary, multi-class, or multi-label, is therefore a central component of diagnostic AI development and reporting. Commonly used measures include confusion-matrix derived indices (e.g., sensitivity, specificity, predictive values, likelihood ratios, and F-scores), as well as discrimination curves such as receiver operating characteristic (ROC) and precision-recall (PR) curves [3,4]. However, these measures provide different perspectives and may lead to different interpretations depending on the clinical context. For example, ROC-based evaluation can appear optimistic under severe class imbalance, whereas PR-based evaluation is often more informative for rare conditions [5]. In multi-label diagnostic tasks, metrics such as Hamming loss, exact match ratio, and set-based similarity measures (e.g., Jaccard index/IoU) can better reflect real-world diagnostic complexity where multiple findings may coexist. For this reason, relying on a single summary statistic is often insufficient and may be misleading, particularly in heterogeneous or imbalanced clinical datasets [6,7].

Beyond overall accuracy, evaluation of diagnostic AI must also address fairness, bias, and robustness to ensure safe clinical use [8]. In high-stakes healthcare settings, models should perform consistently across clinically relevant subgroups and remain stable under realistic sources of measurement variability [9]. Fairness assessment therefore examines whether error rates, such as false positives and false negatives, differ systematically between demographic groups, with operational criteria such as equalized odds providing a practical framework for identifying disparities [10]. Complementary robustness analyses assess the sensitivity of model performance to small, plausible perturbations of input features, reflecting common sources of noise in clinical data [11]. Together, these evaluations help ensure that diagnostic AI systems are not only accurate, but also reliable, equitable, and suitable for real-world deployment [12].

Validation design is equally critical. Cross-validation methods such as k-fold, stratified, grouped, and temporal validation can reduce optimistic bias and improve the credibility of reported results, particularly when datasets are limited or correlated at the patient level [13,14]. In addition, reporting calibration performance (e.g., calibration slope, Brier score) and clinical utility measures (e.g., decision-curve analysis and net benefit) is recommended when model outputs are intended to support risk estimation or diagnostic decision-making [15]. These tools help bridge the translation gap between statistically impressive performance and real-world clinical value.

Insufficient evaluation of diagnostic AI systems can have direct consequences for patient care. False negatives may delay treatment, while false positives can trigger unnecessary testing, anxiety, or invasive interventions. Moreover, models may appear reliable in retrospective internal validation yet fail when deployed across sites, devices, or patient populations [16]. For this reason, diagnostic AI evaluation must go beyond technical model accuracy and instead emphasize clinically interpretable reporting, appropriate validation strategies, and evidence aligned with intended clinical use.

**Figure 1:**
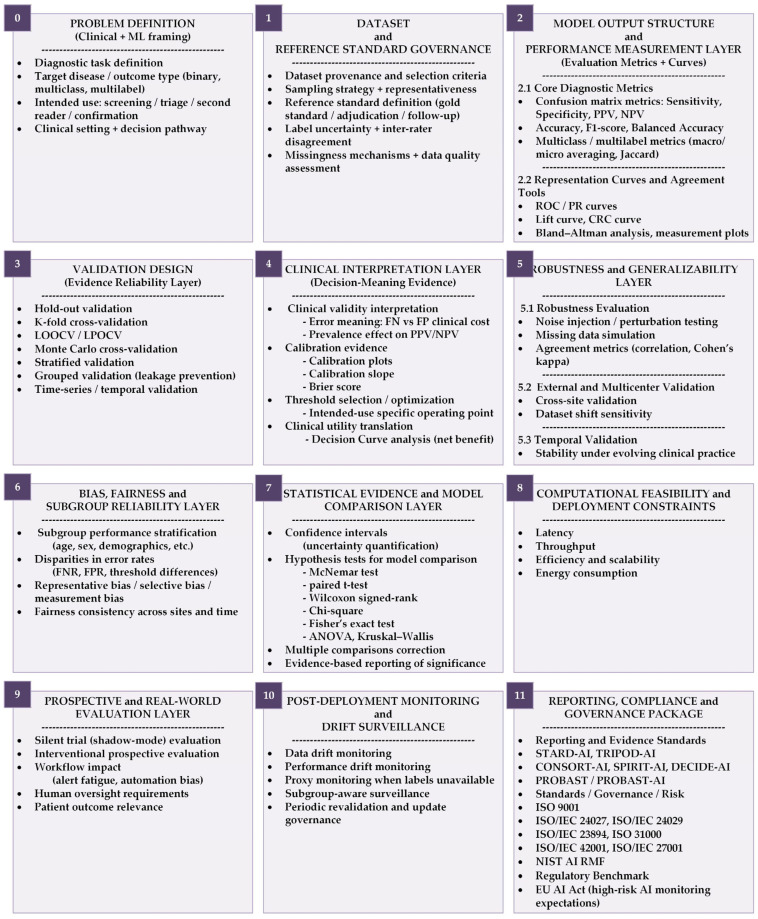
General block diagram of the evaluation and testing items described in this review.

This article is a narrative, guidance-oriented review and framework paper rather than a systematic review. Its aim is not to provide exhaustive coverage of all existing evaluation metrics, but to offer a clinically grounded structure for selecting, interpreting, and reporting evaluation measures in diagnostic AI. The evaluation components discussed were selected based on their frequent use in diagnostic AI research, their relevance to clinical decision-making, and their alignment with major reporting recommendations and standardization efforts. Particular emphasis is placed on linking statistical and evaluative performance measures to intended diagnostic use cases and to practical considerations relevant to real-world deployment. The proposed TRIAGE framework integrates these perspectives, is summarized in Figure 1, and is described in detail throughout the paper. To enhance practical usability, a structured TRIAGE checklist summarizing each evaluation metric (definition, strengths, limitations, appropriate and inappropriate use) is provided in Supplementary File S1. For transparency and governance alignment, the correspondence between the TRIAGE evaluation components and relevant clauses of international standards and guidelines is summarized in Table S1.

## 2. Evaluation Metrics

A dataset can be defined by some of its defining features. Total population refers to the total count of samples in the dataset. Population is further described by its real-world condition, where condition positive refers to the count of actual positive cases and condition negative describes the total number of actual negative instances. In terms of classification outcomes, prediction positive indicates the number of samples classified as positive, while prediction negative refers to those classified as negative [17]. Additionally, prevalence is a critical measure, defined as the proportion of a specific class in relation to the entire number of samples [18].

### 2.1. Binary and Multi-Class Classification Models

According to the classification system in Table 1, every sample in a dataset is evaluated by comparing its predicted status against its ground truth label. This evaluation can result in four possible outcomes. The two categories of correct classification are true positive (hit), where an instance is accurately identified as positive based on the fact that it belongs to the class, and true negative (correct rejection), where a sample is accurately regarded as negative based on the fact that the sample is not a member of the considered class. Conversely, there are two types of misclassification. A false positive (false alarm, type I error) occurs when an observation that is not a member of the class is incorrectly classified as one. The second type of error is a false negative (miss, type II error), which describes a sample that is a member of the class but is incorrectly predicted not to be [17].

Several key metrics are used to evaluate a model’s performance on a per-class basis. The true positive rate (TPR), also referred to as sensitivity or recall, is the proportion of actual positive objects that are accurately identified as positive. Its counterpart is the true negative rate (TNR), also known as specificity or selectivity, which is defined as the ratio of correctly classified negative cases among all actual negative cases [3]. Correspondingly, the error rates provide insight into misclassifications. The false positive rate (FPR), also called fall-out or the probability of a false alarm, represents the proportion of negative instances that are incorrectly identified as positive. False negative rate (FNR), commonly called the miss rate, describes the proportion of positive instances that have been incorrectly flagged as negative [19].

While fundamental rates evaluate effectiveness in relation to the ground truth, predictive values focus on how reliable the predictions are. The positive predictive value (PPV), also known as precision or relevance, measures the proportion of true positive results in relation to all outcomes that were predicted positive. The same logic applies to negative predictive value (NPV), which is also known as separation ability. NPV describes the true negative results in relation to all outcomes that were predicted to be negative [18]. To present some summary insight into a model’s performance over all classes, accuracy is calculated, which is defined as the ratio of all correct classifications to the total number of instances in the dataset [17,20].

Additional metrics describe the specific rate of error of given predictions. The false discovery rate (FDR) is the measure describing the proportion of incorrect rejects (false positives or type I errors) from the total accepted nulls. In parallel, the false omission rate (FOR) is the proportion of false negative results among all instances that received a negative prediction [19].

Finally, several metrics combine the above rates to offer more nuanced diagnostic insights. The positive likelihood ratio is calculated by dividing the true positive rate by the false positive rate, while the negative likelihood ratio is the ratio of the false negative rate to the true negative rate [18]. The DOR or diagnostic odds ratio gives one summary data point indicating the odds of correctly recognizing a case and the odds of wrongly identifying it, regardless of how common it is [3].

In class evaluation, binary classification problems are of particular interest, especially when working with prediction problems where the data are highly skewed or when the cost of false negatives is different from the cost of false positives. The F_β_ score is the most common score for such cases. The F_β_ score is defined in its most basic form as the weighted harmonic mean of precision and recall, and the distance from one, which is on a scale of 0 and 2, is the F_β_ value. Recall is the number of positive examples returned from all returned examples, and recall refers to all examples that were correctly predicted to be positive. The parameter beta is added to increase the importance of recall compared to precision [21].

When the beta value is equal to unity, the F_β_ score is usually known as a score of 1, and it is commonly said that it considers both precision and recall equally. It is most helpful in understanding the performance of a classifier if both false positives and false negatives are considered equally important. Also, the relative differences between F_1_ and the above criteria, which are determined as beta, F_1_ is equal to 1, where there is the largest absolute difference, and covers the most important areas where missing a positive result is necessary, such as medical cases. Equation (1) describes the $F_{\beta }$ score [21]. All the parameters described are summarized in Table 2.

(1) $$F_{\beta }=\left(1+\beta^{2}\right)\times \frac{Precision\times Recall}{\left(\beta^{2}\times Precision\right)+Recall}$$

In a cohort of 1000 individuals, a screening test for a cardiac disorder was conducted, and 200 of them were already confirmed cases. Each case was shown to have 160 true positives, 40 false negatives, 100 false positives, and 700 true negatives.

The sensitivity of 0.80 means that the test was able to detect cases, and the specificity of 0.875 was able to correctly determine the unaffected individuals. Positive predictive value is low, at 0.615, which means that only under 6 for every 10 test positives actually have the disease, which shows how positive results can be very unreliable in a low-prevalence setting. Negative predictive value is highly reliable for a negative result and is shown with 0.946. False negative rate demonstrates the proportion of missed cases at 0.20, while the proportion of unnecessary follow-up to healthy individuals is shown with the false positive rate at 0.125. The positive likelihood ratio of 6.4, and negative of 0.229, together with the diagnostic odds ratio of 28, provide an overall proxy measure with noted shifts in either direction around the odds. This specific example shows that the metrics of the test are more reliable in assisting prediction by either proving or disproving the disease in the subject.

#### 2.1.1. Averaging

When assessing neural network models and classification models, it is important to consider the performance metrics at the multi-class level. For example, in an imbalanced dataset where some classes have considerably more instances than others, the method of metric combination chosen may greatly alter the outcome. In this context, micro-averaging and macro-averaging are two prominent aggregation strategies.

Micro-averaged metrics combine the counts from each class by summing together the true positives (counting how many positives were predicted correctly (*TPs*)), false positives (predicting positives incorrectly (*FPs*)), and false negatives (counting the negatives that were classified incorrectly (*FNs*)) and then defining performance measures based on those aggregated counts. In this case, all instances are treated without class distinction. Micro-averaged precision is then calculated in terms of that summation, as stated in Equation (2). Micro-averaged recall is treated the same as stated in Equation (3). Subsequently, the micro-averaged F_1_ score is calculated from the micro-averaged precision and recall values as stated in Equation (4) [22,23,24].

(2) $${Precision}_{micro}=\frac{\sum_{i=1}^{K}TP_{i}}{\sum_{i=1}^{K}\left(TP_{i}+FP_{i}\right)}$$

(3) $${Recall}_{micro}=\frac{\sum_{i=1}^{K}TP_{i}}{\sum_{i=1}^{K}\left(TP_{i}+FN_{i}\right)}$$

(4) $${F_{\beta }}_{micro}=\frac{\left(1+\beta^{2}\right)\times {Precision}_{micro}\times {Recall}_{micro}}{{Precision}_{micro}+{Recall}_{micro}}$$

Here, *K* represents the complete count of classes, and this addition is performed over all classes. Although micro-averaging is a useful indicator of a system’s overall performance, it can obscure the model’s behavior in less frequent classes. This becomes especially problematic with imbalanced datasets because the metric can become biased by being dominated by the performance on the majority group [22,23,24].

On the other hand, macro-averaging looks at the metric for each class separately and then averages the class-specific metrics. This means that each class will have equal importance assigned to it, irrespective of its occurrence in the data sample. For the macro-averaged precision, one can use Equation (5). For the macro-averaged recall, one can use Equation (6). Then, one can obtain the macro-averaged F1 score from the defined precision and recall as shown in Equation (7) [22,23,24].

(5) $${Precision}_{macro}=\frac{1}{K}\sum_{i=1}^{K}\frac{{TP}_{i}}{{TP}_{i}+{FP}_{i}}$$

(6) $${Recall}_{macro}=\frac{1}{K}\sum_{i=1}^{K}\frac{{TP}_{i}}{{TP}_{i}+{FN}_{i}}$$

(7) $${F_{\beta }}_{macro}=\frac{1}{K}\sum_{i=1}^{K}\frac{\left(1+\beta^{2}\right)\times {Precision}_{i}\times {Recall}_{i}}{{Precision}_{i}+{Recall}_{i}}$$

By averaging metrics on a per-class basis, macro-averaging highlights factors affecting minority classes, which micro-averaging may ignore. As a result, macro metrics have particular relevance in assessing the robustness of the classifier in every region of its decision space [22,23,24].

In the context of robustness analysis, macro average metrics will take precedence when the aim is to enforce a consistent performance function across all classes, including those classes that have fewer examples. However, in applications where poor performance on any single class is unacceptable, a model cannot be considered robust if it performs poorly on rare or difficult classes, even if its overall average performance is high [22,23,24].

Essentially, micro-averaging reflects overall accuracy on a per-sample basis, favoring prevalent classes, whereas macro-averaging reflects performance on a per-class basis, treating all classes equally. Hence, the ideal choice should be dictated by the robustness requirements considered in the particular application domain [22,23,24].

An algorithm used for diagnosis delineated three subtypes of cardiac arrhythmia for which it performed class-specific predictions. With comparison of reference labels for these predictions, the micro-averaged attributed metrics were a precision of 0.84, a recall of 0.77, and an F_1_ score of 0.80, which indicated results for every single patient. The results, on the other hand, proved the macro-averaged results to be lower, with a precision of 0.76, a recall of 0.69, and an F_1_ score of 0.72, indicating marked underperformance on these results by subminority type subgroups.

These results highlight the impact of class imbalance on aggregated evaluation metrics. Micro-averaged measures tend to produce higher scores because they aggregate true positives, false positives, and false negatives across all classes, meaning that the performance on majority classes contributes most strongly to the final value. In contrast, macro-averaged metrics treat each class equally by averaging class-specific results, making them more sensitive to poor performance on minority or rare disease categories. Therefore, macro-averaging is often preferred in medical diagnostic reporting when consistent performance across all disease subtypes is required, since micro-averaging may mask clinically important errors in underrepresented classes.

Weighted averaging offers another aggregation of per-class classification for multi-class classification. Unlike macro-averaging, which assigns equal weight to all classes, weighted-averaging computes class imbalance based on per-class scores weighted by actual occurrences (support) of each class. The result is that classes with a lot of occurrences contribute more to the overall average score, and smaller classes contribute proportionally less [17,25].

The purpose of weighted precision, weighted recall, and weighted F_β_ is to correct the class imbalance present in a data set. The class imbalance problem is resolved using these metrics in three distinct ways. First, the base metric precision, recall, or F_β_ is derived class-wise. Second, every class score is scaled by the support of the class, which is defined as the total count of the true instances of that class. Third, these weighted scores are aggregated to calculate the total weighted average metric [17,25].

Weighted averaging calculates the metric for each class and then finds their average, weighted by the number of instances in each class. This approach provides a score that reflects the model’s overall performance but gives more weight to its performance on more common classes. This is typically what you are looking for if you are interested primarily in the model’s overall accuracy or utility over the full dataset, and the balance of classes is reflective of that of the real-world problem. For instance, over a big image-classification problem where classes of objects occur much more often than others, a weighted average F_β_ scoring would be a truer indicator of how well the model has done overall over most of the examples. A high-weighted-average figure is potentially masking poor minority-class performance, though, if these are not positively weighted [17,25].

In any class of classification, a model may be evaluated using precision, recall, and F_β_ metrics, and associated weights. Here, each class allocation is proportional to the class population in the dataset. For such class allocation, the weighted precision, recall, and F_β_ scores are defined in Equation (8), Equation (9), and Equation (10), respectively.

This method also makes certain that the aggregated metrics are dominated by the weighted average precision and recall of the larger classes, thus yielding a more accurate estimation of the model performance [17,25].

(8) $$P_{weighted}=\sum_{i=1}^{C}\left(P_{i}\times \frac{N_{i}}{\sum_{j=1}^{C}N_{j}}\right)$$

(9) $$R_{weighted}=\sum_{i=1}^{C}\left(R_{i}\times \frac{N_{i}}{\sum_{j=1}^{C}N_{j}}\right)$$

(10) $${F_{\beta }}_{weighted}=\sum_{i=1}^{C}\left({F_{\beta }}_{i}\times \frac{N_{i}}{\sum_{j=1}^{C}N_{j}}\right)$$

These documents have been comprehensively categorized and organized into the following distinct classes: Sports, Politics, and Technology. Evaluating the documents, class-level precision, recall, and F_1_ scores were in the interval between 0.75 and 0.89, recording higher performance for Technology and lower for Sports. Overall class metrics were precision ≈ 0.87, recall ≈ 0.84, and *F*_1_ ≈ 0.85.

The difference between class-level and weighted metrics is due to how class distribution is structured. Technology was the dominant class in the sample, so its stronger performance raised the weighted class averages, while the performance on Sports was barely noticed. This is an excellent case to demonstrate that weighted averages can provide useful single-figure proxies for overall performance but can mask the performance on the smaller classes. This shows they should be used with caution and always supplemented with per-class or macro-averaged scores.

#### 2.1.2. Confidence Intervals

A confidence interval is a range within which a population parameter is likely to lie. It goes beyond a single estimation by providing additional lower and upper limits that a true value may fall under, within a specified confidence level. It has emerged as a central focus within inferential statistics since it allows one to estimate the entire population from a sample [26]. The general form of a confidence interval is expressed as Equation (11) [27].

*Estimate ± (Threshold Value × Standard Error of the Estimate)* (11)

A Z-distribution shown in Equation (12) is used when the sample size (*n*) is large enough, typically n is greater than or equal to 30, or when the population standard deviation (*σ*) is known. When using this type of distribution, several factors are crucial for the confidence interval computation, including the sample mean ($\bar{x}$), the population standard deviation (*σ*), the critical Z-value given for that confidence level $Z_{\frac{a}{2}}$, and total sample size (*n*) [27].

(12) $$CI=\bar{x}\pm Z_{\frac{\alpha }{2}}(\frac{\sigma }{\sqrt{n}})$$

In a situation where the sample size is less than thirty and the standard deviation of the population is not known, one utilizes the t-distribution. In Equation (13), the estimation of the confidence interval relies on a few factors, namely, the sample mean and the corresponding critical t-value, which is based on the level of significance chosen and the sample size, as well as the degrees of freedom (*df* = *n* − 1). Furthermore, the confidence level of interest, as well as some other components, are needed too; in this case, the sample standard deviation (*s*) must also be incorporated [27].

(13) $$CI=\bar{x}\pm t_{\frac{\alpha }{2},df}(\frac{s}{\sqrt{n}})$$

Analytical (Wald) formulas for proportions for classification measures in machine learning like Precision, Recall, and F_β_-score are unreliable, especially for small sample sizes or extreme proportions. To avoid these complications, therefore, the top technique for creating confidence intervals is often the Bootstrap Method. This bootstrapping approach is not based on an interval formula but rather on generating distribution for the statistic that is derived from the data itself through bootstrapping [27].

Percentile method:

B (e.g., 1000 to 10,000) bootstrap replicates of the target measurement are obtained (e.g., these B values are ordered from smallest to largest). For a (1 − α) × 100% confidence interval, the lower bound of the value is the (α/2) × 100th percentile, and the upper bound of the value is the (1 − α/2) × 100th percentile of the bootstrap values [28].

In a set of 100 patients, a model generated 30 true positives, 10 false negatives, 5 false positives, and 55 true negatives. Its F_1_ score was approximated to 0.80. A 95% confidence interval was bootstrapped using 1000 resamples, yielding (0.74, 0.85).

This interval captures the F_1_ score’s estimation uncertainty, related to sample randomness, and allows for a better evaluation of the model compared to using F_1_ as a point estimate. Such confidence intervals are crucial in medicine as data are scarce and the accuracy of the interval estimate is of utmost importance.

Empirical confidence intervals can be estimated using resampling methods (especially bootstrap) by repeatedly sampling the dataset and recalculating performance metrics. Confidence intervals can then be computed using percentile bootstrap or BCa bootstrap, and similar uncertainty estimates can also be obtained through repeated cross-validation, which is useful when sample sizes are small or distribution assumptions are unclear [29].

#### 2.1.3. Confusion Matrix

The confusion matrix is a key tool in evaluating the performance of classification models, aiding in a more detailed analysis of an algorithm’s prediction results. This matrix presents a two-dimensional table that outlines the number of correct and incorrect predictions for each class, allowing us to assess how well the model has performed in identifying data. This tool helps us calculate the overall accuracy of the model while also identifying its strengths and weaknesses. By analyzing this matrix, we can gain valuable insights into the types of errors and patterns of incorrect predictions, which can lead to continuous improvement of the model and increased efficiency in real-world applications. Ultimately, the confusion matrix is a visual and useful tool that allows us to clearly and understandably present the algorithm’s performance and make more informed decisions (Table 1) [30].

The confusion matrix is better than a single metric because by comparing multiple metrics, it allows for a detailed analysis of the performance of classification models and helps identify or fix weaknesses. The confusion matrix also shows which classes are better recognized and what types of errors occurred [31,32].

A confusion matrix is a simple table that displays the relationship between actual and predicted classes. It consists of the following four key components: true positive (TP), which refers to samples that are genuinely positive and correctly identified as such by the model; true negative (TN), indicating samples that are truly negative and accurately recognized as negative by the model; false positive (FP), which denotes samples that are actually negative but incorrectly classified as positive by the model, representing a Type I error; and false negative (FN), referring to samples that are genuinely positive but mistakenly identified as negative by the model, illustrating a Type II error [33].

**Figure 2:**
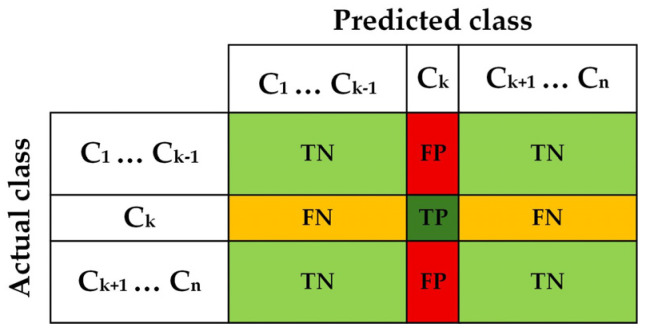
n × n confusion matrix.

The confusion matrix can be used in both binary problems (such as cancer/no cancer) and multi-class problems (such as predicting the type of animal, type of flower, or quality grade) (Figure 2) [33].

Some other scale including prevalence, accuracy, positive predictive value, false discovery rate, false omission rate, negative predictive value, true positive rate, false positive rate, false negative rate, true negative rate, positive likelihood ratio, negative likelihood ratio, diagnostic odds rate, and F_1_ score can also be calculated using the four main parameters of the confusion matrix table, which are fully mentioned in Table 2 [32].

**Table 2:** Confusion matrix table [32].

	Condition Positive (D)	Condition Negative (D_c_)	Clinical Interpretation Focus
Prediction Positive (E)	True Positive (TP)	False Positive (FP) (Type I error)	Positive test result
			PPV (Precision) = TP/(TP + FP)
			FDR = FP/(TP + FP)
Prediction Negative (E_c_)	False Negative (FN) (Type II error)	True Negative (TN)	Negative test result
			NPV = TN/(TN + FN)
			FOR = FN/(TN + FN)
Column-derived measures	Sensitivity (TPR) = TP/(TP + FN) FNR = FN/(TP + FN)	Specificity (TNR) = TN/(TN + FP) FPR = FP/(TN + FP)	Discrimination ability
Population-level measures			Accuracy (ACC) = (TP + TN)/N Prevalence (Prev) = (TP + FN)/N
Likelihood-based measures			LR+ = Sensitivity/(1 − Specificity) LR− = (1 − Sensitivity)/Specificity
Combined diagnostic strength			Diagnostic Odds Ratio (DOR) = (TP × TN)/(FP × FN)
Harmonic summary			F1-score = 2TP/(2TP + FP + FN)

#### 2.1.4. Statistical Analysis of the Validation Measures

Measures drawn from the confusion matrix such as sensitivity, specificity, and predictive values provide the foundation for assessing diagnostic and predictive systems. Yet these types of measures tend to be limited to data in question and cannot truly represent how a model will act when it is actually deployed in real populations. To bridge this shortcoming, statistical evaluation models move beyond the simple true and false counting of classifications and substantively take into consideration the prevalence of the condition. This allows for performance to be grasped in samples under control but also in actual settings; for example, where disease rarity or prevalence influences predictive reliability in a disease detection model [34].

The four basic outcomes of classification that were explained in previous sections can be formulated probabilistically in terms of conditional probability and prevalence of disease. Tree diagram representation leads to the following equations below. *D* and *E* were explained in Table 2.

(14) $$TP=P\left(ED\right)\times S=P(E\backslash D)\times P(D)\times S$$

(15) $$TN=P\left(E^{c}D^{c}\right)\times S=P(E^{c}\backslash D^{c})\times P(D^{c})\times S$$

(16) $$FP=P\left(ED^{c}\right)\times S=P(E\backslash D^{c})\times P(D^{c})\times S$$

(17) $$FN=P\left(E^{c}D\right)\times S=P(E^{c}\backslash D)\times P(D)\times S$$

Here, *P*(*D*) is the prevalence of disease among the population, *P*(*D^c^*) = 1 − *P*(*D*), and *S* is the size of the population. Sensitivity and specificity arise naturally as conditional probabilities [34].

(18) Se=P(E\D)

(19) $$Sp=P(E^{c}\backslash D^{c})$$

Although sensitivity and specificity quantify how effectively a test distinguishes diseased from non-diseased individuals, they do not provide the clinically crucial information of how likely it is that a patient with a positive test result truly has the disease. This posterior probability, *P*(*D*\*E*), is obtained through Bayes’ theorem (Equation (20)).

(20) $$P(D\backslash E)=\frac{P(E\backslash D)\times P(D)}{P(E\backslash D)\times P(D)+P(E\backslash D^{c})\times P(D^{c})}=\frac{Se\times P}{Se\times P+\left(1-Sp\right)\times \left(1-P\right)}$$

The posterior probability is the positive predictive value or precision. PPV varies from sensitivity and specificity in that it varies substantially with prevalence. For instance, a test with *Se* = 0.80 and *Sp* = 0.95 has a posterior probability of true disease among individuals with a positive test of about 64% at 10% prevalence. Even with a very sensitive and specific test *Se* = 0.99 and *Sp* = 0.99, if prevalence is as low as 0.5%, then posterior probability is still only 33%. These examples show that mentioning high sensitivity and specificity is not sufficient; prevalence affects predictive value in the real world.

Although sensitivity and specificity provide important information about discrimination, clinical usefulness also depends on disease prevalence, downstream confirmatory testing, and the relative harms of false-positive and false negative decisions. Instead, acceptable operating performance must be defined in relation to the intended clinical role of the model (e.g., screening, triage, or confirmatory diagnosis), the expected prevalence in the target population, and the consequences of diagnostic error. In practice, models with seemingly strong global metrics may still be clinically inadequate if they perform poorly at the required decision threshold or if predictive values become unacceptable under low-prevalence conditions.

Because of such dependence, complementary measures have been developed. One of these is the diagnostic odds ratio (DORs), which is the odds ratio of a diseased individual having a positive test to a non-diseased person (Table 2). The DOR reduces discriminative performance to a single metric and, importantly, is disease prevalence-independent. It is a robust metric of model strength between groups and has been heavily proposed as an across-the-board measure of assessment [34].

By uniting conditional probabilities with prevalence, statistical evaluation methods go beyond dataset-limited confusion matrices to characterize the performance of diagnostic or predictive systems in true populations. Such a perspective is essential for introducing machine learning or clinical models into practice, where prevalence is not constant, patient heterogeneity, and cost of error determine whether an instrument has meaningful utility outside controlled testing conditions.

### 2.2. Multi-Label Classification Models

Unlike traditional classification tasks that predict or estimate just one label or class for each sample, the multi-label classification approach allows for several labels to be applied at once, even if those labels are correlated. This is a method of explaining the research question and then using ML, DL, or AI models to execute it. In the input dataset, each sample is paired with a label vector, which is a coded format that shows which labels are relevant for that particular sample. Then, multi-label classification models are trained on the dataset to predict or estimate label vectors based on the input features. Such models are widely used in various domains, including medical diagnosis, text categorization, speech type recognition, and image classification based on the objects present [6]. For example, in chest X-ray image analysis, the existence or non-existence of detectable diseases in each patient is represented by a vector in the following format:

Labels vector: [Pneumonia, Tuberculosis, Cardiomegaly, Pleural Effusion]

Patient #1: [1, 0, 1, 0]

Evaluation metrics of multi-label models differ from traditional classification approaches. The next sections explain the methods used for evaluating the performance of multi-label classification models, as mentioned in Table 3 [7].

#### 2.2.1. Hamming Loss

Hamming loss is one of the most common metrics for evaluating multi-label classification models. This metric measures the percentage of prediction error (labels that are incorrectly predicted) or missing error (labels that are not predicted at all) for each sample. Importantly, this metric reflects the overall model performance in making errors, regardless of any specific sample or label [35].

(21) $$Hamming\;Loss=\frac{1}{N}\sum_{i=1}^{N}\frac{\left|Y_{i}∆Z_{i}\right|}{L}$$

This metric returns the number of mismatches between the predicted labels (*Z_i_*) and the ground-truth labels (*Y_i_*) by function ∆ for each sample *i* and then measures the fraction of these mismatches over the total number of labels *L* and finally averages this value over all *N* samples.

This metric calculates the ratio of misclassified labels to the total number of labels across all samples [36]. For example, in Table 4, four labels are incorrectly predicted, regardless of which sample or label they belong to. Therefore, the hamming loss will be 0.5 for this model example with *N* = 2 and *L* = 4, which means the model cannot predict 50% of labels correctly. A lower value indicates better model performance.

#### 2.2.2. Exact Match Ratio

The exact match ratio, also known as Subset Accuracy, is the fraction of samples where all predicted labels exactly match the ground-truth labels. This metric is very rigid, especially when the number of possible labels is large. In such cases, it becomes increasingly complex to find samples that are completely correctly classified, as both completely misclassified samples and those that are almost correct are considered incorrect [35].

(22) $$Exact\;Match\;Ratio=\frac{1}{N}\sum_{i=1}^{N}I(Z_{i}=Y_{i})$$

*I* is the indicator function that returns 1 if the predicted label set *Z_i_* and ground-truth label set *Y_i_* are exactly matched, and 0 otherwise. This metric is computed as the ratio of all these values to the total number of samples (*N*). If the indicator function counts the opposite condition (returns 1 when the predicted label set does not exactly match the ground-truth label set), then the resulting metric is known as the Subset 0/1 Loss [36].

(23) Subset Loss=Exact Match Ratio−1

For example, in a study similar to Table 5, only 1 out of 4 predicted label sets exactly match the ground-truth labels. Therefore, the exact match ratio (Subset Accuracy) in this case would be 0.25. This highlights the strictness of this metric, as even minor deviations in label predictions for a single sample are not acceptable.

#### 2.2.3. Jaccard Index

The Jaccard Index, also known as Intersection over Union (IoU), measures the similarity percentage between the predicted and ground-truth labels for each sample and over all samples. Unlike strict metrics such as Exact Match Ratio, the Jaccard Index rewards partial correctness. This makes it especially useful in real-world applications where complete agreement between predicted and ground-truth label sets may be rare, but partial matches are common and still meaningful [37].

(24) $$Jaccard\;Index=\frac{1}{N}\sum_{i=1}^{N}\frac{\left|T_{i}\cap P_{i}\right|}{\left|T_{i}\cup P_{i}\right|}$$

This metric computes the number of matched labels between the predicted label set *P_i_* and the ground-truth label set *T_i_* for sample *i*, and then the ratio of all these values to the total number of labels. For example, in Table 6, if 3 out of 4 labels are correctly predicted for sample #1, then the Jaccard Index would be 0.75. Overall, the Jaccard Index for the entire dataset is the average of the Jaccard indices of all samples (*N*). IoU with higher values indicates better partial agreement.

#### 2.2.4. Distribution Difference

Distribution Difference or Distance Metrics are label-based evaluation metrics that assess how well each label is predicted across the entire dataset (Table 7). These metrics are beneficial for examining distributional fairness or balance among the labels, showing whether the model has learned to predict each label fairly well, and measuring how much the distribution of predicted labels deviates from the true distribution [37].

## 3. Evaluation Representation Curves

A model’s performance is sometimes more intuitively and better appreciated if graphically defined as opposed to just spelt out numerically. What follows below is how the employment of curves can provide a more intuitive and better comprehension of the performance of a model. The visual aids allow us to make intelligent guesses regarding model behavior at different thresholds and see how the model can recognize true positives and reject false positives. In addition to examining one-value metrics, these curves allow us to examine patterns, trade-offs, and overall trends in performance.

### 3.1. Precision–Recall Curve (PRC)

The precision–recall curve shows the relationship between recall and precision across different decision-making inputs. Precision or positive predictive value (Equation (25)) is the number of instances that the model predicted to be positive that are actually positive, which is obtained by dividing the true positive (TP) rate by the sum of the true positive and false positive (FP) rates. Recall or true positive rate is a measure of the percentage of actual positive samples that the model also correctly predicted as positive, which is obtained by dividing the true positive rate by the sum of the true positive and false negative (FN) rates (Equation (26)) [38].

(25) PPV=TP/(TP+FP)

(26) TPR=TP/(TP+FN)

The precision–recall curve shows the relationship between recall and precision for various decision inputs. Precision is the number of samples that the model predicted to be positive and are actually positive, which is obtained by dividing the true positive rate by the sum of the true positive and false positive rates. Recall is a measure of the percentage of true positive samples that the model also correctly predicted to be positive, which is obtained by dividing the true positive rate by the sum of the true positive and false negative rates.

**Figure 3:**
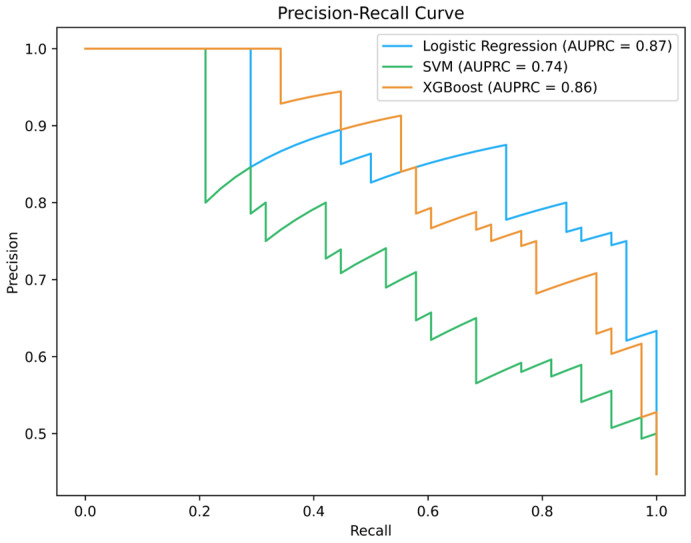
Precision–recall curves for Logistic Regression, SVM, and XGBoost models on the CAD dataset.

The formulas above obtain precision and recall at different output thresholds. These values are points on the axis to create the PR curve; the length of the axis is called recall, and the width of the axis is called precision. The method of measuring this curve is by calculating the area under the PR graph, which is called average precision. The value of this parameter is in the range from 0 to 1. The value of this parameter ranges from 0 to 1, so that the closer it is to 1, the better the model performance. This parameter displays the precision–recall curve at a single value, which represents the sum of the precisions at different recall values. Figure 3 illustrates precision–recall curves applied to the coronary artery disease (CAD) dataset [39]. The plot contains three PR curves corresponding to Logistic Regression, SVM, and XGBoost models. The curves show that Logistic Regression outperforms XGBoost, which in turn performs better than SVM.

In this study, a publicly available clinical dataset related to coronary artery disease (CAD) diagnosis was used to evaluate the proposed analysis and visualization framework. The dataset was obtained from the Cleveland Heart Disease database, which is hosted by the University of California Irvine (UCI) Machine Learning Repository. This dataset has been widely used as a benchmark in cardiovascular disease prediction and clinical decision-support studies. The original dataset contains 303 patient records; however, after excluding samples with missing values, a total of 272 complete patient records were retained for analysis. The study population consisted of approximately 68% male subjects, and the diagnostic ground truth was derived from coronary angiography results, where CAD status was defined based on the narrowing of at least one coronary artery exceeding 50% [39].

The dataset includes demographic, lifestyle, and clinical examination variables that are routinely collected in medical practice and are clinically relevant to CAD screening. The recorded features include age (years), sex, chest pain type (cp), resting systolic blood pressure (trestbps), serum cholesterol (chol), cigarettes per day (cigs), number of years as a smoker (years), fasting blood sugar (fbs), family history of CAD (famhist), resting electrocardiographic results (restecg), maximum heart rate achieved (thalach), resting heart rate (thalrest), peak exercise systolic and diastolic blood pressure (tpeakbps, tpeakbpd), resting diastolic blood pressure (trestbpd), exercise-induced angina (exang), ST depression induced by exercise (oldpeak), slope of the peak exercise ST segment (slope), number of major vessels colored by fluoroscopy (ca), and thallium-201 stress scintigraphy (thal). Additionally, the dataset provides the diagnostic label as both a categorical disease indicator (num) and a binary outcome variable (outcome) representing the presence or absence of angiographic CAD [39].

To support transparency and reproducibility of the reported results, the dataset used in this study, along with the complete source code developed for preprocessing, statistical analysis, and figure generation, has been made publicly available through an open-access GitHub repository. The repository includes Python-based (IPvthon version 8.14.0) Jupyter Notebook scripts (.ipynb) that were used to generate the plots and visualizations presented in this manuscript, in addition to documentation describing the dataset structure and feature definitions. The GitHub (version 2.41.0) repository can be accessed at https://github.com/mrzakariarajabi/heart-disease-dataset-visualization-CAD- (accessed on 17 February 2026).

The space under the precision–recall curve is the average precision (AP) and its formula is Equation (27) [38].

(27) $$AP=\int_{0}^{1}p\left(t_{i}\right)dr(t_{i})$$

The AP formula using the trapezoidal rules is in Equation (28) [38].

(28) $$AP=\sum_{i=1}^{k}p\left(t_{i}\right)[r\left(t_{i}\right)-r\left(t_{i-1}\right)]$$

with r(t_0_) = 0.

In the process of disease prediction and diagnosis, the setting of the decision threshold plays a crucial role in determining the model’s performance. At high thresholds, the model declares only people who have obvious symptoms of the disease as sick, and the diagnoses are approximately correct, and the accuracy of the model is high. However, a percentage of patients who have few symptoms but are sick may not be identified, so the recall is low. At low thresholds, the model identifies patients with greater sensitivity. As a result, more real patients are identified, indicating high recall, but the accuracy of diagnosis is reduced due to the misdiagnosis of healthy individuals. The precision–recall curve at different thresholds creates a balance between precision and recall. This curve refers to the interaction between precision and recall.

### 3.2. Lift Curve

Lift is a parameter that measures the success of a model in predicting a subject (being sick) compared to a control model. The lift coefficient when the class is positive is in Equation (29) [38].

(29) $$lift\left(y=1\right)=P\left(y=1\right|\hat{y_{c}}=1)/P(\hat{y_{null}}=1)$$

**Figure 4:**
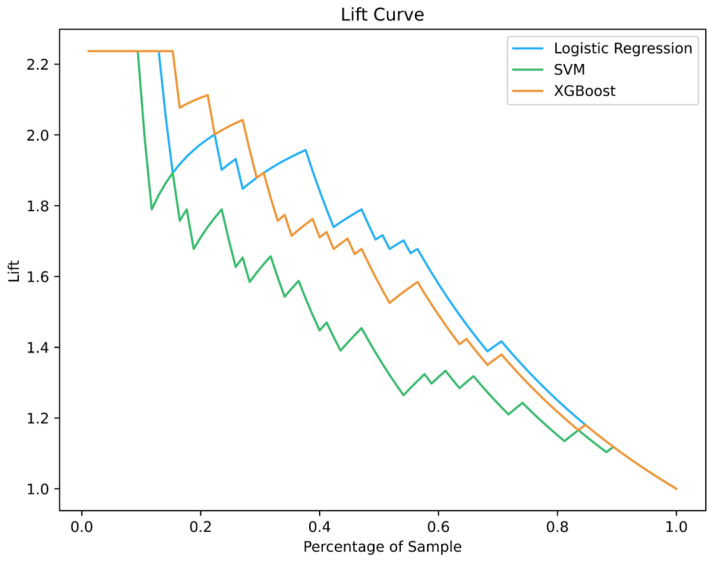
Lift curves for Logistic Regression, SVM, and XGBoost models on the CAD dataset.

The lift coefficient compares our predictions using our actual model to a random guess that could be made on the entire population or a specific control group; it tells us how much better our predictions would be if the actual model is used. The greater the lift, the better the model performs than chance. In lift curve, the horizontal axis of this curve is the percentage of positive predictions in the entire data at several thresholds, and the vertical axis is the ratio of the true positive rate between the model and a random classifier (the lift value). The lift curve shows the performance of the model relative to random guesses on the data. Figure 4 shows the lift curves for Logistic Regression, SVM, and XGBoost models applied to the CAD dataset [39]. The results demonstrate that Logistic Regression achieves the highest performance, followed by XGBoost and then SVM.

Suppose a neural network-based model is designed to detect the presence of a cancerous tumor in MRI images. The model has high discrimination power when it correctly identifies patients, and the model’s performance is several times better than random guesses. The higher the value of the lift parameter, the greater the model’s ability to identify real patients and distinguish them from healthy individuals.

### 3.3. Cumulative Response Curve (CRC)

**Figure 5:**
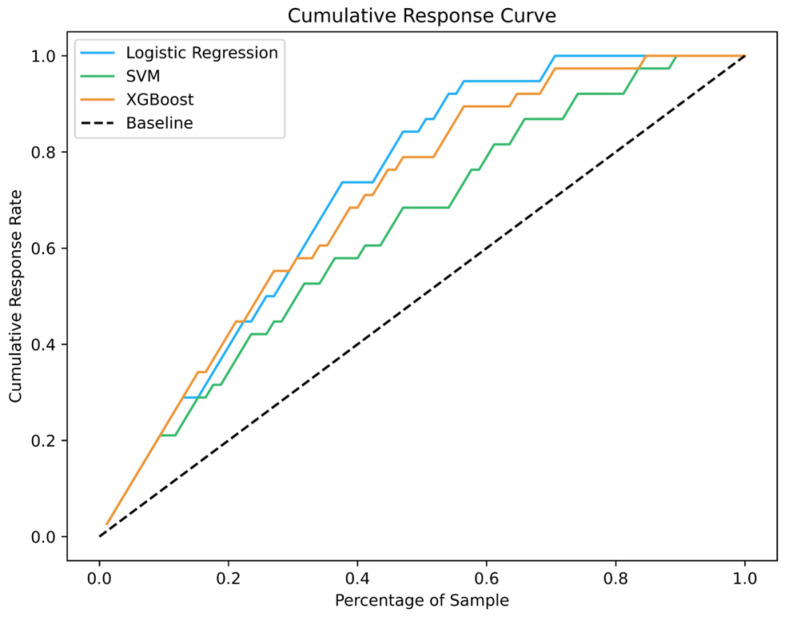
Cumulative response curve (CRC) comparison of Logistic Regression, SVM, and XGBoost models applied to the CAD dataset.

The cumulative response curve or cumulative gain curve is a graph that evaluates the performance of a binary classification system when faced with different decision threshold values. The true positive rate against different threshold levels forms this diagram. In this diagram, each binary classifier is represented as a point for each threshold, and by changing the threshold in each classification, a set of these points is formed in that classification, which indicates the behavior of the model at different sensitivity levels. The cumulative gain curve is a graph that shows what percentage of all positive samples are identified by examining different data thresholds. The form and location of the gain curve depend on the accuracy of the model’s performance, as well as the proportion of positive samples to the entire sample [40]. Figure 5 cumulative response curves (CRCs) for Logistic Regression, SVM, and XGBoost models on the CAD dataset [39]. The curves demonstrate that Logistic Regression provides superior performance, with XGBoost performing better than SVM.

For example, suppose a medical school has a machine learning model to diagnose patients with (1) or no (0) colon cancer. The model gives each patient a probability of having cancer from 0 to 1 based on the patient’s status. Let us say the patients are ranked from highest to lowest based on this probability. The model is successful in correctly identifying a percentage of patients. In the random guess and without the model, the percentage of patients correctly identified decreases. The difference between the two cases represents the gain. The higher the gain, the better the model performs compared to the random case.

### 3.4. Receiver Operating Characteristic (ROC)

The ROC curve is a graph that can compare the rate of correct identification of positive cases (true positive rate) versus the rate of incorrect identification of negative cases as positive (false positive rate) and evaluate the performance of the model (binary). The sensitivity and false positive rate values are calculated and tabulated for different thresholds to generate the ROC curve. The y-axis of the ROC curve represents the sensitivity or true positive rate (Equation (30)) [38], and the x-axis of this curve represents the false positive rate (FPR = 1 − specificity) (Equation (31)) [41].

(30) TPR=TP/(TP+FN)

(31) FPR=FP/(FP+TN)

AUROC evaluates the power of a model in distinguishing positive and negative classes. The area under the ROC curve (AUC) is a comprehensive measure of the overall accuracy of a model in distinguishing between cases with and without a particular disease; this value is calculated by integrating the points on the ROC curve and has a value between 0 and 1; the closer this value is to 1, the better the model performs. AUC in the range of 0 to 0.5 indicates a model without the power of distinction, and its performance is similar to random guessing, but the higher the AUC is than 0.5 and closer to 1, the more the model achieves the power of distinction between classes completely [41]. AUROC is useful when measuring the ability of a model to correctly rank samples, but it is not appropriate when the number of samples in the positive and negative classes is very different or when one of the positive or negative classes is more important. The AUC formula is in Equation (32) [38].

(32) $$AUC=\int_{0}^{1}TPR\left(t_{i}\right)dFPR(t_{i})$$

**Figure 6:**
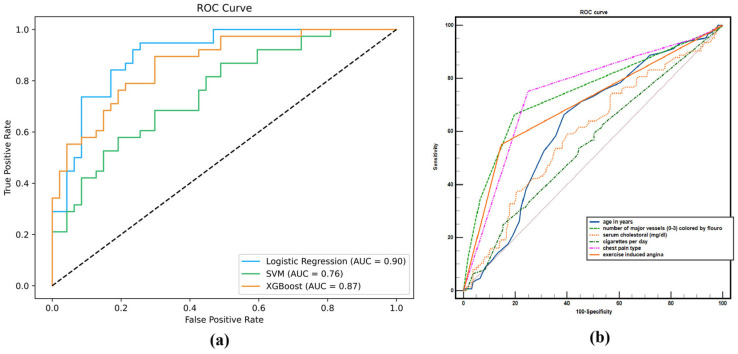
(**a**) ROC curves for Logistic Regression, SVM, and XGBoost on the CAD dataset. (**b**) ROC curves from Logistic Regression with selected CAD variables, including AUC and pairwise comparisons.

Figure 6a illustrate receiver operating characteristic (ROC) curves for Logistic Regression, SVM, and XGBoost models on the CAD dataset [39]. The results indicate that Logistic Regression achieves the best performance, followed by XGBoost and then SVM. Furthermore, another important application of ROC analysis is the evaluation of the relative importance of input variables. MedCalc software (version 23.3.7) facilitates this process by enabling the comparison of variables through two established methods (DeLong et al., [42]; Hanley & McNeil, [4]). For each variable, the software provides the area under the curve (AUC), standard error (SE), and 95% confidence interval (CI). In addition, MedCalc performs pairwise comparisons of ROC curves between variables, reporting the difference between areas, standard error, 95% CI, z-statistic, and significance level [43]. Figure 6b presents ROC curves derived from Logistic Regression using selected variables of the CAD dataset [39].

For example, a doctor has a computer model that can distinguish between patients with a certain disease and healthy patients. The model gives each patient a probability score. At a high decision threshold, only the patients with the highest probability are declared positive, meaning that almost everyone who is diagnosed is actually sick, but many real patients are not identified. At a low decision threshold, everyone with a moderate or high probability is declared sick, meaning that most real patients are identified, but at the same time, some healthy people are misdiagnosed. The ROC curve represents the proportion of real patients identified and the proportion of healthy people misdiagnosed. By changing the threshold to different values and calculating the FPR and TPR parameters, the ROC curve is plotted, and then the area under the curve is obtained, yielding the AUROC. The closer the curve is to the upper-left corner, the better the model is at identifying real patients and the fewer false positives will it produce.

### 3.5. Measurement Plots

To report the metrics and visualization used to assess model performance, researchers constructing or validating clinical prediction models should explicitly state all performance metrics and visualizations used, and the rationale for their selection. These include discrimination metrics (e.g., AUC, c-statistic), calibration metrics (e.g., calibration slope, Brier score), and clinical utility metrics (e.g., decision curve analysis). If more than one model is being contrasted, the grounds on which they are being contrasted should be explicitly stated.

**Figure 7:**
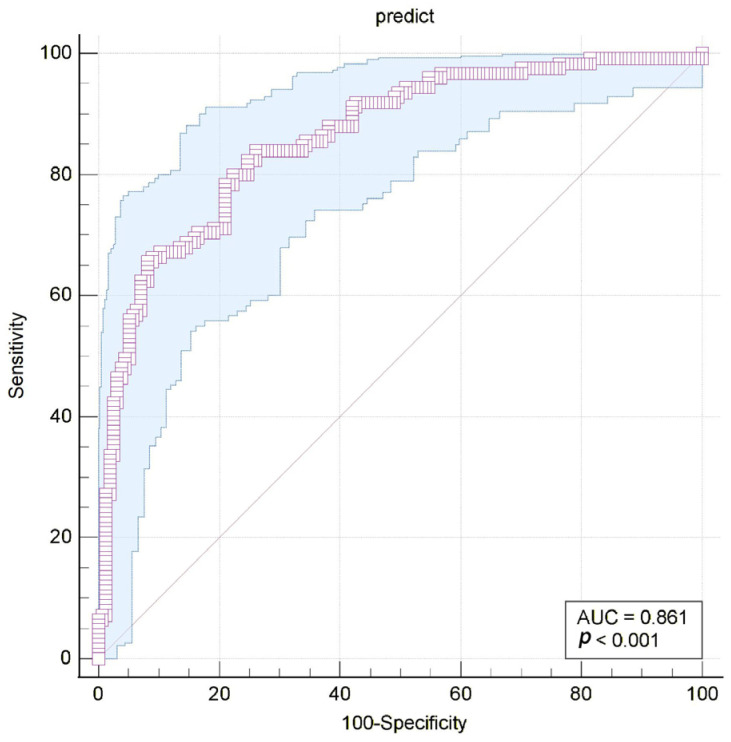
The dashed line represents no discrimination (AUC = 0.5). The area under the curve is above 0.8, which means excellent discriminative ability. The shaded blue region represents the 95% confidence interval around the ROC curve.

Discrimination metrics such as the area under the curve (AUC) quantify a model’s ability to discriminate between two outcome classes. This curve plots the true positive rate against the false positive rate across a range of threshold values. As in Figure 7, the value range of this measurement is from 0 to 1, and it is calculated by determining the area under the curve. A value of 0.5 indicates that the model performs no better than a random chance, while a value of 1.0 represents perfect discrimination.

Calibration metrics assess the correspondence between a model’s probabilistic predictions and the observed outcomes. For example, the calibration slope is achieved through the implementation of a logistic regression model. Within this model, the observed binary outcome (0 or 1) is regressed on the log odds of the output probabilities from the original model using Equation (33).

(33) $$Outcome\sim \beta_{0}+\beta_{1}\times log\left(\frac{p}{1-p}\right)$$

**Figure 8:**
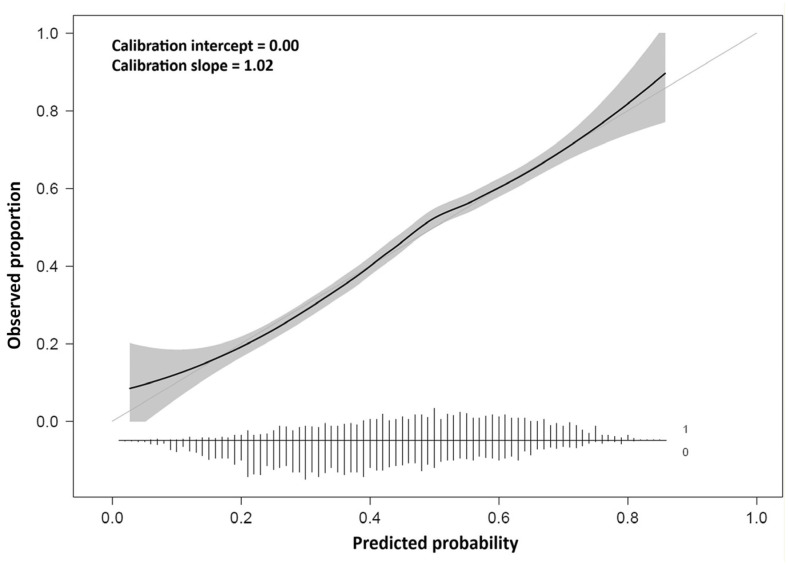
The calibration plot: a slope value of one suggests perfect calibration, meaning that the predicted probabilities correspond well with the observed outcomes [15]. The solid black line represents the estimated calibration curve, while the surrounding gray shaded region indicates the 95% confidence interval around the calibration estimate.

Here, *p* denotes the predicted probability from the original model, *β*_0_ is the calibration intercept for reflecting systematic over- or underestimation of risk, and the coefficient $\beta_{1}$ is the calibration slope [15]. A slope value above one indicates underfitting, while a slope value below one suggests overfitting. As illustrated in Figure 8, perfect calibration is represented by the 45° diagonal line, where predicted probabilities equal observed risk.

Another approach to model calibration is to use the Brier score. It measures how well a forecast is calibrated and how accurately it predicts the chance of an event. It is calculated by averaging the difference between predicted and actual outcomes. The score is expressed using Equation (34).

(34) $$Brier\;score=\frac{1}{N}\sum_{i=1}^{N}{\left(f_{i}{-E}_{i}\right)}^{2}$$

where E is the actual outcome (0 or 1), f is the predicted probability for i in total N observations [6]. The possible values range from 0 to 1, with a value close to zero indicating better model performance, and a score of 1 indicating completely incorrect decisions. In addition to calibration, this metric evaluates how sophisticated the model is at predicting events versus non-events. For example, in 5 patients, with a mean squared difference in predicted values and actual outcomes of 0.46, the score is 0.092, which is relatively low, meaning that the predictions are close to the existing results.

A decision curve analysis evaluates the usefulness of a model in a clinical setting by weighing the benefits of correct predictions against the harms of incorrect ones. It is calculated as shown in Equation (35).

(35) $$Net\;benefit=\frac{TP}{n}-\frac{FP}{n}\times \frac{p_{t}}{1-p_{t}}$$

where TP and FP represent the number of true and false positives, n is the number of individuals, and $p_{t}$ is the probability threshold [2]. The probability threshold is the risk level at which a decision maker (e.g., a doctor) chooses to treat or take an action. Suppose the model predicts 20 true positives and 10 false positives using 100 patients. If our probability threshold is 0.10, the Net benefit is 0.189. This means identifying about 18.9 true positives per 100 patients without unnecessary treatment is equivalent.

These metrics make reproducibility, critical analysis, and potential safe clinical application of AI prediction models feasible. For model performance in prognostic models, it must be tested for all relevant time points. Also, graphical presentations such as calibration plots and decision plots must be included to explain the model. When models are being compared to multiple models, the criteria for comparison and basis for superiority claim must be defined.

### 3.6. Bland and Altman’s Analysis

Bland and Altman’s analysis is a technique for comparing two measurements or instruments designed to measure the same quantity. Based on agreement limits, it develops a method for quantifying the agreement between two quantitative measurements [44]. The agreement limits are computed using the mean and standard deviations of differences using Equation (36).

(36) Agreement limit=mean differences±1.96×standard deviation

**Figure 9:**
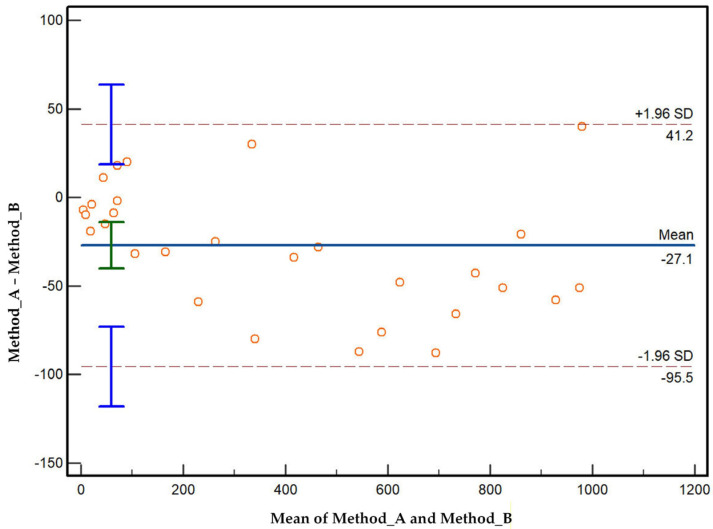
A Bland and Altman plot with hypothetical data done by methods A and B. The error bars present 95% confidence interval limits for mean and agreement limits. The orange circles represent individual paired measurements, the solid blue line indicates the mean difference, and the dashed red lines denote the ±1.96 standard deviation limits of agreement.

The points in Figure 9 at low mean values (differences between Method_A and Method_B) are primarily dispersed around zero. In this range, two methods sometimes agree but variability is still present. At higher mean values (>400), the differences become negative and fall below the mean line. This downward trend indicates possible proportional bias. Upper limit of agreement (LoA) is +41.2 and lower LoA is 95.5. Therefore, in most cases, method A could measure 95 units lower and 41 units higher than method B. On average, method A gives values 27 units lower than method B as shown in mean line. Overall, the figure indicates poor agreement between methods; wide LoA suggests that methods are not interchangeable without correction.

## 4. Bias, Fairness, and Robustness Assessment

In high-stakes medical and clinical applications, concepts such as fairness, bias, and robustness must be defined operationally and evaluated using testable procedures rather than discussed only at a conceptual level [8,12]. In this study, these terms are treated as measurable properties of the predictive system, each associated with specific evaluation protocols. Fairness focuses on whether the model performs consistently across sensitive subgroups, bias refers to systematic distortions introduced by data or modeling choices, and robustness assesses the stability of model performance under controlled perturbations of the input data.

### 4.1. Bias and Fairness Assessment

Fairness assessment is employed to determine whether the predictive model exhibits systematically different behavior across predefined sensitive groups, such as gender, age categories, or other clinically relevant demographic attributes [10,45]. Rather than relying solely on aggregate performance metrics (e.g., accuracy or F1 score), fairness analysis evaluates whether error rates and prediction distributions differ between groups [10,46]. A model is considered unfair if it disproportionately disadvantages a particular group through higher false negative rates, higher false positive rates, or systematically different decision thresholds [45].

Bias in this context refers to systematic deviations that cause unequal model behavior across subpopulations [47]. Several types of bias are relevant in clinical datasets [48]. Representative bias occurs when certain groups are underrepresented or overrepresented in the training data, leading to poorer generalization for minority groups [47,49]. Selective bias arises when the dataset includes only a subset of the population due to inclusion or exclusion criteria, potentially skewing the learned decision boundaries [50]. Measurement bias may also be present when clinical variables are recorded differently across groups [51]. Identifying these biases requires stratified analysis of performance metrics and prediction distributions across demographic attributes [10].

### 4.2. Equalized Odds as an Operational Fairness Criterion

To operationalize fairness, this study adopts equalized odds, a well-established fairness criterion in binary classification. Equalized odds require that a classifier achieve equal true positive rates (TPRs) and false positive rates (FPRs) across different sensitive groups [45]. This criterion ensures that the likelihood of correctly identifying a positive case, as well as the likelihood of incorrectly flagging a negative case, does not depend on group membership.

Formally, let $\hat{Y}$ denote the model’s predicted outcome, Y the true outcome, and A sensitive attribute (e.g., sex or age group) [52]. A classifier satisfies equalized odds if:

(37) $$P(\hat{Y}=1|Y=y,A=a)=P(\hat{Y}=1|Y=y,A=b),\;\forall y\in \left\{0,1\right\},\;\forall a,b\in A$$

This condition can be expressed equivalently as:

(38) TPRa=TPRb and FPRa=FPRb

For all sensitive groups a and b, in practical terms, this means that for individuals who truly have the disease (Y=1), the probability of correct detection should be the same across groups, and for individuals without the disease (Y=0), the probability of false alarm should also be comparable [53].

For example, consider a model predicting whether a patient is at high risk for a specific disease. If the model systematically fails to identify affected patients from a particular demographic group (lower TPR) or incorrectly flags healthy individuals from that group as high risk (higher FPR), the model violates equalized odds. Enforcing or evaluating equalized odds helps ensure that the diagnostic system does not unfairly advantage or disadvantage specific populations, thereby improving equity in clinical decision-making.

### 4.3. Robustness Assessment via One-at-a-Time Perturbation

Robustness is defined operationally as the stability of model performance under controlled perturbations of input features [9]. In clinical settings, measurements are often noisy due to device variability, patient movement, or recording errors [54]. A robust model should therefore maintain consistent predictions when small, plausible changes are introduced into the input data [9].

Robustness is evaluated using a one-at-a-time (OAT) perturbation protocol, where noise is injected into individual input features while all other features are held constant. For each feature, Gaussian or bounded noise is added incrementally, and model performance metrics (e.g., accuracy, F1-score, and sensitivity) are recomputed at each noise level [9]. Let δ denote the perturbation magnitude; robustness can then be assessed by monitoring performance degradation as a function of δ [11,55].

A robustness threshold can be defined as the maximum perturbation level for which performance remains within an acceptable deviation (e.g., less than 5% drop in F1-score) [9]. If performance degrades sharply under small perturbations, the model is considered sensitive and potentially unreliable in real-world clinical conditions. Conversely, gradual performance degradation indicates a more robust and trustworthy system [9].

## 5. Performance Metrics and Loss Functions

In clinical machine learning, evaluating predictive performance requires more than reporting a single accuracy-based measure. Depending on whether the model output is continuous (e.g., risk score or biomarker prediction) or categorical (e.g., disease vs. non-disease), different families of metrics are needed to quantify error magnitude, probabilistic uncertainty, and agreement between predicted and true outcomes. In this section, we summarize commonly used error-based measures (e.g., RMSE, MAE), loss functions used during model optimization (e.g., hinge and entropy-based losses), and statistical association and agreement metrics (e.g., correlation coefficients, balanced accuracy, MCC, and Cohen’s kappa). Together, these measures provide complementary perspectives that support more reliable and clinically interpretable assessment of diagnostic AI systems.

### 5.1. Root Mean Square Error

The Root Mean Square Error (RMSE) is a commonly used metric for evaluating the accuracy of an artificial intelligence model and the degree of dispersion in its errors. This metric measures the distance between the observed data and the regression line. So, when absolute deviations are large (i.e., larger errors), the squaring in its formula places greater emphasis on these errors. Consequently, RMSE is more sensitive to error variability. In addition, as the sample size increases, RMSE becomes a more reliable measure of error variability, improving the model’s stability and accuracy. These errors are considered unbiased; in other words, their mean is zero, which means the model, on average, neither underestimates nor overestimates the actual values. The RMSE formula is given in Equation (39) [56].

(39) $$RMSE=\sqrt{\frac{1}{N}\sum_{i=1}^{N}{e_{i}}^{2}}$$

The RMSE formula is used to calculate the error index. The RMSE provides a measure of the average deviation between the model’s predictions and the observed values, quantifying the model’s predictive accuracy. When using RMSE, it is assumed that the errors are normally distributed. It means most errors are small, although a few larger ones may occur. If the number of errors is large (e.g., 100 samples or more), the RMSE value tends to be closer to the actual error, offering a clearer picture of the model’s accuracy. But if the sample size is small, the RMSE value may not be sufficiently precise [56].

### 5.2. Prediction Errors

Many metrics in statistics and machine learning are used to measure prediction errors, especially when dealing with outliers. These metrics quantify the difference between the actual values and the predicted values of an AI model. They are known as loss functions. For example, Max Error is a loss function for indicating the model’s worst error. It calculates the largest difference between the actual value and the predicted value. Max Error can be used in two ways. Absolute Max Error, which is the largest absolute difference between actual and predicted values, and Relative Max Error, which expresses the largest error as a percentage of the data range. If a model is perfectly accurate, the Max Error will be zero, although this rarely happens in practice. The formula for Max Error is shown in Equation (40) [38].

(40) Max error=max(y−f(x))

Mean Absolute Error (MAE) calculates the absolute differences between actual and predicted values. The formula is shown in Equation (41) [38].

(41) $$MAE=\frac{1}{N}\sum_{i=1}^{N}\left|y_{i}-{\hat{y}}_{i}\right|$$

Mean Squared Error (MSE) calculates the squared differences between actual and predicted values. It gives greater weight to larger prediction errors, thus making the metric more sensitive to extreme errors. The MSE formula is shown in Equation (42) [38].

(42) $$MSE=\frac{1}{N}\sum_{i=1}^{N}{\left(y_{i}-{\hat{y}}_{i}\right)}^{2}$$

#### 5.2.1. Huber Loss

The Huber loss function behaves like MSE for small errors and like MAE for large ones. The Huber loss function is shown in Equation (43) [57].

(43) Ly,fx=12|y−f(x)|2,&y−fx≤δδ(|y−f(x)|−12δ),& otherwise

#### 5.2.2. Entropy-Based Loss Functions

In addition to error-based loss functions such as MAE, MSE, and Huber loss, entropy-based measures are widely used in classification problems, especially in probabilistic models and deep learning systems. This family of measures is particularly relevant in medical AI because diagnostic models often output predicted probabilities that are interpreted as risk estimates rather than only as hard class labels [1,58,59].

The most fundamental concept is Shannon entropy, which quantifies the uncertainty of probability distribution. In diagnostic applications, high entropy corresponds to uncertain predictions, which may reflect borderline clinical cases, noisy inputs, or limited evidence for a clear decision. Closely related, cross-entropy loss is one of the most commonly used objective functions for training classification models. It measures the mismatch between the true class label and the predicted probability distribution and is widely used because it encourages probabilistically meaningful outputs and supports stable optimization in neural networks [1,58,59].

Another important information-theoretic quantity is Kullback–Leibler (KL) divergence, which measures the divergence between two probability distributions. In clinical AI studies, KL divergence is frequently used to quantify differences between distributions (for example, comparing training and test populations) and to support analysis of dataset shift and generalizability. Since KL divergence is closely related to cross-entropy, these measures are often discussed together as part of a unified information-theoretic framework. In this manuscript, KL divergence is described in detail in next section [60].

Overall, entropy-based losses complement conventional error-based loss functions by providing a natural way to quantify uncertainty and probabilistic mismatch, which are central considerations when developing and evaluating diagnostic prediction models [1,58,59].

#### 5.2.3. Kullback–Leibler Divergence

Kullback–Leibler divergence, also known as the relative entropy or the I-divergence parameter, is mainly used to measure the difference between two distributions. It can be used as an evaluation parameter for databases, and also as a statistical distance and a loss function [60,61]. Assume that *P* is the actual probability distribution, and *Q* is a model probability distribution. The following formula measures how much these two distributions are different relative to each other. This formula is applicable to discrete distributions [60].

(44) $$D_{KL}(P,Q)=\sum P\left(x\right)log\left(\frac{P\left(x\right)}{Q\left(x\right)}\right)$$

*P*(*x*)/*Q*(*x*) represents the probability of event *x* according to *P* relative to *Q*. When two distributions are identical, the output will be 0. As such, by minimizing this function, it can be used as the loss parameter for classifiers and neural networks. This can be achieved by comparing the labels in the dataset to the output of a classifier using this method. The Kullback–Leibler divergence formula can be extended to work with two-by-two confusion matrix parameters. The formula is as follows.

(45) $$D_{KL}=\frac{\left(TP+FN\right)\times \log\left(\frac{TP+FN}{TP+FP}\right)+(TN+FP)\times \log\left(\frac{TN+FP}{TN+FN}\right)}{N}$$

By following the variables in a confusion matrix, it can be determined that in this formula, (*TP* + *FN*) is the actual distribution of the TRUE class and, subsequently, (*TN* + *FP*) is the actual distribution of the FALSE class. As such, (*TP* + *FP*) is the output of the binary classifier for the TRUE class, and (*TN* + *FN*) will be the output of the classifier for the FALSE class. To use KL divergence for multi-class systems, the following formula can be used, which is not that different from the usual KL divergence formula.

(46) $$CD_{KL}=\sum_{i}P_{i}\times log\left(\frac{P_{i}}{Q_{i}}\right)$$

Consider *P_i_* as the actual number of items in class *i*, and *Q_i_* as the predicted number of items in class *i*. KL divergence is used as a regularizer factor of the latent space in variational autoencoders (VAEs). Tuning of the latent space is essential to the quality of VAE [62].

### 5.3. Predicted Correlation

Correlation and linear regression are used to quantify the relationship between two numerical variables. Correlation indicates the strength of the linear relationship between paired variables and expresses it as a correlation coefficient. If both variables *X* and *Y* are normally distributed, the Pearson correlation coefficient (*r*) is computed. If normality is not assumed for one or both variables, a rank-based correlation coefficient, such as Spearman’s rho (ρ), may be used instead. There is a hypothesis test of the correlation that assesses whether a linear relationship exists between the two variables in the population, returning *p* < 0.05 if the relationship is statistically significant. A 95% confidence interval for the correlation coefficient is also used to provide an estimate of the population correlation [63].

#### 5.3.1. Pearson Correlation

The Pearson correlation coefficient measures how changes in one variable result in a change in another. The Pearson correlation coefficient (*r*) between two variables, *X* and *Y*, is calculated according to Equation (47) [64].

(47) $$rXY=\frac{Cov(X,Y)}{\hat{\sigma X}\hat{\sigma Y}}$$

In this equation, *Cov*(*X*, *Y*) is the covariance between *X* and *Y*. $\hat{\sigma Y}$ is the standard deviation of variable *Y*, and $\hat{\sigma X}$ is the standard deviation of variable *X*. This measure ranges from −1 to 1. *r* = −1 means a perfect negative linear relationship, where an increase in one variable results in a decrease in the other in a perfectly linear manner. In contrast, *r* = 1 represents a perfect positive linear relationship. If *r* = 0, it means no linear relationship exists between the two variables. This coefficient is helpful in quantifying the degree to which two variables are linearly related [64].

#### 5.3.2. Spearman’s Rank Correlation

When an increase in one variable (*X*) is genuinely associated with an increase in another variable (*Y*), but the relationship is not strictly linear, so the Pearson correlation coefficient may not be the appropriate choice. For example, consider the relationship between the dose of an antihypertensive medication and the reduction in systolic blood pressure. When the dose is very low, even a small increase may lead to a noticeable improvement in blood pressure. However, as the dose becomes higher, further dose increases often produce progressively smaller additional reductions, and the response may begin to plateau. This type of monotonic but non-linear relationship is common in clinical and pharmacological data and may not be appropriately captured by Pearson correlation, making rank-based methods such as Spearman correlation more suitable. This kind of nonlinear relationship means that using the Pearson correlation could lead to misleading conclusions. In such cases, the Spearman rank correlation is a more suitable alternative, as it captures monotonic relationships regardless of linearity. The Spearman rank correlation formula is in Equation (48) [64,65].

(48) $$\rho =1-\frac{6\sum_{i=1}^{n}{\left(x_{i}-y_{i}\right)}^{2}}{n\left(n^{2}-1\right)}$$

This formula measures the strength and direction of the monotonic relationship between two ranked variables. In this formula, *n* is the number of paired observations. *x_i_* is the rank of the *i*-th observation in variable *X*. Also, *y_i_* is the rank of the *i*th observation in variable *Y*. (*x_i_* − *y_i_*)^2^ is the squared difference between the ranks of each pair. The coefficient ranges from –1 (perfect negative monotonic correlation) to +1 (perfect positive monotonic correlation). A value of 0 indicates no correlation [64].

#### 5.3.3. Kendall’s Tau Correlation

In addition to Pearson and Spearman correlation coefficients, Kendall’s tau (τ) is another widely used non-parametric measure of association that quantifies the strength and direction of a monotonic relationship between two variables. Unlike Pearson correlation, which assumes linearity and is sensitive to outliers, Kendall’s tau is based on concordant and discordant pairs of observations and is therefore more robust when the relationship is not linear or when the data contain ties. This makes Kendall’s tau particularly useful in clinical and biomedical settings where variables may be ordinal (e.g., disease severity grades, Likert-scale ratings, or staging systems) or where measurements may cluster into repeated values due to limited resolution of laboratory assays. In practice, Kendall’s tau can provide a more reliable assessment of association in small-to-moderate samples and in datasets where tied ranks are common, complementing Spearman’s rank correlation as an alternative rank-based measure [65]. In Equation (49) for the calculation of *τ*, C represents the number of matched pairs in the predicted and correct outcome, and D represents the unmatched ones.

(49) τ=(C−D)(C+D)

### 5.4. Balanced Accuracy

In the evaluation of a classification model, accuracy may sometimes appear better than its true performance. In binary classification, the class imbalance problem occurs when the training set contains an unequal number of samples from each class. This imbalance can cause the classifier to become biased towards the majority class. Applying such a classifier to a similarly imbalanced test set, may provide an overly optimistic estimate of accuracy. In an extreme scenario, the classifier might categorize all test samples to the larger class, thereby achieving an accuracy equal to the proportion of majority class labels in the test set. Several strategies exist to address this issue. Reducing the sample size in the majority class or increasing the sample size in the minority class and also adjusting the cost of misclassification errors are used to reduce classifier bias [66].

However, although these methods can, in certain cases, mitigate bias, they do not generally guarantee protection against optimistic estimates of accuracy. Balanced accuracy, defined as the average of the accuracy obtained in each class, can provide a suitable alternative. The balanced accuracy is defined as Equation (50) [66].

(50) $$Balanced\;Accuracy=\frac{TP}{P}+\frac{TN}{N}=\frac{Sensitivity+Specificity}{2}$$

This metric essentially calculates the average of the actual positive rate and the actual negative rate. In other words, the mean of sensitivity and specificity. If a classifier performs equally well across both classes, this measure aligns with standard accuracy, defined as the proportion of correct predictions out of all predictions made. However, in cases where standard accuracy is artificially high due to class imbalance, such as when the model consistently predicts the majority class, the balanced accuracy decreases to the level of random chance. One of the strengths of balanced accuracy is that it treats both positive and negative classes equally, offering a symmetric evaluation. This assumption of symmetry can be relaxed if needed, in which case the formula in Equation (51) is adjusted accordingly [66].

(51) $$c\;\times \;\frac{TP}{P}+\left(1-c\right)\times \frac{TN}{N},where\;c\in [0,1]$$

where c ∈ [0, 1] represents the cost associated with the misclassification of a positive example. In simpler terms, when c is closer to 1, more importance is given to correctly identifying positive samples (as the cost of false positives is higher). Conversely, when c is closer to 0, more emphasis is placed on correctly identifying negative samples [66].

### 5.5. Jaccard Index

The Jaccard Index, also known as the Jaccard similarity coefficient, is one of the most widely used similarity measures for comparing two sets. It is especially appropriate when the objective is to quantify the overlap between two collections of elements. In clinical and medical research, the Jaccard Index is frequently used to compare binary patient representations, such as the presence or absence of symptoms, diagnoses (e.g., ICD codes), prescribed medications, genetic markers, or extracted clinical concepts from electronic health records (EHRs). The Jaccard Index provides an intuitive interpretation because it directly measures the proportion of shared elements relative to the total number of distinct elements across both sets.

Formally, given two sets *A* and *B*, the Jaccard Index is defined as the ratio between the size of their intersection and the size of their union in Equation (52).

(52) $$J(A,B)=\frac{|A\cap B|}{|A\cup B|}$$

The Jaccard Index ranges from 0 to 1. A value of 0 indicates that the two sets share no common elements, whereas a value of 1 indicates that the sets are identical. Compared to other binary similarity measures, the Jaccard Index is particularly useful because it ignores negative matches (i.e., features absent in both sets). This property is beneficial in medical data, where the number of absent clinical events is often much larger than the number of present events, and counting co-absences would artificially inflate similarity [67].

### 5.6. Weighted Discrete Elements

In many real-world medical applications, clinical features do not contribute equally to similarity. Some events, such as rare diagnoses or critical symptoms, may carry more importance than common ones. To account for this, the Jaccard Index can be extended into a weighted Jaccard Index, where each element is assigned a weight representing its clinical relevance, frequency, or importance. This variation is particularly useful in clinical decision support systems, patient similarity networks, and cohort retrieval tasks, where certain diagnoses or laboratory abnormalities should influence similarity more strongly than routine or less informative findings.

Let *w*(*x*) denote the weight associated with an element x. The weighted Jaccard Index can be defined as Equation (53).

(53) $$J_{w}\left(A,B\right)=\frac{\sum_{x\in A\cap B}w\left(x\right)}{\sum_{x\in A\cup B}w\left(x\right)}$$

This weighted formulation preserves the same conceptual meaning as the standard Jaccard Index but incorporates the magnitude of importance of shared elements. For example, in clinical phenotyping, two patients sharing a rare but severe condition (such as pulmonary embolism) might be considered more similar than two patients sharing only common conditions such as hypertension. Thus, weighting makes the similarity measure more clinically meaningful and aligned with medical priorities [68].

### 5.7. Addition-Based Multiset Jaccard Index

In several clinical contexts, patient data cannot be represented simply as sets, because features may appear multiple times. For instance, a patient may receive repeated medications, multiple hospital admissions, repeated lab tests, or recurring symptoms. In such cases, the data are better represented as a multiset, where elements have multiplicities (counts). The classical Jaccard Index does not capture this repetition, since it only considers whether an element is present or absent. To overcome this limitation, the multiset Jaccard Index (also called the generalized Jaccard similarity) can be used.

If *m_A_*(*x*) and *m_B_*(*x*) represent the multiplicity (count) of element *x* in multisets *A* and *B*, the multiset Jaccard Index is defined as Equation (54).

(54) $$J_{m}\left(A,B\right)=\frac{\sum_{x}min(m_{A}(x),m_{B}(x))}{\sum_{x}max(m_{A}(x),m_{B}(x))}$$

This formulation extends the Jaccard Index by measuring overlap based on shared counts. It is particularly useful in longitudinal EHR analysis, where the frequency of medical events carries clinical meaning. For example, two patients who both have diabetes may still differ significantly if one has repeated hospitalizations and intensive medication adjustments, while the other has only a single diagnosis record. The multiset Jaccard Index can better capture this difference by accounting for the number of occurrences of each event, making it well-suited for modeling chronic disease trajectories, medication adherence patterns, or repeated laboratory abnormalities [68].

### 5.8. Continuous Sets

In modern clinical machine learning, data are often represented in continuous form rather than binary form. Examples include normalized laboratory values, continuous vital signs, gene expression levels, imaging-derived biomarkers, or embedding-based feature representations. In such cases, the original Jaccard Index cannot be applied directly because the features are no longer discrete elements. However, the concept of intersection and union can be generalized to continuous vectors using minimum and maximum operators. This extension is often referred to as continuous Jaccard similarity.

Given two non-negative continuous vectors $a=(a_{1},a_{2},\ldots ,a_{n})$ and $b=(b_{1},b_{2},\ldots ,b_{n})$, the continuous Jaccard Index is defined as Equation (55).

(55) $$J_{c}\left(a,b\right)=\frac{\sum_{i=1}^{n}min(a_{i},b_{i})}{\sum_{i=1}^{n}max(a_{i},b_{i})}$$

This version maintains the same intuition as the classical Jaccard Index, but instead of counting shared elements, it measures overlap in terms of shared magnitudes. In clinical applications, this is useful for comparing patients based on continuous laboratory profiles or physiological measurements. For instance, two patients may have similar inflammatory profiles if their biomarkers overlap in magnitude, even if they are not identical. The continuous Jaccard similarity can therefore provide a robust similarity measure for patient stratification, disease subtyping, and monitoring progression in chronic conditions [68].

### 5.9. Comparison and Practical Differences Across Categories

Although all these measures fall under the Jaccard family, they differ in how they interpret “overlap” and what type of clinical information they are best suited for. The standard Jaccard Index is most appropriate when the data are purely binary, such as whether a diagnosis code or symptom exists in a patient record. The weighted Jaccard Index extends this setting by incorporating clinical importance, making it useful when some conditions or clinical concepts are more informative than others. In contrast, the multiset Jaccard Index is designed for event-based clinical data where repetition matters, such as multiple hospital admissions, repeated medication prescriptions, or frequent abnormal lab test results. Finally, the continuous Jaccard Index generalizes the same overlap idea to continuous-valued biomedical measurements, enabling similarity comparisons across lab results, physiological signals, or continuous patient representations.

In medical and clinical research, choosing the correct Jaccard variant depends on the nature of the data and the objective of the analysis. If the goal is to compare patients based on presence/absence of diseases, the classical Jaccard Index is often sufficient and interpretable. If clinical significance or rarity must be emphasized, the weighted version provides a more meaningful similarity score. If the temporal repetition or intensity of clinical events matters, such as distinguishing stable versus frequently hospitalized patients, the multiset formulation becomes more suitable. Finally, for continuous biomedical variables and modern machine learning representations, continuous Jaccard similarity offers a mathematically consistent and clinically relevant approach [68].

### 5.10. Matthews Correlation Coefficient (MCC)

When evaluating binary classification tasks and their corresponding confusion matrices, researchers can utilize various statistical metrics depending on the specific goal of the experiment, but there is still no universally agreed-upon standard for model evaluation. Accuracy and F1 Score are among the most widely used and recognized metrics, both derived from the confusion matrix. These measures remain popular in practice. However, in the context of imbalanced datasets, they can often provide an overly optimistic assessment of a model’s performance [69].

The MCC is an effective solution to address this issue. MCC is a robust metric for evaluating classification models, especially when the dataset is imbalanced. It provides a balanced measure by considering all four categories of the confusion matrix: true positives, true negatives, false positives, and false negatives. The MCC value ranges from −1 to +1. A value of −1 denotes a completely erroneous prediction, where all positive samples are classified as negative and all negative samples as positive. A value of +1 denotes a perfect prediction with no misclassifications, and value near 0 implies random guessing [38].

In some cases, where two values from the confusion matrix are simultaneously equal to zero, the MCC may be undefined. MCC is symmetrical with respect to class labels, meaning that switching the roles of the positive and negative classes will not affect the result. A key advantage of this metric is that it remains accurate even in cases where the dataset is imbalanced. In other words, an MCC value of zero does not necessarily imply a prediction accuracy of 1/N for an N-class system because the class distribution may not be uniform. The MCC is a discrete version of the Pearson Correlation Coefficient, and it is calculated using Equation (56) [38,69].

(56) $$MCC=\frac{TP\times TN-FP\times FN}{\sqrt{\left(TP+FN)(TP+FP)(TN+FP)(TN+FN\right)}}$$

In this formula, *TP* is the true positive numbers, *TN* is the true negative numbers, *FP* is the false positive numbers, and *FN* is the false negative numbers. The MCC is well-suited for imbalanced datasets. However, it cannot be easily generalized to multi-class classification problems. It is important to note that this equation can sometimes be an indeterminate form of 0/0. For instance, if *FN* + *TP* = 0, the model has predicted all samples as the negative class. In this case, since no predictions are made for the positive class, it often points to a fundamental issue with the model [38].

### 5.11. Hinge Loss

Loss functions depict the amount of cost one is willing to pay to use a predicted result instead of the actual outcome. When working with machine learning algorithms, there are many loss functions from which one can choose. Making the correct choice depends on the type of problem and the available dataset. It is generally considered an empirical issue [70].

Hinge loss is considered a linear loss function. It is also a convex function, which is important for optimization purposes, and while it is not differentiable, it has a subgradient. Typically, the Hinge loss function is used in training classifiers based on the support vector machine algorithm.

As shown in the work of Crammer and Singer, the Hinge loss function can also be extended to work with multiclass classifiers. It is also known as the Crammer-Singer method [71]. A basic Hinge loss function follows Equation (57).

(57) $$l\left(t,y\right)=max\;(0,1-ty)$$

**Figure 10:**
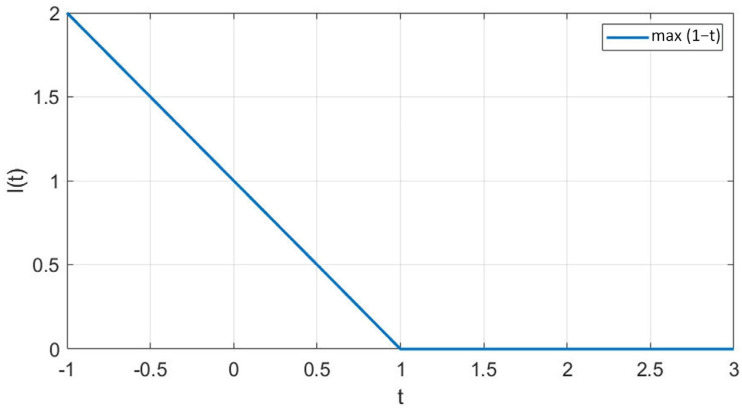
Hinge loss function with y being constant.

Parameter *y* is the label or the intended outcome and can either be +1 or −1, and *t* is the raw output of a binary classifier. As such, if the classifier predicts the item with sufficient margin (both *t* and *y* have the same sign), the output of the loss function will be 0. Otherwise, the loss increases linearly. This behavior can be seen in Figure 10.

In a study by Ozyildirim et al. [72], the Levenberg–Marquardt (LM) optimization function was implemented using the Hinge loss function as the cost factor to achieve faster convergence. In this study, the multiclass variation in the Hinge loss function was smoothed and used with the L2-regularization parameter and was then compared with a cross-entropy loss function.

As a result, a combination of LM and Hinge loss in multiclass classification problems converges to an optimal solution before the stochastic gradient descent optimization method and provides similar results [72].

### 5.12. Cohen’s Kappa

In the medical science field, a dataset might be collected and labeled by multiple people. As such, they might have different degrees of agreement on the labels assigned to the items present in the dataset. Such a phenomenon in data science is called inter-rater agreement or inter-rater reliability and can cause inter-rater related errors. Intra-rated reliability issues can occur when the person responsible for labeling a phenomenon repeatedly gets presented with the same data. The issue is, will they label that set the same way or not [73].

Cohen’s kappa is one of the most common methods among the tests used for measuring inter- and intra-rater reliability. This test is used explicitly for two raters. Fleiss’ kappa is the extended version of Cohen’s kappa that can work for three or more raters [73]. This test can also be used to evaluate binary classification models based on their two-by-two confusion matrix [74].

Cohen’s kappa can take a value ranging from negative one to one, where zero indicates agreement by random chance, and one shows a perfect agreement between the two raters. This parameter can also take negative values, which Cohen has specified is unlikely to happen in practice. Cohen’s kappa can be determined by using the following formula [73].

(58) $$ĸ=\frac{p_{o}-p_{e}}{1-p_{e}}$$

Here, *p_o_* represents the observed agreement between the two raters, and *p_e_* represents the probability of chance agreement. Since Cohen’s kappa is based on a chi-square table, *p_e_* can be calculated by the formula shown below [73].

(59) pe=cm1 × rm1n+cm2 × rm2nn

where *cm*_1_ represents column 1 marginal or sum, *cm*_2_ is column 2 marginal or sum, *rm*_1_ shows row 1 marginal or sum, *rm*_2_ is row 2 marginal or sum, and *n* is the number of observations [73]. Table 8 shows how to interpret Cohen’s kappa for inter and intra-rater reliability problems.

As shown by D. Chicco et al. [74], Cohen’s kappa can also be used to measure the performance of a binary classifier, similar to how MCC is used. The formula for Cohen’s kappa can be rewritten in terms of confusion matrix variables and is as follows.

(60) $$ĸ=\frac{2\times (TP\times TN-FP\times FN)}{\left(TP+FP\right)\times \left(FP+TN\right)+(TP+FN)\times (FN+TN)}$$

where *TP* is true positives, *TN* is true negatives, *FP* is false positives and *FN* is false negatives. Here, a value of zero indicates that the predictions were similar to random guesses, a value of one being a perfect classification, and negative one indicates perfectly wrong classification [74].

In a study done by Vieira et al. [75], Cohen’s kappa was used as a performance metric alongside accuracy in a feature selection algorithm on five datasets in the UCI machine learning repository. In general, Cohen’s kappa had slightly better subset solutions while using fewer features, and no significant change in standard deviation was detected.

## 6. Cross-Validation

A well-known method used in machine learning (ML) for evaluating models and estimating prediction errors is called cross-validation (CV) [13]. Cross-validation is a useful technique that resamples data into several training and test sets. This approach offers several advantages. It provides a constant way to evaluate models, helps in reducing overfitting, supports model’s hyperparameter tuning, makes more effective use of limited data, and assists in estimating the generalization error of a predictive model [76]. Even with its benefits, cross-validation is not without its downsides. It can be time-consuming and computationally intensive, especially when working with large datasets or complex models, which can slow down the training process. Additionally, it is not the best choice for certain data types, like time-series data, where the data points are connected in a sequence and not independent. Various cross-validation techniques are designed to address diverse situations. Examples of these include K-fold cross-validation, stratified cross-validation, Monte Carlo cross-validation, and Hold-out cross-validation. These methods work by dividing the data into multiple smaller folds or subsets. Therefore, ensure a fairness evaluation process; in other words, every piece of data has an equal chance of being assigned to the training and test sets. A comparison of these cross-validation methods is provided in Table 9.

### 6.1. Hold-Out Cross-Validation

Hold-out cross-validation is a simple method where the dataset is split into two or three sets, typically training and test set (and sometimes validation), with no repetition of the split [76]. The model is trained on the training set and evaluated on the test set. This approach is commonly used for working with large datasets because it allows for quick evaluations. The main benefits are an easy-to-use method, speed, and efficiency, particularly when a small sample of the data can effectively represent the whole dataset. However, it has significant drawbacks. It may waste valuable data in the small dataset, and without a stratified approach, it might fail to preserve class distribution in imbalanced datasets. Unlike K-fold and Leave-one-out cross-validation, Hold-out does not give a robust performance estimate.

### 6.2. K-Fold Cross-Validation

**Figure 11:**
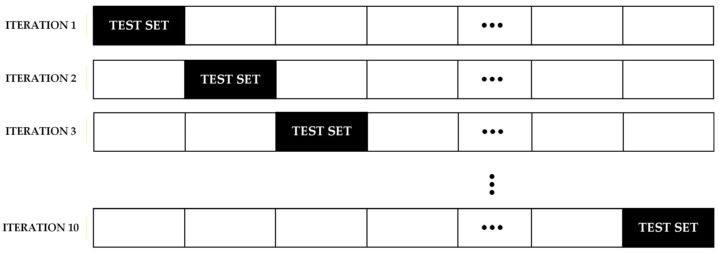
Ten-fold cross-validation; the dataset is divided into 10 parts, where each black block represents the test set in one iteration and the white blocks represent the training set.

The most commonly used method for evaluating models with cross-validation is K-fold cross-validation. K-fold cross-validation splits the dataset into k folds of equal size, then uses one of these folds as a test set and the remaining folds as a training set. This process is repeated k times, and each time a different fold is used as a test set. The final performance is calculated as the average of all k-test results [13]. K-fold cross-validation is a technique used when a more robust estimate of model performance is needed, especially when working with small to medium-sized datasets or during hyperparameter tuning. It ensures that each data point is used as part of both training and testing sets, which helps reduce bias compared to the Hold-out method [76]. The benefits of K-Fold cross-validation include efficient use of data, a better estimate of model generalization, and reduced variance compared to Hold-out validation. However, it also has drawbacks such as increased computational cost (as the model is trained k times), and choosing k that is too small or too large may result in higher variance or longer runtimes, respectively. When it comes to a classification task, we often use a method called Stratified K-Fold. This method makes sure that the different categories or classes are evenly distributed when the data is split into parts or sections. It keeps the overall balance of the data in each part, making the analysis or model training more accurate and reliable. This process is visually demonstrated in Figure 11.

### 6.3. Leave-One-Out Cross-Validation (LOOCV)

**Figure 12:**
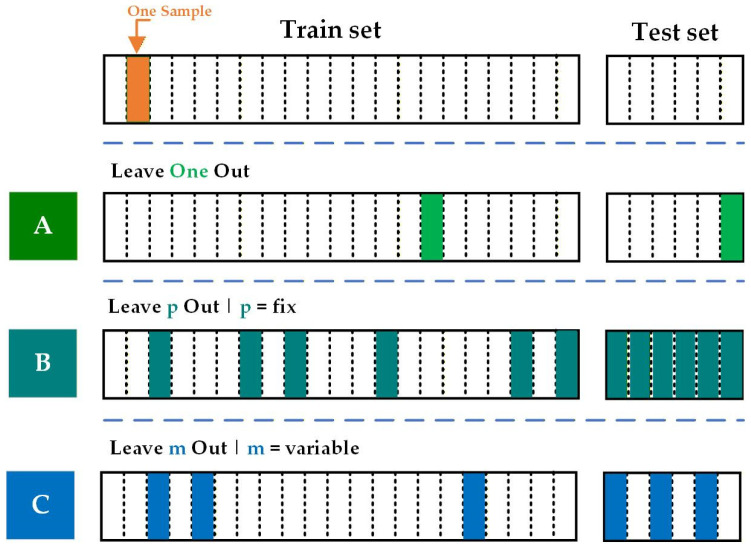
(**A**) Leave-One-Out uses one sample for testing and the rest for training, repeating for every data point, (**B**) Leave-p-Out, and (**C**) Leave-m-Out use fixed and variable numbers of samples for testing, respectively, with the remaining data forming the training set.

Leave-one-out cross-validation is a cross-validation method that removes a single data point and the model trains on the remaining data points; then, the excluded data point in the dataset is used as a test set to assess model performance. This procedure is repeated for every data point in the dataset, and then for the result, we average the performance across all runs. It is applicable when the dataset is small and we want to make something out of the data and train a robust model. The most commonly used are in medical, biological, or experimental studies where every point is valuable, and the benefits are that every sample is used, and we do not use a random split, and provides a nearly unbiased estimate of generalized error; it also slows training due to being computationally expensive [76]. We illustrate this method in Figure 12.

### 6.4. Leave-P-Out Cross-Validation (LPOCV)

Leave-P-Out Cross Validation (LPOCV) is a technique where, in every round, p samples are taken out from the dataset for testing purposes. The model is trained with the remaining samples (leave-m-out), similar to Leave-One-Out Cross-Validation (LOOCV), but more than one sample is left out here. The process is repeated for every possible group of p samples in the dataset, which makes LPOCV a very comprehensive method to assess how well the model performs. Numerous combinations are used in this method, which ensures a thorough check of the model’s effectiveness [76].

Leave-P-Out Cross-Validation (LPOCV) is an approach used for evaluating the model when highly accurate results are required. When working with small datasets, every piece of data matters a lot; this technique is highly efficient. Such cases frequently occur in fields like medical or experimental research, where precision is crucial. LPOCV is advantageous because it reduces bias by considering all possible ways to split the data into training and test sets. This comprehensive approach makes it a reliable tool for predicting how well a model will perform. However, one significant downside is the high amount of computer power it requires [76]. This is particularly relevant when the value of p gets larger or when working with big datasets. The number of combinations it needs to calculate increases dramatically, making it less practical for medium to large datasets or for models that take a long time to train. We illustrate this method in Figure 12.

### 6.5. Monte Carlo Cross-Validation

Monte Carlo Cross-Validation or Repeated Random Sampling Validation is a technique to check how well a model works by repeatedly splitting the data into training and testing parts. In each run, a model is trained on the randomly selected training set and evaluated on the corresponding test set, and the final performance is averaged over all runs [76]. In Monte Carlo cross-validation, the way data are split can overlap, unlike k-fold cross-validation. Therefore, not every data point is used in each iteration. Monte Carlo cross-validation is effective when working with a large dataset or when evaluating a model’s performance with different random data splits. It offers flexibility by allowing the user to define the sizes of the training and test sets. It is also easy to use and helps understand how much the model’s performance can change. However, there are some downsides. In different iterations, the same data might appear in both the training and test sets. Certain data samples may be used more often than others, and without setting a fixed random seed, the results cannot be easily repeated. Monte Carlo cross-validation is typically used to test the stability of a model, especially when other methods like k-fold or leave-p-out require too much computing power.

### 6.6. Time-Series Cross-Validation

**Figure 13:**
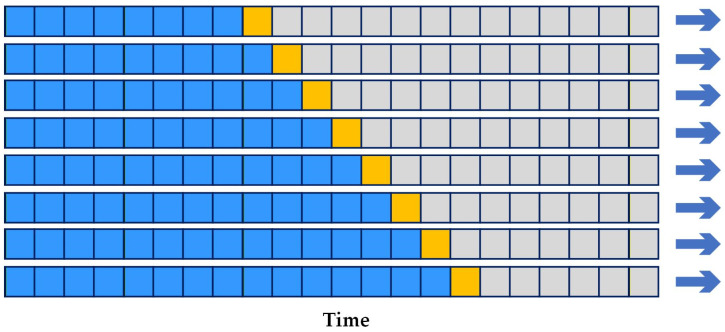
Time-series cross-validation. The data are split in time order; in each step, earlier blue points are used to train, and the next orange point is used to test. The arrows indicate the forward progression in time with each split.

Time-series cross-validation is a special method used when the order of events in data matters. This is often seen in studies that track patients over time, like long-term medical research. Unlike regular cross-validation, which does not keep track of the order of events, time-series cross-validation makes sure not to use future information to guess earlier events. One standard method for this is forward chaining, also called rolling-origin [77]. In this approach, the model learns from earlier data and tests on later data, for instance, in a patient’s continuous glucose monitoring data collected over several months. The model can learn from the first two months of data to predict what happens in the third month. Then, it trains in the first three months to forecast the fourth month, continuing this way each time. This ensures that the predictions respect the timeline of the data. This approach is very fundamental in ensuring that the analysis accurately forecasts future trends based on past data. This method is visually demonstrated in Figure 13.

This method helps predict healthcare needs, model disease progression, and predict hospital readmission risks. In these fields, it is crucial to use only past data to predict the future, similar to how we make decisions in real life. The main benefit is the ability to make accurate predictions without revealing additional information, especially in time-sensitive situations. However, there are some downsides. As the training set becomes larger with each data split, it demands more computer power [77]. In the beginning, there is less training data available, which can be a challenge. Another problem is dealing with changes over time, like shifts in patients’ behavior or variations in treatment over time.

### 6.7. Stratification Cross-Validation

**Figure 14:**
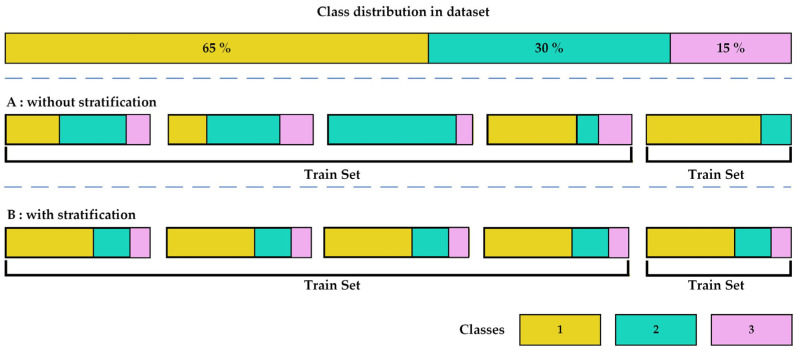
Stratified vs. Non-Stratified Cross-Validation, (**A**) without stratification, some folds may miss certain classes, making them unbalanced; (**B**) with stratification, each fold keeps the same class distribution as the whole dataset, making all folds more representative.

Stratified cross-validation is a method used in data analysis, especially for classification problems with uneven class distribution. This method partitions the dataset into smaller parts called folds. Each fold keeps the same distribution of class labels as the entire dataset [76]. This is very useful when the classes have unequal representation, such as in medical diagnosis. There may be more healthy individuals than those with a disease. As an example, in a cancer detection dataset where 10% of the samples show cancer and 90% are healthy, stratified cross-validation ensures this 10% to 90% ratio is maintained in both training and test sets. Each fold will represent the whole dataset’s proportions, which enhances the reliability and accuracy of the analysis for predicting outcomes. It plays the key role, especially when dealing with significant class imbalances commonly seen in real-world issues like healthcare diagnostics. Figure 14 shows how stratification impacts the resampling process.

In medical tasks, such as predicting if a person has a disease, assessing the risk of a patient returning to the hospital, or determining how well a treatment might work. It is important because it improves the model by balancing the different groups or classes that we are examining. Stratified cross-validation has some great benefits; it helps make the model evaluation fairer, decreases bias, and enhances the understanding of new data, particularly when the data are unbalanced. However, there are some challenges, too [14]. Implementing it can be complex, especially for tasks involving multiple labels or in regression scenarios [13]. It is also less adaptable with tiny datasets because it is challenging to maintain the right class balance in every data segment.

### 6.8. Grouped Cross-Validation

**Figure 15:**
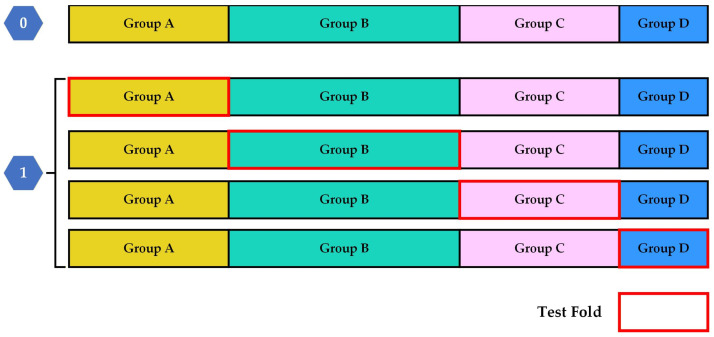
Grouped Cross-Validation, (**0**) the dataset has four separate groups; (**1**) in each step, three groups are used to train, and 1 group is used to test, and this is repeated for all group combinations.

Grouped Cross-Validation (or Block Cross-Validation) is a technique used when handling data. In this approach, data samples are organized into groups based on a specific identifier, such as patient ID, device ID, or session ID. When dividing the data, all samples from one group are kept together, either entirely in the training set or entirely in the validation set [76]. This prevents mixing, ensuring samples from the same group do not end up in both sets. This technique is important because it stops data leakage, which can happen if related samples from the same group are split between training and test sets. For instance, in a medical imaging study, if several images come from the same patient, grouped cross-validation makes sure all those images stay together in one set. This technique is illustrated in Figure 15.

This approach is especially useful in clinical research, EEG/ECG signal analysis, medical imaging, or wearable device data, where data points from the same subject or recording session are not independent. The main benefit of using this approach is that it gives a more accurate idea of how well a model will perform in real situations where data are grouped or linked. Additionally, it helps to prevent overfitting by minimizing the risk of data leaking between groups. However, its drawbacks include fewer unique splits, which can reduce evaluation variability, and uneven fold sizes if groups vary significantly in sample count.

## 7. Computational Complexity

Computational complexity has been discussed for a long time, and how much computing power a model will require to make predictions or train on. This is relevant because even a brilliant model is useless if it takes ages to implement or requires a ton of computing power. Computational complexity enables us to discuss whether a model is computationally feasible in actual implementations, especially when we need to make a choice under a time constraint or when we do not have much hardware. By looking at factors like memory and processing time, we are able to achieve a balance between usability and performance so that the model, aside from being good, is also feasible to deploy and sustain in use.

### 7.1. Classification Latency

In machine learning models, the time it takes for an input to be received and converted into an output is called classification latency. In fact, classification latency is the time required to preprocess the input (prepare the data), compute the model (perform mathematical operations on the model layers), and generate the output (convert the model results into a usable prediction).

Many factors affect classification latency, including model complexity (such as complex neural networks requiring higher latency), computational resources (processor (CPU) speed, graphics processing unit (GPU) or other hardware), input size (larger data requires more processing time), model optimization (techniques such as quantization can reduce latency), and execution environment (latency in cloud environments may be higher due to network connections).

Classification latency is significant in real-time applications such as ICU alarm prediction, emergency triage decision support, radiology workflow prioritization, because low classification latency is crucial for delivering seamless performance and maintaining a high-quality user experience. This metric is very useful in performance evaluation and is often evaluated against rigorous operational benchmarks to ensure that the system meets real-world needs [78].

(61) $$\frac{1}{N}\sum_{i=1}^{N}(T_{mr}-T_{di})$$

In Equation (61), the total number of data is represented by *N*, the time required for machine learning to infer the *i*th sample is represented by *T_mr_*, and the time required for the *i*th sample to enter the inference stage is represented by *T_di_*. Therefore, classification latency has a significant impact on real-time applications, and high latency can cause poor performance of real-time systems [78].

### 7.2. Classification Throughput

Classification throughput is the number of inferences that a machine learning model performs per unit of time. Classification throughput is the rate at which a classification system, such as a machine learning model, processes and classifies input data samples within a specified time interval and is expressed in units of samples per second. It serves as a critical performance metric for evaluating the efficiency and scalability of classification algorithms, especially in scenarios that require fast processing of large volumes of data. This metric is expressed in samples per second [16].

(62) $$\frac{N_{cla}}{T_{e}-T_{b}}$$

In Equation (62), *N_cla_* is the number of data or samples (such as images, texts, or records) that the classification system processes and classifies in a given time, *T_e_* is the time (in seconds) that the inference takes to complete for *N_cla_* samples, and *T_b_* is the time (in seconds) that the inference starts for *N_cla_* samples.

Classification throughput is essential in areas such as batch radiology screening, pathology slide processing, ECG stream processing, and fraud detection systems, where fast and accurate classification of large data sets is critical for operational success.

Various factors can affect Classification throughput, as this metric is highly dependent on system performance and environmental conditions. Factors include model optimization (model architecture, complexity reduction, and batch processing), hardware (processors, memory, and scalability), and data type (data volume and complexity, data preprocessing, and data variety). Classification throughput is improved by balancing model accuracy and processing speed. The most important steps to achieve higher classification throughput include model-level optimization, selecting the proper hardware, and managing the type and volume of data [16,78,79].

### 7.3. Classification Efficiency

Classification efficiency evaluates the performance of a classification system (such as a machine learning model) by considering a combination of accuracy and computational resources consumed (such as time, energy, or memory). Classification efficiency is used to measure the performance of models in environments such as real-time systems. Classification throughput only measures processing speed, while efficiency, on the other hand, considers the overall optimization of the system with respect to output quality and computational costs. Classification efficiency indicates the accuracy of a machine learning model in classifying data while optimizing computational resources, and its goal is to achieve speed and low computational cost in addition to accuracy.

Various factors, such as model accuracy, model optimization, hardware, and data type, affect Classification efficiency. More accurate models have higher efficiency, but if they consume a lot of resources, efficiency may decrease. In model optimization, techniques such as quantization, pruning, or using lightweight architectures can reduce resource consumption and increase efficiency. Optimized processors also reduce power consumption and time, and more complex data (such as high-resolution images) consume more resources and may reduce performance.

There is no standard metric for calculating classification efficiency; it is defined in different ways depending on the application and is a function of accuracy characteristics [78]. Classification efficiency is a multidimensional metric. Its calculation depends on the specific project objective and criteria, and the selection of the appropriate formula should be made according to the intended application [80].

### 7.4. Energy Consumption

Energy consumption refers to the amount of electrical or computational energy consumed by a system during the execution of a specific task, such as data classification. Energy consumption is used in contexts such as resource-constrained devices to assess energy efficiency or machine learning and performance optimization. Energy consumption in the field of classification indicates the amount of energy required to process data (text, signals, or images) by the model and is often investigated with the aim of reducing computational costs and environmental impact.

Energy consumption is reported in joules (J), watt-hours (Wh), or millijoules (mJ) for each sample or the entire process, and in some cases, power in watts (W) is also used for instantaneous analysis. Since energy consumption represents the energy required to power the system, it can be calculated by measuring the total power for some time [78].

(63) $$Energy\;consumption=\frac{P}{T}$$

Average power can also be calculated as energy measured in joules over a given time interval:

(64) $$P=\frac{E}{T}$$

By combining Equations (63) and (64) above, we can calculate the system performance per watt:

(65) $$\frac{\frac{P}{T}}{\frac{E}{T}}=\frac{P}{E}$$

Artificial intelligence systems, especially advanced models such as deep learning models, require substantial computational resources for execution and training, which is due to model training, execution, data centers, and data storage and transmission. Therefore, high energy consumption is due to the need for heavy processing, data centers, and cooling, which increases electricity, infrastructure, and maintenance costs. To reduce the costs caused by high energy consumption, optimized algorithms, optimized hardware, and renewable energy can be used [81].

## 8. Statistical Tests of Significance

Statistical significance tests are used to determine whether the model’s result is statistically significant. They help determine if differences in a model’s performance or correlations are significant enough to be tolerable. Researchers can draw evidence-based conclusions about the validity and accuracy of their findings with the help of these tests. This is especially important in scientific and medical environments, where decisions based on inconclusive or random results can prove catastrophic. Generally, statistical significance testing adds another level of gravity and conviction to the evaluation process.

### 8.1. Paired Student’s t-Test

To evaluate whether the differences between two related groups are statistically significant under a normal distribution, researchers often apply the paired Student’s *t*-test, which compares their means and standard deviations. However, this method is unsuitable for analyzing more than two groups. Often used in assessing whether differences in model accuracy are statistically significantly different in machine learning, it should not be used along with k-fold cross-validation, as the repeated data resampling does not satisfy the independent observations assumption. A better alternative is Dietterich’s 5 × 2 cross-validation approach [82], which alleviates dependency by conducting five instances of two-fold cross-validation. In each instance, the data are split into training and testing sets, both configurations are evaluated, and the performance metrics are calculated [78].

In this context, each of the *n* pairs of observations is considered independent. The paired observations are labeled by $x_{i}$ and $y_{i}$, and their difference by *d_i_* for *i* = 1, …, *n*. Consequently, the random variable *D* = *X* − *Y* is also normally distributed, with an expected value of $\mu_{d}$ = $\mu_{x}$ − $\mu_{y}$ and variance $\sigma_{d}^{2}$. The hypothesis under investigation is the null hypothesis (H_0_: $\mu_{d}$ = 0), which posits that no systematic difference exists between the paired means. A Student’s t statistic is computed according to Equation (66).

(66) $$t=\frac{\bar{d}}{S_{d}/\sqrt{n}}$$

The test statistic is assessed in relation to Student’s t-distribution with n − 1 degrees of freedom. The procedure is similar to a randomized block analysis of variance, with paired measurements as the block variable. When the variances in the measures for X and Y are equal, the variance in the measures for D takes the form $2\sigma^{2}(1\;-\;\rho )$ as deriving from prior established theory. Even in negative correlations, capable of inflating variance, the scenario is hardly encountered in practice, due to careful pairing procedures. When variances are different, it leads to variances behaving differently. Statistically, in terms of efficacy, a paired *t*-test surpasses a two-sample independent *t*-test in designs parallel to each other. With the correlation coefficient nearly a third, relative efficiency is 1.5, so 100 paired observations are statistically as powerful as 150 independent ones per group [83].

Possible paired samples could include spouses, twins, the same student cohort from two classes, the same instructors examined in fall and spring, a stable set of professionals tested at multiple points throughout a career, a student cohort receiving a pretest and a posttest, or a sample of people being assessed for body mass index before and after exercise intervention. Consider the following example [84].

As presented in Table 10, the systolic blood pressure (SBP) of 20 patients was recorded at baseline, after 30 min, and as paired differences, yielding values of 79.40 ± 7.60, 84.60 ± 7.57, and 5.20 ± 2.49, respectively. The paired samples *t*-test indicated that the mean difference in paired measurements of the SBP obtained at 30 min and baseline after the intervention was statistically significant (*p* < 0.001) [85]. The paired-samples *t*-test is considered more robust than the one-sample *t*-test. Nonnormality has a limited impact, particularly when sample sizes exceed 30. However, outliers can affect results via a change in variance and skewness. Extreme data points, especially ones as extreme as *p* < 0.001, are less likely to be real when the data are not normal. Dependencies within paired observations, such as clustering, perturb the estimated variance, and this renders the significance values invalid. Heteroscedasticity and missing data, especially non-randomly missing data, are problematic [83].

### 8.2. Analysis of Variance (ANOVA)

The F-statistic, as used as the test statistic in Analysis of Variance or ANOVA, is a method used in comparing three or more groups’ means in a bid to find out if their average sizes are substantially different. ANOVA relies on a host of assumptions, among which is homogeneity in the variances in groups, and it can be tested through Levene’s test. The data are also typically assumed normally distributed, and the observations are assumed to be independent; therefore, they are assumed not to be related or correlated [86].

ANOVA estimates both the variability among the means of groups as well as within groups. The sum within every individual group, considering its mean, estimates the within-group variance. The sum of squared differences among individual groups and the overall mean estimates the amount by which the means among groups deviate from the grand mean. The ANOVA F-statistic is a ratio of intergroup mean squared variability proportions and intragroup mean squared variability proportions [78].

Types of ANOVA are determined by the number of factors or independent variables [86]. For instance, when there is a single categorical factor, the analysis is classified as one-way; when two categorical factors are present, it becomes a two-way ANOVA, and post hoc tests are then used to perform pairwise comparisons within groups [85].

Test statistics such as LSD, Bonferroni, or Tukey’s are used to make multiple comparisons publicly available under homogenous variances (*p* > 0.05). In the presence of non-homogeneous variances (*p* < 0.05), alternative post hoc tests such as Games–Howell and Tamhane’s T2 are recommended [87]. Table 11 provides an example of calculating the F-score and *p*-value.

The F-score is calculated using the formula presented in (67).

(67) $$F=\frac{\sum_{j=1}^{p}n_{j}{\left(x_{j}-x_{..}\right)}^{2}/\left(p-1\right)}{\sum_{j=1}^{p}\sum_{i=1}^{n}{\left(x_{ij}-x_{.j}\right)}^{2}/\sum_{j=1}^{p}(n_{j}-1)}$$

As shown in Equation (67), $x_{.j}$ denotes the mean value in the *j*th group, calculated as $\sum_{i=1}^{n}x_{ij}/n_{j}$ represents the overall mean value; p represents the number of groups; $x_{ij}$ denotes the *i*th observation in the *j*th group; and $n_{j}$ and n signify the number of observations in the *j*th group and the aggregate number of observations, respectively. For Table 11 dataset, the F equals six, and because it is larger than one, it indicates a difference among groups, or on the other hand, *p* < 0.05 [88,89]. ANOVA is traditionally applied in applied research in an effort to determine whether the conditions of the treatment, demographically different groups, or experimental conditions produce significantly different outcomes. In education, psychology, and medicine, it is a cornerstone technique [90].

Although the classical ANOVA F-test is widely used for comparing the means of three or more groups, it is strongly dependent on the assumption of homogeneity of variances across the groups. In many real-world medical and clinical datasets, this assumption is often violated due to differences in population characteristics, unequal sample sizes, or heterogeneity in patient responses. For instance, when comparing laboratory biomarker values across treatment groups, one group may contain more severely ill patients and therefore exhibit substantially larger variance than the others. In such cases, applying the standard ANOVA F-test may lead to misleading conclusions because the calculated F-statistic becomes sensitive to unequal variance, potentially inflating Type I error rates and producing incorrect statistical significance.

When variances are not equal, the traditional F-test is no longer fully reliable because it assumes that the pooled within-group variance is an accurate estimate of the true error variance. If one group has much higher variability than the others, the mean square within groups may not represent the same underlying distribution across groups. This is particularly problematic in clinical trials or observational studies where patient heterogeneity naturally produces unequal dispersion of outcomes. Therefore, before relying on the standard ANOVA F-test, variance homogeneity is often checked using tests such as Levene’s test or Brown–Forsythe test, and if the assumption is violated (e.g., *p* < 0.05), alternative robust approaches are recommended.

A widely accepted alternative is Welch’s ANOVA, which is an adaptation of the F-test designed specifically for comparing group means under heteroscedasticity (unequal variances). Welch’s ANOVA modifies the test statistic by weighting group means according to their variances and sample sizes, and it uses an adjusted approximation for the degrees of freedom. This makes the test more robust when the group variances differ substantially, particularly when sample sizes are unequal. In medical applications, Welch’s ANOVA is commonly used when comparing clinical measurements such as blood pressure, glucose levels, or imaging-derived biomarkers across multiple patient groups, since such variables often demonstrate different levels of variability between healthy individuals and patients with advanced disease.

In addition to Welch’s ANOVA, the Brown–Forsythe F-test is another robust option that can be applied under variance inequality. This test uses deviations from the median rather than the mean, which reduces sensitivity to extreme values and outliers. This property is especially valuable in clinical data analysis, where distributions are frequently skewed and may contain abnormal measurements due to acute conditions or measurement noise. Consequently, Brown–Forsythe-based methods are often preferred when the dataset contains outliers or when normality is questionable in addition to unequal variances [91].

### 8.3. Mann–Whitney U Test

The Mann–Whitney U test, also referred to as the Wilcoxon rank-sum test, is a widely used non-parametric statistical test designed to compare two independent groups. It is commonly applied as an alternative to the independent samples *t*-test when the assumptions of normality are not satisfied. In medical and clinical research, the Mann–Whitney test is frequently used when comparing outcomes such as laboratory values, symptom severity scores, pain scales, or physiological measurements between two patient groups (e.g., treatment vs. control), especially when the data are skewed or contain outliers.

Unlike parametric tests that compare group means, the Mann–Whitney test evaluates whether the distribution of values in one group tends to be systematically higher or lower than the other group. It achieves this by converting the raw observations into ranks across both groups combined and then analyzing the sum of ranks within each group. This makes the test robust to non-normal distributions and suitable for ordinal data, which is common in clinical questionnaires and diagnostic scoring systems.

Let two independent samples be represented as group 1 with size *n*_1_ and group 2 with size *n*_2_. After ranking all observations together, the Mann–Whitney U statistic for the first group is computed as Equation (68).

(68) $$U_{1}=n_{1}n_{2}+\frac{n_{1}(n_{1}+1)}{2}-R_{1}$$

where $R_{1}$ represents the sum of ranks of observations in group 1. Similarly, the statistic for the second group is as Equation (69).

(69) $$U_{2}=n_{1}n_{2}+\frac{n_{2}(n_{2}+1)}{2}-R_{2}$$

where $R_{2}$ is the sum of ranks in group 2. The final test statistic is typically taken as Equation (70).

(70) $$U=min(U_{1},U_{2})$$

A smaller U value indicates a larger difference between the groups. The *p*-value is then obtained by comparing the calculated U statistic to its sampling distribution, and for larger sample sizes, a normal approximation is often applied.

In summary, the Mann–Whitney U test is a powerful and flexible method for comparing two independent groups when the data are not normally distributed or when the measurement scale is ordinal. Due to its robustness and simplicity, it is widely used in clinical studies and biomedical research to assess whether two patient groups differ significantly in terms of a given outcome measure [92].

### 8.4. Kruskal–Wallis Test

An advanced non-parametric statistical analysis is the Kruskal–Wallis test, which is based on rank-based methods, essentially indicating whether there is a difference among three or more independent groups assumed to have been drawn from the same population or probability distribution. When the value of the test statistic obtained is larger than the value of the critical value, there exists evidence that the groups differ in distribution [78]. The Kruskal–Wallis test is a general extension of the Mann–Whitney U test, serving as the non-parametric counterpart to a one-way ANOVA [93]. The Kruskal–Wallis H statistic is determined according to the methodology outlined in (71).

(71) $$H=\frac{12}{N(N+1)}\sum_{i=1}^{k}\frac{R_{i}^{2}}{n_{i}}-3(N+1)$$

As shown in Equation (71), k is the number of groups, $R_{i}$ is the sum of ranks for group i (i = 1, 2, 3), and $n_{i}$ is the number of observations in group i. The normalized factor 12/N(N+1) is a normalizing coefficient. The test statistic of 5.96 is compared to the chi-square critical value with 2 degrees of freedom (k−1 = 2). The necessary values for this calculation, including group ranks and their sums, are summarized in Table 12. At a significance level of α = 0.05, the critical value is 5.99 [94,95]. Because 5.96 is less than 5.99, the null hypothesis is not rejected. Therefore, there is no statistically significant difference among the groups [96].

### 8.5. Chi-Squared Test

Chi-squared test compares whether observed and predicted frequencies in a set or sets of independent, categorical variable groups (the frequency data) differ. The chi-squared test is typically applied in questionnaires or experiments to determine whether distributions are statistically different. The chi-squared test represents a statistical technique applied in establishing association, specifically establishing a relationship between two variables, through quantitative processes. The test constitutes a statistical test designed for a specific end, namely, establishing whether there is an association between two variables. To test independence in a particular model, a chi-squared test is applied in the form of a contingency table. The chi-squared test is regarded as the superior technique in testing general significance in both observed and predicted frequencies [78].

The 2 × 2 chi-square test from a contingency table is applied in determining the specific question of whether the two variables are statistically associated with each other [94]. However, no measure of effect (risk ratio or odds ratio) exists [97].

The basic chi-squared statistic is computed as described in Equation (72), which outlines the method used in this analysis.

(72) $$\chi^{2}=\sum \frac{{\left(\mathrm{Observed}-Expected\right)}^{2}}{Expected},\;\mathrm{df}=1\;\mathrm{for}\;a\;2\times 2\;\mathrm{table}$$

When the data are intricate, the equation is adjusted in data arrangements as shown in Table 13. Roundings are made consistent prior to calculating Equation (73), which recasts the chi-squared measure in the new configuration [94].

(73) $$\chi^{2}=\frac{n{\left(d_{1}h_{0}-d_{0}h_{1}\right)}^{2}}{d\cdot h\cdot n_{1}\cdot n_{0}},\;\mathrm{df}=1$$

Using the information in Table 14, which presents the outcome of a trial to treat COVID-19, the chi-square statistic turned out to be 87.543. On one degree of freedom, the value is far larger than the critical value (10.83 at the 0.1% level) and thus represents p < 0.001. This result demonstrates that the likelihood of such a difference in size occurring by chance at random is very small and demonstrates a statistically significant association between COVID-19 outcome and treatment.

Chi-squared testing is most appropriate for testing categorical data summarized in contingency tables when the focus is on testing differences in distributions or testing for the existence of independence among groups. It comes in very handy in clinical studies to compare arms in the treatment because the test offers an indication of whether or not frequencies observed for outcomes significantly deviate from what would be anticipated under the null hypothesis of homogeneity. By applying a straightforward interpretation of the chi-squared measure, investigators can determine if categorical predictors such as exposure status or treatment condition are reliably related to outcomes [98].

### 8.6. Wilcoxon Signed-Rank Test

A non-parametric alternative to the paired Student’s *t*-test for ranked data is the Wilcoxon signed-rank test [99]. This test compares rankings of positive and negative differences and evaluates the performance disparities between two classifiers for each dataset. The goal is to determine whether any randomly selected observation will either exceed or fall short of a sample from the distribution of the other dataset. To identify clusters at opposite ends, which would indicate that the results do not meet the significance test, the ranked values for the two datasets are interleaved [78]. The null hypothesis in the Wilcoxon signed-rank test states that the distribution of pairwise differences is centered at zero [100].

This is the non-parametric equivalent of the paired *t*-test, and it checks whether the median of the paired differences in the population from which the sample is drawn is zero. All differences equal to zero must be excluded from the analysis. The remaining differences are then considered based on their absolute values, disregarding their signs. These absolute differences should be arranged in ascending order of magnitude and assigned ranks starting from one. In instances where two or more differences share the same magnitude, the ranks they would have received are averaged, and that average rank is assigned to each of the tied values. The next step involves calculating $T_{+}$ and $T_{-}$ based on the dataset from Table 15, where $T_{+}$ = 12 and $T_{-}$ = 3. The test statistic is then determined as T = min($T_{+}$,$T_{-}$) = 3 [94].

Finally, for n = 5 (number of non-zero differences) and a two-tailed test at α = 0.05, the critical value will be determined. The value from the Wilcoxon signed-rank table is six. Since three is smaller than six, the value is *p* < 0.05, and the null hypothesis is rejected. There is significant evidence (*p* < 0.05) that CRP levels decreased after treatment. Rank-based methods are particularly advantageous for small data samples in which deviations from normality are evident and data cannot be effectively transformed. These methods are also preferred when variable transformations would result in less interpretable measurement units. While parametric tests generally offer greater statistical power when their assumptions are met, rank-based tests demonstrate increased robustness to outliers [94].

### 8.7. Fisher’s Exact Test

Fisher’s Exact Test is a non-parametric statistical test for evaluating associations between two nominal variables in 2 × 2 contingency tables. Contrary to the Chi-squared test, which is computed based on large-sample approximations, Fisher’s test provides exact *p*-values based on the hypergeometric distribution. This renders the test particularly suitable for small data sets (typically less than 20 observations) or in tables with few expected cells counts (<5), where approximate approaches may yield unreliable results [101,102].

The test is used to test the null hypothesis of independence between two variables. A statistically significant *p*-value is evidence for association, with rejection of H_0_ being conventionally at *α* = 0.05. Depending upon how the hypothesis has been formulated, the test can be one- or two-tailed. Since it conditions on fixed marginal totals (row and column), the test is conservative, which can reduce statistical power in some situations [103,104].

Outside of classical statistics, Fisher’s Exact Test finds wide application in machine learning for comparing classifying models in terms of whether differences in accuracy or error rates are significant statistically. The ISO/IEC TS 4213:2022 standard lists its application in performance measurement. Application is across all fields, from bioinformatics (gene set enrichment analysis) [105] to medicine (small-scale clinical trials).

Historically, the approach is a derivation of Ronald Fisher’s “Lady Tasting Tea” study [103], which proved useful for categorical data analysis. The main strengths over approximate tests are resistance to sparse data, exact probability assessment, and no normal distribution assumption. The weakness is inapplicability to dependent data (when McNemar is more applicable) and higher computational requirements for greater-than-2 × 2 tables [102,104,106]. These features make Fisher’s Exact Test the go-to basic statistic for exact small-sample study and classification performance analysis.

Fisher’s Exact Test is a non-parametric test based on the hypergeometric distribution that computes exact *p*-values without the assumption of large samples. It is particularly well-suited to small datasets or where contingency tables are subject to low expected frequencies. For the test to be executed, a 2 × 2 contingency table is first prepared, for example, comparing two machine learning models on the basis of classification accuracy (Table 16).

Here, n is the number of samples, and *a*, *b*, *c*, and *d* are frequencies in the tabulated table. The probability of seeing the table under the null hypothesis of independence would be given by the hypergeometric distribution (Equation (74)).

(74) $$p=\frac{\left(\frac{a+b}{a})(\frac{c+d}{c}\right)}{\left(\frac{n}{a+c}\right)}$$

To get a two-tailed *p*-value, one adds probabilities for all contingency tables with probabilities equal to or smaller than that of the observed table. The decision rule is simple [101,103] (Equation (75)).

(75) $$\mathrm{Decision}\left\{\begin{array}{c} p\;\leq 0.05\;H_{0}\;Reject\\ p>0.05\;H_{0}\;Accept \end{array}\right.$$

where the alpha level is typically set at 0.05. Results are to record the contingency table, frequency counts, actual *p*-value, and whether a one- or two-tailed test was conducted. Due to its computational requirements, program packages such as R, Python, SPSS, or SAS are typically employed, with Monte Carlo simulations for larger-than-2 × 2 tables [102,103].

The test takes independent data into account; for dependent observations (e.g., repeated measures), McNemar’s Test is to be used [102,103]. If marginal totals are kept constant, the test may be conservative, which can reduce statistical power. In such situations, alternatives like Barnard’s Test might be more powerful [103,104]. Extensions are available for large contingency tables (e.g., Bosch’s Exact Test) [106] and for stratified data (e.g., Cochran–Mantel–Haenszel Test) [103]. For transparency and replicability, there should be reporting with contingency table statistics, marginal totals, and programs utilized.

In the study of relationship between ESR1 in bone metastatic mutations and breast cancer, the investigators conducted a liquid biopsy to investigate the relationship between ESR1 mutations and bone metastases in the HR-positive subgroup of 88 patients with breast cancer. Table 17 presents the 2 × 2 frequency distribution containing data from 35 HR-positive patients with ESR1 mutations and bone metastases evaluations [107].

In order to examine whether ESR1 mutations are statistically independent from bone metastasis, a two-sided Fisher’s Exact Test was carried out using Stata (version 14.2). The resulting *p*-value was 0.022, which led to the rejection of the null hypothesis due to a significant association. These findings support the potential of ESR1 mutations as biomarkers for predicting the likelihood of bone metastasis within this subgroup.

This example demonstrates that Fisher’s Exact Test is of great utility in small-scale medical research, particularly in investigating the correlation between genomic markers (such as ESR1 mutation) and breast cancer metastases. It was precise and flexible in dealing with low-frequency data, unlike, say, the Chi-squared test, the alternative that would not have been appropriate due to small cell frequencies [107].

### 8.8. Central Limit Theorem (CLT)

The Central Limit Theorem (CLT) is an important theorem of probability theory and statistics that the mean or total of a large number of independent random variables will roughly follow a normal distribution, no matter the original variables’ distribution, under certain conditions [108,109,110]. This kind of property makes it possible for good statistical inference, including hypothesis testing and confidence interval estimation, even if the population in question is not normal. Mathematically, for mutually independent random variables $X_{1},X_{2},\ldots ,\;X_{n}$, with finite mean $\mu_{i}=E\left[X_{i}\right]$ and variance $\sigma_{i}^{2}=Var(X_{i})$, the sum $S_{n}=X_{1}+X_{2}+\ldots +X_{n}$ has variance (Equation (76)).

(76) $${Var(S}_{n})=\sigma_{1}^{2}+\sigma_{2}^{2}+\cdots +\sigma_{n}^{2}$$

Under the Lindeberg-Feller condition, the standardized sum converges in distribution to a standard normal $N\left(0,\;1\right)$ (Equation (77)).

(77) $$N\left(0,1\right)\overset{d}{\to }\frac{S_{n}-E[S_{n}]}{\sqrt{Var(S_{n})}}$$

For independent and identically distributed (i.i.d.) variables with mean μ and variance σ^2^, the sample mean is Equation (78).

(78) $${\bar{X}}_{n}=\frac{1}{n}\sum_{i=1}^{n}X_{i}$$

With μ = E[${\bar{X}}_{n}$] and $\frac{\sigma^{2}}{n}$, the standardized mean converges to standard normal (Equation (79)).

(79) $$N\left(0,1\right)\overset{d}{\to }\frac{(\mu -{\bar{X}}_{n})\sqrt{n}}{\sigma }=Z_{n}$$

This theorem holds a particularly important role in medical statistics, where it is essential in the analysis of measurement errors, in testing diagnostic models, and in verifying the stability of machine learning algorithms [108,110]. Its major advantage lies in permitting normal-based inference for non-normal and multivariate data whenever the sample size is sufficiently large, commonly taken as n ≥ 30.

The application of the CLT to independent or dependent data follows a systematic process. First, one defines the random variable of interest, such as the sample mean in Equation (78). Next, one computes the mean and variance. For i.i.d. variables, the expected value of the sample mean is μ and its variance is $\frac{\sigma^{2}}{n}$; for non-identically distributed variables, the variance of the sum is given by $Var\left(S_{n}\right)=\sigma_{1}^{2}+\sigma_{2}^{2}+\cdots +\sigma_{n}^{2}$. If $\sigma^{2}$ is unknown, it may be approximated using the sample variance s^2^. Subsequently, the Lindeberg condition is verified to ensure that the influence of extreme values diminishes as n→∞ (Equation (80)) [109,111].

(80) $$\underset{n\to \infty }{\lim}\frac{1}{s_{n}^{2}}\sum_{i=1}^{n}E\left[{\left(X_{i}-\mu_{i}\right)}^{2}.1_{\left\{\left|X_{i}-\mu_{i}\right|>{\varepsilon s}_{n}\right\}}\right]=0,\forall \varepsilon >0$$

Normalization is then performed by defining the standardized variable $Z_{n}=\frac{S_{n}-E[S_{n}]}{\sqrt{Var(S_{n})}}$, which converges to $N\left(0,\;1\right)$ asymptotically [109,111]. This allows probability calculations to be approximated using the normal distribution, according to Equation (81).

(81) $$P\left(a\leq X_{n}\leq b\right)=\Phi \left(\frac{b-n\mu }{\sqrt{n}\sigma }\right)-\Phi \left(\frac{a-n\mu }{\sqrt{n}\sigma }\right)$$

where Φ denotes the cumulative distribution function of the standard normal. For discrete observations, a continuity correction may be applied. In the final stage, hypothesis testing and confidence interval estimation are carried out. Standard Z-tests or interval estimation can be performed using the standard error $\frac{\sigma }{\sqrt{n}}$ [112].

An illustrative example of CLT in medical statistics can be found in a 1971 clinical trial investigating whether laser photocoagulation delayed blindness in patients with diabetic retinopathy. In this study, one eye from each patient received treatment while the other served as control, and the outcome was the quiescent interval time (α-QIT) to blindness for 24 patients. This design yielded a multivariate and partly dependent dataset [113].

For the treated eyes, the estimator of quiescent time was defined as Equation (82).

(82) $${\hat{q}}_{0.5,2}\left(t\right)=t_{2}-F_{n,2}^{-1}(0.5F_{n}\left(t\right);t_{1})$$

where $F_{n}\left(t\right)$ is the empirical joint distribution and $F_{n,2}^{-1}$ is the empirical inverse for the treated eye. The sample mean was then computed as Equation (83).

(83) $${\bar{q}}_{0.5,2}\left(t\right)=\frac{1}{n}\sum_{i=1}^{n}q_{0.5,2,i}\left(t\right)$$

Yielding a median blindness time of approximately 13.785 months for the treated eyes. For n = 24, ${\bar{q}}_{0.5,2}\left(t\right)$ was calculated, and in a simulated sample of n = 50, the estimate was ${\bar{q}}_{0.5,2}\left(t\right)\approx 0.0190$ with small errors. The assumption of normality for these estimates was verified using the Shapiro–Wilk test, which produced a *p*-value of 0.15, supporting the normal approximation.

From this, a 95% confidence interval was constructed. With $\sqrt{\left(s^{2}/n\right)}=\sqrt{(0.0076/50)}\approx 0.0123$, and using the critical value $z_{\frac{\alpha }{2}-1}=z_{0.975}$, the interval was given by ${\bar{q}}_{0.5,2}\left(t\right)\pm z_{0.975}\times \sqrt{\left(s^{2}/n\right)}=0.0190\pm 1.96\times 0.0123=(-0.0051,\;0.0431)$. Comparison with control eyes was carried out using Equation (84).

(84) $$d_{n}\left(t\right)={\bar{q}}_{0.5,1}\left(t\right)-{\bar{q}}_{0.5,2}\left(t\right)$$

Whereby ${\bar{q}}_{0.5,1}\left(t\right)$ and ${\bar{q}}_{0.5,2}\left(t\right)$ denote the median quiescent times for control and treated eyes, respectively. According to [112], the difference $d_{n}\left(t\right)$ provides statistical evidence that laser treatment delays blindness. The CLT ensures that as the sample size increases, the distribution of $d_{n}\left(t\right)$ approaches normality, thereby validating the inference procedure.

This example underscores the significance of the Central Limit Theorem in medical statistics. Even when the underlying distributions are non-normal or when data exhibit dependence across observations, the CLT permits approximation of the sampling distribution of the mean. As a result, confidence intervals and hypothesis tests can be constructed in a theoretically justified manner, demonstrating the essential role of CLT in medical research and the analysis of complex clinical data.

### 8.9. McNemar Test

McNemar’s Test is a non-parametric statistical method designed to assess differences between paired binary outcomes. It is especially relevant for analyzing 2 × 2 contingency tables with correlated observations, such as those arising from pretest–posttest designs, repeated measures, or comparisons of two classifiers evaluated on the same dataset. Unlike the simple Chi-square test, which assumes independence across observations, McNemar’s Test focuses specifically on discordant pairs that they are cases where one observation is classified as a success and the corresponding paired observation as a failure. The null hypothesis (H_0_) states that the probability of success is the same in both groups, implying no statistically significant difference between the paired proportions. Because of this focus, the test has found widespread use in medicine, education, clinical data analysis, and machine learning [114,115,116,117]. Several variations exist, including the classical, continuity-corrected, exact, and mid-p approaches. The mid-p version is often preferred in small to moderate sample sizes, since it balances statistical power and Type I error control [116].

The McNemar test examines paired binary data by means of a 2 × 2 contingency matrix, which summarizes the results from two related sets, as shown in Table 18.

Where a represents cases where both groups succeed, b indicates success in Group 1 but failure in Group 2, c indicates failure in Group 1 but success in Group 2, and d indicates failure in both groups. The classic test statistic (without continuity correction) is calculated as Equation (85).

(85) $$X^{2}=\frac{{\left(b-c\right)}^{2}}{b+c}$$

For small sample sizes, however, exact procedures based on the binomial distribution are recommended, with the mid-p variant offering a less conservative alternative. In this case, the mid-*p* value is computed as Equation (86) [115].

(86) $$p_{value}=2\times BinomCDF\left(b,b+c,0.5\right)-BinomPDF(b,b+c,0.5)$$

where BinomCDF represents the cumulative distribution function and BinomPDF the point probability function of the binomial distribution [114]. This approach tends to provide greater power while maintaining control over Type I error rates [117].

An applied example can be seen in a medical study that compared two machine learning models (Model A and Model B) for predicting two-year survival in 214 patients with multiple myeloma, of whom 27 were affected by the disease [114]. The objective was to determine whether the two models differed significantly in overall accuracy. The contingency table summarizing the results indicated that *a* = 165 patients were correctly classified by both models, *b* = 15 was correctly classified by Model A but not by Model B, *c* = 10 was correctly classified by Model B but not by Model A, and *d* = 24 was misclassified by both models. Based on these counts, the classical McNemar statistic was calculated as

$$X^{2}=\frac{{\left(15-10\right)}^{2}}{15+10}=\frac{5^{2}}{25}=\frac{25}{25}=1$$

When compared against the Chi-square distribution with one degree of freedom, the corresponding *p*-value was approximately 0.317. Since this exceeds the conventional significance threshold of 0.05, the null hypothesis was not rejected. Applying the mid-p version of the test, which relies on the binomial formulation, produced a *p*-value of 0.301. Both approaches therefore yielded the same conclusion; there is no statistically significant difference in accuracy between the two predictive models.

The interpretation is straightforward. Because the *p*-values from both the classical and mid-p tests were greater than *α* = 0.05, the null hypothesis (*H*_0_: *b* = *c*) was retained. Thus, Models A and B cannot be distinguished in terms of their two-year survival prediction performance in this patient cohort. The test emphasizes discordant pairs (*b* and *c*) as the sole source of information about differences between the groups, while concordant pairs (a and d) contribute no information to this comparison.

From a methodological perspective, McNemar’s Test requires that the data be both paired and dichotomous. The classical form is generally considered appropriate if the number of discordant pairs, *b* + *c*, is at least ten. In smaller samples, exact or mid-p versions are preferable in order to avoid tests that are overly conservative. While the test itself does not accommodate covariates or additional explanatory factors such as age or disease stage, extensions such as generalized linear mixed models (GLMM) can be applied to address such complexities. Nonetheless, the McNemar framework remains valuable because it preserves analytical simplicity while maintaining its central goal of testing marginal homogeneity without imposing excessive distributional assumptions.

### 8.10. Adjusted t-Test

The Adjusted *t*-test is a statistical test used in the case of comparison between two groups that are related, particularly when the usual assumptions of the common *t*-test fail or when the observations are not independent. The adjustment usually occurs in situations such as cross-validation studies or machine learning, in which the repeated measures on the same data would create dependency and lead to a variance underestimation. The adjustment remedies this relationship by modifying the standard error of the mean difference to make the *t*-test obtained more conservative and less prone to committing Type I errors [118].

For comparing two supervised learning algorithms’ performance, it is usually done by comparing them on the same data with k-fold cross-validation. Both algorithms are trained and evaluated on the same folds, and the performance measure (such as accuracy, AUC, or RMSE) is recorded for every fold. The straightforward next step is to compare the two sets of results using a paired *t*-test, since each fold provides a paired observation of the performance of the algorithms. The paired *t*-test, in the classical formulation, computes differences between algorithms per fold, estimates the mean and variance of the differences, and then builds a test statistic to determine whether the mean difference is significantly distant from zero. The formula for the test statistic is provided by Equation (87), in which $\bar{d}$ represents the average difference over folds, s is the standard deviation of differences, and k is the number of folds.

(87) tadj=d¯SK

However, this approach assumes that the results between different folds are independent, which is not the case in cross-validation. In k-fold CV, all observations are used to produce a number of training sets; thus, the errors between folds are dependent. This dependency renders the standard paired *t*-test too conservative, producing too small standard errors and inflated Type I error rates (false positives). Dietterich [82] solved this issue and proposed an adjusted paired *t*-test for k-fold cross-validation that adjusts the standard error of the mean difference for dependence between folds. The adjusted standard error is given by Equation (88). The corresponding test statistic is Equation (89).

(88) $${SE}_{CV}=s\times \sqrt{\frac{1}{k}+\frac{1}{k-1}}$$

(89) tCV=d¯SSECV

with k − 1 degree of freedom. This adjustment increases the estimated standard error relative to the naive test, making the adjusted *t*-test more conservative. In practice, this means the adjusted paired *t*-test will require a larger observed performance difference before declaring statistical significance, which decreases the likelihood of false superiority claims.

The adjusted paired *t*-test provides a method for comparing algorithms when performance is measured in terms of continuous statistics obtained from resampling methods, i.e., error rates, RMSE, log-loss, or AUC values accumulated across the cross-validation folds. Unlike the usual paired *t*-test that assumes independence between folds, the adjusted one explicitly addresses the issue that training folds overlap and thus results are correlated. By enlarging the standard error by the correction term in Equation (88).

The adjusted *t*-test is conservative and guards against making overly optimistic inferences. In real-world applications, even if a model appears to do better on average than another model, the adjusted *t*-test will not necessarily declare it significant. The improvement must be replicable across the different folds of cross-validation. Further, it must be of sufficient magnitude for the test to ascertain that the difference is statistically significant. This makes it particularly well-suited to cross-validation experiments, where overlap between folds would otherwise render naive tests misleading.

McNemar’s test, though, is for a specific data structure: paired categorical responses on the same subjects and whether two classifiers disagree significantly often. If per-subject accuracy is the question of interest, McNemar is clearly the test to apply; if the comparison is a question of aggregate measures of performance over repeated folds, the adjusted paired *t*-test is more effective. That is, McNemar directly accounts for subject-level disagreements, whereas the adjusted paired *t*-test accounts for the reliability of average performance differences between correlated cross-validation results.

Therefore, for experiments that involve heavy use of cross-validation for estimating generalization accuracy, the paired *t*-test with corrections is the more appropriate tool. It explicitly deals with resampling structure in the variance calculation and provides a principled way of determining whether the algorithms’ observed differences are stable or merely artifacts of fold overlap.

### 8.11. Accommodating Multiple Comparisons

In studies where numerous hypotheses are simultaneously tested, such as when assessing machine learning model performance or microarray gene expression, the probability of rejecting true null hypotheses increases. This has been represented by either the false discovery rate (FDR) or the familywise error rate (FWER) [119,120]. Adjustment for multiple comparisons is crucial to establish the reliability of statistical inference. Methods such as Bonferroni correction, Holm-Bonferroni stepdown procedure, and Benjamini–Hochberg FDR are applied routinely to control such error rates. Bonferroni technique divides the significance level (*α*) by the number of tests (*n*), providing a conservative bound $\frac{\alpha }{n}$ to limit FWER [121]. FWER under independence is given by Equation (90).

(90) $$FWER=1-{\left(1-\alpha \right)}^{n}$$

where α is the significance level and n are the number of tests [121]. This value is important in settings where error control is critical (such as clinical trials). FDR, defined as the expected proportion of false rejections among all rejected hypotheses, is given by Equation (91).

(91) $$FDR=E(\frac{V}{R})$$

where V is the count of false negatives and R is the count of rejected hypotheses [119,121]. FDR is particularly desirable in exploratory large-scale studies where statistical power optimization is at highest priority, while FWER-based methods are yet to be preferred in severe-error applications such as clinical trials [122,123].

Among the principal multiple-comparison procedures, the Bonferroni correction is conceptually straightforward and non-parametric, reducing the significance level to $\frac{\alpha }{n}$ for each hypothesis. While this effectively controls FWER, it is highly conservative and tends to inflate Type II error rates as n increases [121]. The Holm–Bonferroni procedure improves upon this by ordering *p*-values and applying sequential thresholds of the form $p_{i}\leq \frac{\alpha }{n-i+1}$. This approach remains conservative but offers greater power while still controlling the FWER [121]. The Benjamini–Hochberg method regulates FDR by using ordered thresholds of the form $p_{i}\leq \frac{i.q}{n}$, where q is the desired false discovery rate. This allows for a balance between error control and statistical power in large-scale testing scenarios [119,120].

A concrete example is provided by a clinical study that compared 55 patient characteristics between two groups with distinct mental health disorders [124]. Ten hypothetical *p*-values, *p*_(1)_ = 0.0001, *p*_(2)_ = 0.0002, *p*_(3)_ = 0.001, *p*_(4)_ = 0.005, *p*_(5)_ = 0.01, *p*_(6)_ = 0.03, *p*_(7)_ = 0.07, *p*_(8)_ = 0.15, *p*_(9)_ = 0.26, *p*_(10)_ = 0.52, sorted in ascending order, were used to illustrate the application of multiple-comparison procedures.

Applying the Bonferroni correction with *α* = 0.05 and *n* = 55 yielded an adjusted threshold of $\frac{\alpha }{n}=\frac{0.05}{55}\approx 0.00091$. None of the *p*-values fell below this threshold, leading to the conclusion that no patient characteristics were statistically significant. This illustrates the conservative nature of Bonferroni adjustment, which effectively prevents Type I errors but may overlook genuine differences when many hypotheses are tested.

Using the Holm–Bonferroni method under the same conditions, the sequential thresholds ($\frac{\alpha }{n-i+1}$) were applied to the ordered *p*-values. With *α* = 0.05 and *n* = 55:

- *p*_(1)_ = 0.0001 ≤ 0.05/55 ≈ 0.00091 → reject H1;
- *p*_(2)_ = 0.0002 ≤ 0.05/54 ≈ 0.00093 → reject H2;
- *p*_(3)_ = 0.001 > 0.05/53 ≈ 0.00094 → stop.

Thus, two hypotheses were rejected, identifying two significant patient characteristics. This outcome demonstrates the enhanced power of Holm–Bonferroni compared with the standard Bonferroni adjustment, while still ensuring FWER control.

The Benjamini–Hochberg procedure was then applied with *q* = 0.05 and *n* = 55. Thresholds were calculated as $\frac{i.q}{n}=\frac{i.0.05}{55}\approx i.0.00091$. The first three *p*-values satisfied their respective thresholds and leading to rejection of three hypotheses. The fourth value exceeded its threshold and the procedure stopped.

- *p*_(1)_ = 0.0001 ≤ 0.00091 → reject H1;
- *p*_(2)_ = 0.0002 ≤ 0.00182 → reject H2;
- *p*_(3)_ = 0.001 ≤ 0.00273 → reject H3;
- *p*_(4)_ = 0.005 > 0.00364 → stop.

Thus, three null hypotheses were rejected, demonstrating that FDR-based adjustment yields more discoveries than FWER-based approaches. This highlights the utility of the FDR framework in large-scale exploratory settings such as genomics and machine learning model evaluation.

The choice of correction method depends critically on study design and objectives. Bonferroni correction is most appropriate for modest numbers of tests where stringent FWER control is required. Holm–Bonferroni offers a more balanced approach by improving power while preserving FWER control. The Benjamini–Hochberg procedure is particularly advantageous in exploratory or high-dimensional research, where maximizing discoveries under controlled error rates is desirable. In small samples or where dependence between variables is present, resampling-based approaches may further enhance the robustness of error control [119,124].

## 9. Clinical Requirements for Diagnostic AI Evaluation

Clinical diagnosis is a high-stakes setting where the consequences of an algorithmic error are not limited to inconvenience or cost. A wrong prediction can delay treatment, trigger unnecessary interventions, or amplify inequities for specific patient groups. For this reason, evaluation in clinical AI is judged not only by how well a model performs on a dataset, but by whether the reported evidence is sufficient for safe use in real care pathways. Machine learning systems used for clinical diagnosis operate in a safety-critical context where errors can delay treatment, trigger unnecessary interventions, or exacerbate existing health inequities. Clinical evaluation must therefore extend beyond reporting strong performance on retrospective datasets and demonstrate that a system is reliable across diverse patient populations and real-world conditions, while remaining consistent with privacy obligations and regulatory expectations. International guidance, including the World Health Organization’s recommendations on AI for health, frames trustworthy clinical AI around human oversight, safety, transparency, equity, and accountability across the system lifecycle [125]. These principles provide a useful lens for interpreting the technical evaluation toolbox reviewed in this article. When evaluation metrics, validation strategies, and statistical tests are aligned with clinical requirements, they become more than academic reporting; they form the evidence package that supports clinical credibility and adoption.

A practical way to connect technical evaluation to clinical acceptance is to anchor every result to the intended clinical use. In diagnostic AI, intended use defines the patient population, clinical setting, and the decision that the tool supports (for example, screening, triage, second reading, or diagnostic confirmation). The same algorithm can be acceptable in one setting and unsafe in another, even if reported performance is similar. A screening tool may tolerate a different error profile than a triage tool used in emergency care. Clinicians and regulators, therefore, expect evaluation to clarify the role of AI within the diagnostic workflow and how model outputs will be acted upon. Reporting standards emphasize this need for explicit description of inputs, outputs, intended users, and the clinical pathway in which the system operates, because these details determine whether results apply to real practice [126].

### 9.1. Clinical Validity

Once the intended use is clear, the metrics reviewed in this paper can be interpreted in a clinically meaningful way. In medicine, stakeholders often prefer measures that reflect patient risk and decision consequences. This does not mean replacing advanced metrics but rather presenting them in a manner that supports clinical reasoning. For example, the confusion matrix is not merely a technical summary; it represents the expected pattern of missed diagnoses and false alarms. Confidence intervals are not only statistical decorations; they describe how stable the model’s behavior is likely to be when deployed on new patients. When prevalence differs across institutions, predictive values can vary even when sensitivity and specificity remain constant, which directly affects how clinicians interpret risk scores and decide on follow-up testing. Similarly, calibration evidence becomes essential when model outputs are presented as probabilities used to guide clinical decisions, because miscalibrated probabilities can create overconfidence and harm patient safety. These interpretation steps do not require complex mathematics; they require framing technical results as clinical evidence [127].

In clinical diagnostic studies, credibility also depends heavily on how the “ground truth” is established. Diagnostic labels in healthcare often contain uncertainty and may reflect imperfect reference standards, inter-rater variation, or evolving clinical knowledge. This reality motivates careful dataset construction, transparent label definitions, and documentation of clinical reference standards. The STARD-AI guideline emphasizes that diagnostic accuracy studies using AI must report reference standards, clinical data sources, and conditions under which results apply. This strengthens the connection between evaluation metrics and clinical validity by making clear what “correct” means and how that correctness was determined [127].

### 9.2. Generalizability and Robustness

Healthcare is a setting where a distribution shift is common. Changes in clinical coding, imaging devices, lab assays, or local protocols can alter data patterns even when the diagnostic question remains unchanged. A model that performs well in one hospital may degrade in another due to differences in patient demographics, disease prevalence, or acquisition protocols. This is why medical acceptance relies heavily on evaluation that demonstrates generalizability across sites and time. External validation provides evidence that results are not artifacts of a single institution, while temporal validation tests whether a model remains reliable as clinical practice evolves. In this context, the cross-validation strategies discussed in your review become clinically meaningful tools rather than technical options: stratification supports fairness and stable estimates across patient groups, grouped validation reduces leakage across correlated observations, and time-series validation reflects real deployment where the future cannot be sampled like the past. Reporting standards such as TRIPOD+AI also reinforce the need to describe validation design, dataset provenance, and limitations so that decision makers can judge whether performance is transferable to their patient population [128].

Robustness metrics, such as agreement measures and correlation-based evaluations, become particularly valuable when diagnostic signals are noisy or when outcomes are continuous or ordinal. Clinicians also care about performance stability under realistic conditions such as incomplete data, poor image quality, uncommon presentations, and workflow interruptions. Robustness evaluation can therefore be framed as evidence that the system behaves predictably when clinical inputs deviate from “ideal” research conditions. In practice, this kind of evaluation supports safe adoption because it helps identify failure modes before deployment. This aligns with WHO’s emphasis on safety, reliability, and risk awareness across the AI lifecycle [129].

### 9.3. Statistical Significance and Reproducible Evidence

Statistical testing plays a distinct role in clinical evaluation because clinical decision-making is guided by evidence-based medicine standards. It is not enough to show that a model performs well; one must show that improvements over baselines are reliable and not due to chance, data leakage, or overfitting. This is where the statistical significance tests discussed in your review connect directly to clinical credibility. Paired designs and non-parametric comparisons are often appropriate when the same patients are evaluated under different models or conditions, and corrections for multiple comparisons help avoid inflated claims when many metrics, subgroups, and thresholds are tested. In clinical papers, transparent reporting of uncertainty and significance is one of the main signals that results are robust enough to inform clinical decisions, rather than merely suggesting promise. STARD-AI and TRIPOD+AI support this broader goal by encouraging complete reporting of study design, evaluation strategy, and limitations [127].

### 9.4. Prospective Evaluation and Real-World Clinical Utility

Retrospective validation is a necessary but incomplete form of evidence for diagnostic AI [130]. Clinical deployment occurs under time pressure, incomplete patient information, interruptions in workflow, and heterogeneous acquisition conditions, meaning that offline test performance can substantially overestimate real-world reliability [131]. For this reason, clinical translation requires prospective evaluation designs that measure model behavior under operational conditions consistent with its intended use [126,132]. Prospective evaluation can be conducted in silent deployment (shadow mode), where predictions are generated but not shown to clinicians, enabling unbiased detection of calibration drift and distribution shift without influencing care decisions [133]. Alternatively, prospective evaluation can be interventional, measuring how AI outputs change clinician decisions, diagnostic timing, downstream testing, and ultimately patient outcomes [126]. This distinction is essential because clinical utility is not determined solely by diagnostic accuracy, but by whether the AI system improves decision-making without introducing new harms such as alert fatigue, workflow overload, or automation bias [134]. In this setting, reporting frameworks such as SPIRIT-AI and CONSORT-AI provide guidance for documenting trial design and AI integration, ensuring that evidence remains decision-relevant and reproducible [126,132].

### 9.5. External Validity Across Sites and Multicenter Evidence

Single-center evaluation provides limited evidence for clinical adoption because diagnostic AI models are vulnerable to institutional artifacts such as site-specific acquisition protocols, scanner properties, coding habits, and documentation practices [135]. External validity, therefore, requires evaluation on independent cohorts, preferably across multiple institutions, with site-stratified reporting rather than pooled averages alone [135]. Multicenter validation provides stronger evidence that performance reflects disease-related signal rather than dataset-specific correlations [136]. Temporal validation is equally important because clinical practice evolves, devices change, and prevalence shifts, meaning that a model trained on historical data may degrade silently when deployed. These requirements align with the TRIAGE framework’s emphasis on generalizability testing and with TRIPOD-AI and STARD-AI reporting principles, which require a clear description of dataset provenance, validation design, and the conditions under which diagnostic accuracy claims apply [127,129]. In high-stakes settings, external validation is not an optional improvement but a primary safety requirement because failure across sites translates directly into inequitable clinical outcomes [137].

### 9.6. Reference Standard Uncertainty and Ground Truth Reliability

A fundamental clinical limitation is that “ground truth” is often uncertain [138]. Diagnostic labels may reflect imperfect tests, incomplete follow-up, inter-rater disagreement, or evolving clinical knowledge, meaning that evaluation results can be distorted by reference standard error. This uncertainty can inflate apparent model error rates or, more critically, mask subgroup-specific bias if label noise is correlated with demographics or access to diagnostic procedures. A model may appear inaccurate in a subgroup due to biased underdiagnosis in the reference standard, or conversely, may appear accurate because it reproduces historical clinical bias rather than detecting true disease. Clinical evaluation must therefore document how labels were generated (expert consensus, adjudication panels, pathology confirmation, gold-standard tests, or longitudinal confirmation) and justify why the reference standard is appropriate for the intended clinical use [127,138]. STARD-AI explicitly requires transparent reporting of reference standards and applicability conditions, but clinical credibility also benefits from quantifying uncertainty through agreement analysis and sensitivity testing under alternative labeling assumptions [127]. These practices strengthen fairness interpretation, calibration interpretation, and clinical validity by clarifying what “correct” means in real diagnostic environments.

### 9.7. Dataset Shift Surveillance and Post-Deployment Monitoring

Even strong external validation does not guarantee long-term safety because healthcare data distributions shift continuously due to changes in diagnostic guidelines, clinical coding systems, devices, patient demographics, laboratory assays, and emerging diseases [139]. Clinical evaluation must therefore include a post-deployment surveillance plan that monitors both data drift and performance drift [140]. Because ground truth labels may not be immediately available in deployment, monitoring must incorporate proxy indicators such as shifts in predicted risk distributions, confidence profiles, and input feature statistics, combined with periodic revalidation on newly collected cohorts [141]. Surveillance must also be subgroup-aware: aggregate stability can conceal drift within vulnerable populations, potentially amplifying inequities over time. This lifecycle framing is consistent with WHO guidance emphasizing safety and accountability, and it aligns with ISO/IEC 23894 and NIST AI RMF governance principles, which treat risk management as continuous across deployment, maintenance, and updates. Similarly, ISO/IEC 42001 reinforces that monitoring should be institutionalized as part of an AI management system with defined accountability, documentation, and corrective action triggers [142,143].

### 9.8. Clinical Risk Management and Safety-Driven Evidence Packaging

Diagnostic AI evaluation must ultimately answer a safety question whether the evidence supports use without introducing unacceptable harm. This requires risk-managed evaluation rather than metric-driven benchmarking. False negatives and false positives correspond directly to delayed treatment and unnecessary intervention, meaning that sensitivity, specificity, predictive values, and decision thresholds must be interpreted as clinical risk tradeoffs rather than abstract statistics [144]. Risk management principles formalized in ISO 14971 provide a natural framework for mapping model error modes to clinical hazards, estimating severity and likelihood, and specifying controls such as robustness testing, fairness auditing, threshold optimization, and monitoring [145]. Broader governance standards, including ISO 31000 and ISO/IEC 23894, further justify treating dataset construction, sampling decisions, and validation design as managed risk processes rather than neutral research steps [142,146]. In this context, quality management principles such as ISO 9001 support traceability and objective evidence, implying that annotation procedures, preprocessing steps, and evaluation pipelines should be documented as controlled processes [147].

This risk-driven framing also clarifies why robustness and fairness tests are clinically essential. Robustness stress tests identify conditions under which the model fails (noise, missing data, rare-case presentations, degraded imaging quality), while fairness analysis ensures that failure is not concentrated in protected subgroups [148]. These evaluations should be treated as safety controls that define deployment boundaries, rather than optional add-ons to performance reporting. When failure modes are identified, they must be translated into clinical constraints (e.g., device limitations, patient subgroup restrictions, or workflow conditions) and explicitly reported.

### 9.9. Transparent Clinical Reporting and Reproducibility Standards

Clinical credibility depends not only on evaluation rigor but also on whether evidence is reported in a form that clinicians and regulators can audit [126,127]. Diagnostic AI papers frequently omit essential details such as threshold selection, missing-data handling, subgroup analysis, uncertainty reporting, and dataset provenance, making performance claims difficult to interpret [129]. Reporting standards, therefore, function as clinical safety enablers by reducing hidden bias, leakage, and irreproducible methodology. STARD-AI and TRIPOD-AI provide structured guidance for diagnostic and prediction model reporting, while PROBAST and PROBAST-AI support systematic assessment of bias risk in prediction modeling studies [127,129,149]. In prospective and interventional contexts, SPIRIT-AI and CONSORT-AI provide complementary guidance for trial reporting and AI integration [126,127]. These standards reinforce that reproducibility is not merely academic: independent verification is a clinical trust requirement. Documentation should therefore include model versioning, preprocessing pipelines, dataset construction logic, validation strategy, confidence intervals, and limitations, ensuring that evidence is transferable and decision-ready.

### 9.10. Deployment Readiness, Cybersecurity, and Human Oversight

Deployment readiness requires demonstrating that the model can function safely within clinical infrastructure, including acceptable latency, compatibility with hospital IT systems, and stable performance under incomplete or imperfect inputs [150]. Human oversight must be explicitly defined because diagnostic AI systems operate as decision-support tools rather than autonomous clinicians. Clinical evaluation should therefore examine usability, interpretability, clinician response patterns, and the potential for automation bias [134]. These requirements align with WHO’s human-centered trustworthiness principles and are increasingly reflected in regulatory expectations for high-risk AI.

Deployment readiness also includes cybersecurity and privacy. Clinical AI systems require secure handling of datasets, metadata, model artifacts, and update pipelines. Standards such as ISO/IEC 27001 provide a basis for confidentiality and access control [151], while industrial cybersecurity guidance such as ISA/IEC 62443 becomes relevant when models are deployed on edge devices integrated into clinical environments. Supply chain security frameworks such as NIST SP 800-161 and practices such as SBOM documentation support transparency and risk management of dependencies, reducing the likelihood that software compromise could translate into clinical harm. These concerns reinforce that clinical readiness is a socio-technical requirement: a model can be accurate yet unsafe if deployed in an insecure or poorly governed environment [152,153,154].

### 9.11. Clinical Evaluation as a Lifecycle Evidence Pipeline (TRIAGE-Aligned)

Taken together, these clinical requirements imply that diagnostic AI evaluation should be structured as a lifecycle evidence pipeline rather than a disconnected list of metrics. The TRIAGE framework provides a coherent spine: intended use definition anchors evaluation, reference standard documentation defines clinical validity, internal and external validation establish reliability, calibration and threshold optimization translate probabilities into decisions, robustness and fairness testing define safe operating boundaries, statistical testing supports evidence-based claims, reporting standards ensure reproducibility, and monitoring enables post-market safety. This structure aligns naturally with ISO/IEC governance standards such as ISO/IEC 42001 and risk frameworks such as ISO/IEC 23894 and NIST AI RMF, all of which emphasize continuous lifecycle accountability [142,143].

This pipeline framing also addresses a common weakness in AI evaluation literature: strong retrospective performance is often presented as evidence of readiness, while clinical translation requires prospective evidence, multicenter validation, explicit handling of reference-standard uncertainty, and surveillance for dataset shift. By explicitly positioning robustness, fairness, calibration, and statistical testing as safety controls within a TRIAGE-aligned workflow, the evaluation toolbox reviewed in this article becomes clinically interpretable and regulator-relevant. In this sense, the clinical section should not be treated as a concluding commentary but as the organizing logic that determines why specific metrics and standards were selected and how they support decision-ready clinical evidence.

## 10. Conclusions

Diagnostic AI evaluation must be treated as a clinical evidence process rather than a technical reporting exercise. In this narrative guidance review, we introduced TRIAGE (Trustworthy Reporting and Assessment for Clinical Gain and Effectiveness of AI models) as a structured framework that connects performance metrics, validation strategy, calibration, robustness testing, and reporting standards to the realities of clinical diagnostic workflows. TRIAGE emphasizes that discrimination metrics alone are insufficient unless they are interpreted through prevalence-aware measures (e.g., predictive values and likelihood ratios), supported by uncertainty quantification, and accompanied by calibration evidence when probabilistic outputs influence decisions. It further positions external validation, subgroup-aware fairness assessment, reference-standard transparency, and post-deployment monitoring readiness as essential components of credible diagnostic evidence. A structured checklist of the TRIAGE evaluation parameters introduced throughout this paper is provided as an appendix and is publicly available at Supplementary File S1.

In practice, TRIAGE encourages authors and evaluators to begin by explicitly defining intended use and then selecting minimum evidence appropriate to that use case. For screening systems, minimum acceptable evidence should prioritize high sensitivity with clinically interpretable false-positive burden, calibration, and external validation across representative populations. For TRIAGE tools, evidence must additionally demonstrate safe prioritization performance under realistic prevalence and workflow constraints, including robustness to missing or degraded inputs and monitoring plans for dataset shift. For second-reading systems, minimum evidence should include head-to-head comparison with clinician baselines, reader-assist evaluation where feasible, and subgroup consistency to avoid amplifying inequities. For confirmatory diagnostic decision support, the highest level of evidence is required, including strong external and temporal validation, well-defined reference standards, clinically justified threshold selection, and prospective or interventional evaluation demonstrating that model use improves decision-making without introducing unacceptable harm. By organizing evaluation in this intended-use-driven way, TRIAGE provides a practical path for producing diagnostic AI evidence that is transparent, clinically interpretable, and suitable for translation into real-world care.

## Figures and Tables

**Table 1:** Confusion matrix with diagnostic categories and error types [1].

Total Population	Condition Positive (CP)	Condition Negative (CN)
**Prediction positive**	True positive (TP)	False positive (FP) or type I error
**Prediction negative**	False negative (FN) or type II error	True negative (TN)

**Table 3:** Multi-label classification evaluation metrics.

Type	Definition	Application	Example Metrics
**Example-based metrics**	Evaluates model performance for each sample, comparing predicted vs. actual label sets for each sample.	Measuring overall prediction accuracy and correctness per sample. Sensitive to label imbalance.	Accuracy, Precision, Recall, F-Measure, Hamming Loss, Exact Match Ratio, Jaccard Index [35,36]
**Label-based metrics**	Evaluates model performance for each label across all samples.	Identifying how well the model learns each label. Detecting label-specific bias. Disregarding the interaction of labels in samples.	Macro-Precision, Micro-Recall, Chi-square distance [35,36]
**Ranking-based metrics**	Assesses whether relevant labels are ranked higher than irrelevant ones for each sample.	Useful for models with label scores or probabilities as output. Requires label confidence scores.	Ranking Loss, Average Precision, One-error, Coverage [35,36]

**Table 4:** Output sample of a multi-label classification model using Hamming loss calculation.

Patient #*i*	*Y_i_*	*Z_i_*
#1	[1, 0, 1, 0]	[1, 1, 0, 0]
#2	[0, 0, 1, 1]	[0, 1, 0, 1]

**Table 5:** Output sample of a multi-label classification model using exact match ratio calculation.

Patient #*i*	*Y_i_*	*Z_i_*
#1	[1, 0, 1, 0]	[1, 1, 0, 0]
#2	[0, 0, 1, 1]	[0, 1, 0, 1]
#3	[1, 0, 0, 1]	[1, 0, 0, 1]
#4	[0, 1, 1, 0]	[1, 1, 1, 0]

**Table 6:** Output sample of a multi-label classification model using Jaccard Index calculation.

Patient #*i*	*T_i_*	*P_i_*
#1	[1, 0, 1, 0]	[1, 1, 1, 0]
#2	[0, 0, 1, 1]	[0, 0, 0, 1]

**Table 7:** Distribution-based evaluation metrics in multi-label classification.

Metric	Definition	Use	Method
**Kullback** **–** **Leibler (KL) Divergence**	Measuring the deviation of the predicted label distribution from the true distribution.	Comparing probabilistic outputs. Sensitive to zero values and asymmetry. Useful in model calibration.	DKL=∑Pi×logPiTi note: 0×log0x=0
**Wasserstein Distance** **(Earth Mover’s Distance)**	Measuring the required transform for one distribution into the correct one.	Effective for measuring fairness or distributional shifts. Useful for ordinal labels.	$W\left(P,Q\right)=\underset{\gamma \in \prod \left(P,Q\right)}{\inf}E_{\left(x,y\right)\sim \gamma }\left[d(x,y)\right]$
**Chi-square Test (X^2^)**	Statistical test of significant differences between predicted and actual label frequencies.	Suitable for discrete count comparisons. Suitable for hypothesis testing. Need a large sample size per label.	$X^{2}=\sum_{i=1}^{n}\frac{{\left(Q_{i}-E_{i}\right)}^{2}}{E_{i}}$

**Table 8:** Interpretation of Cohen’s kappa [73].

Kappa’s Value	Level of Agreement	% of Reliable Data
**0–0.20**	None	0–4%
**0.21–0.39**	Minimal	4–15%
**0.40–0.59**	Weak	15–35%
**0.60–0.79**	Moderate	35–63%
**0.80–0.90**	Strong	64–81%
**Above 0.90**	Almost Perfect	82–100%

**Table 9:** Comparison of cross-validation methods.

CV Method	Best Use Case	Pros	Cons	Suitable for Small Datasets	Handles Imbalanced Data	Data Leakage Protection	Computational Cost
Hold-Out	Large datasets, quick evaluation	Simple, fast	The unstable estimate may waste data, no class balance unless stratified	No	No	No	Low
K-Fold	General-purpose, small/medium datasets	Reasonable performance estimate, reduced bias	Computationally expensive, sensitive to k	Yes	No (exception: Stratified K-Fold)	No	Medium
Leave-One-Out (LOOCV)	Very small datasets, medical/biological studies	Nearly unbiased, uses all data	Very slow on large datasets	Yes	Yes	Yes	High
Leave-P-Out (LPOCV)	Highly precise evaluation, tiny datasets	Extremely thorough, low bias	Combinatorially expensive, impractical on large datasets	Yes	Yes	Yes	Very High
Monte Carlo	Large datasets, assessing model stability	Flexible, can repeat many times	Results vary by split, overlapping samples, less repeatable	Yes	No	No	Medium
Time-Series	Time-dependent data (e.g., patient monitoring)	Respects temporal order, prevents look-ahead bias	Limited early training data, concept drift over time	No	No	Yes	Medium to High
Stratified CV	Classification with class imbalance	Maintains class ratio, better generalization	More complex, tricky with multilabel or tiny datasets	Yes	Yes	No	Medium
Grouped CV	Grouped/linked data (e.g., patients, sessions)	Prevents data leakage, better generalization in group scenarios	Fewer splits possible, and uneven group sizes may reduce stability	Yes	Yes	Yes	Medium

**Table 10:** Data specific to the ten patients [85].

No.	Gender	Age Group	BMI	SBP (B/L)	SBP (30 min)	SBP (60 min)
**1**	M	2	22	70	76	72
**2**	F	1	28	82	85	80
**3**	M	3	30	88	91	86
**4**	F	2	23	73	78	74
**5**	M	1	27	84	93	86

**Table 11:** A medical example for calculating the ANOVA test.

Subject	Gender	Treatment Type 1	Treatment Type 2	Treatment Type 3
**1**	Female	5	6	9
**2**	Male	10	4	8
**3**	Female	7	4	5
**4**	Male	7	3	6
**5**	Female	6	3	7
	Group Means	7	4	7
	Grand Mean	6		

**Table 12:** Summary of ranks for the Kruskal–Wallis test.

Groups	Value	Ranks	Sum of Ranks
**Group 1**	5, 7, 8	4, 6, 7	17
**Group 2**	6, 9, 10	5, 8, 9	22
**Group 3**	1, 2, 3	1, 2, 3	6
**Total N = 9**			

**Table 13:** Notation represents the number of persons in each category of the 2 × 2 table for a binary outcome variable across the two exposure groups.

	Event: Yes (d)	Event: No (h)	Total
**Group 1 (exposed)**	*d*_1_	*h*_1_	*n*_1_
**Group 0 (unexposed)**	*d*_0_	*h*_0_	*n*_0_
**Total**	*d*	*h*	*n*

**Table 14:** A 2 × 2 table displaying results from a COVID-19 drug trial.

**(a) Observed Numbers**			
	COVID-19		Total
	Yes	No	
**Drug**	10	200	250
**Placebo**	95	166	205
**Total**	105	366	455
**(b) Expected Numbers**			
	COVID-19		Total
	Yes	No	
**Drug**	57.7	201.1	250
**Placebo**	47.3	164.9	205
**Total**	105	366	455

**Table 15:** C-reactive protein (CRP) levels in nine patients before and after a 6-week anti-inflammatory treatment.

	CRP Level		Difference *d*_i_ = *y*_i_ − *x*_i_	Absolute Difference	Rank of Absolute Difference (Sign)
Patient	Before Treatment (*y*_i_)	After Treatment (*x*_i_)			
**1**	28	20	+8	8	4 (+)
**2**	25	19	+6	6	3 (+)
**3**	27	31	−4	4	2 (−)
**4**	35	21	+14	14	5 (+)
**5**	30	32	−2	2	1 (−)

**Table 16:** 2 × 2 contingency table for Fisher’s Exact test.

	Model B: Correct	Model B: Incorrect	Total
**Model A: Correct**	a	b	a + b
**Model A: Incorrect**	c	d	c + d
**Total**	a + c	b + d	n = a + b + c + d

**Table 17:** Contingency table for breast cancer diagnosis.

	Bone Metastasis: Yes	Bone Metastasis: No	Total
**ESR1 Mutation: Yes**	10	0	10
**ESR1 Mutation: No**	13	12	25
**Total**	23	12	35

**Table 18:** 2 × 2 contingency table for McNemar test.

	Group 2: Success	Group 2: Failure
**Group 1: Success**	a	b
**Group 1: Failure**	c	d

## Data Availability

No new data were created or analyzed in this study.

## Supplementary Materials

The following supporting information can be downloaded at: https://www.mdpi.com/article/10.3390/diagnostics16050666/s1. Supplementary File S1: Summary of evaluation metrics including definitions, advantages, limitations, appropriate use, and inappropriate use; Table S1: Mapping of TRIAGE evaluation components to relevant ISO/IEC standards, reporting guidelines, and regulatory frameworks [155].

## Abbreviations

AI	Artificial Intelligence
AUC	Area Under the Curve
AUROC	Area Under the ROC Curve
CAD	Coronary Artery Disease
CI	Confidence Interval
CN	Condition Negative
CP	Condition Positive
CV	Cross-Validation
DOR	Diagnostic Odds Ratio
EHR	Electronic Health Records
FDR	False Discovery Rate
FN	False Negative
FNR	False Negative Rate
FOR	False Omission Rate
FP	False Positive
FPR	False Positive Rate
IoU	Intersection over Union
KL	Kullback–Leibler (standard; used as “Kullback-Leibler”)
LOOCV	Leave-One-Out Cross-Validation
LR-	Negative Likelihood Ratio
LR+	Positive Likelihood Ratio
MAE	Mean Absolute Error
MCC	Matthews Correlation Coefficient
ML	Machine Learning
MSE	Mean Squared Error
NPV	Negative Predictive Value
PPV	Positive Predictive Value
RMSE	Root Mean Square Error
ROC	Receiver Operating Characteristic (curve)
SE	Standard Error
SVM	Support Vector Machine (standard; used as “SVM”)
TN	True Negative
TNR	True Negative Rate
TP	True Positive
TPR	True Positive Rate
VAE/VAEs	Variational Autoencoder(s)
