# Supplementary material for: TRIAGE: Trustworthy Reporting and Assessment for Clinical Gain and Effectiveness of AI Models
Source: Diagnostics (Basel). 2026 Feb 25;16(5):666. doi: 10.3390/diagnostics16050666 (PMC12984829; doi:10.3390/diagnostics16050666)
Supplement: Supplementary file 1 [file diagnostics-16-00666-s001.zip › Supplementary File S1.pdf]

Supplementary File S1. Comprehensive Summary of Evaluation Metrics, Curves, Validation Strategies, and Statistical Tests for Diagnostic AI (TRIAGE Framework Mapping)

Appendix A provides a structured and comprehensive summary of all evaluation components discussed in this review, including performance metrics, representation curves, robustness and fairness measures, validation strategies, computational efficiency indicators, and statistical significance tests. The table is intended to serve as a practical reference for researchers and clinicians by clarifying (i) what each evaluation tool measures, (ii) its primary advantages, (iii) common limitations and sources of misinterpretation, and (iv) the clinical scenarios in which it is appropriate or inappropriate.

This appendix supports the TRIAGE framework by linking technical evaluation methods to clinical evidence requirements such as intended use definition, reference standard reliability, external validity across sites, prospective evaluation, and post-deployment monitoring. The purpose of this summary is not to propose a single "best" metric, but to highlight that metric selection must be aligned with the diagnostic workflow, the clinical consequences of false positives and false negatives, and the deployment context.

To improve transparency and reproducibility, this appendix is designed to complement the narrative review by providing an explicit inventory of the evaluation toolbox described in the manuscript and by reducing the risk of selective reporting or over-reliance on single summary measures such as accuracy or AUC, based on AI standards, guidelines and papers.

**Section 2.1 — Binary & Multi-class classification**					
**Metric / Measure**	**Definition**	**Pros**	**Cons**	**Appropriated Use**	**Unappropriated Use**
**Confusion-matrix counts (TP, TN, FP, FN)**	Raw counts of correct/incorrect predictions vs reference labels	Fully transparent; enables *all* derived diagnostic metrics; helps interpret error modes	Can hide severity/cost differences unless paired with clinical context; depends on threshold and reference standard quality	Always report (binary and multi-class) as the base for interpretable evaluation	Alone is not "performance"; avoid without derived measures and context
**Prevalence**	Proportion of positives in the evaluated population	Essential for interpreting PPV/NPV and clinical workload implications	If prevalence differs from target setting, predictive values will not transport	Any clinical/diagnostic reporting where PPV/NPV are discussed	Don't imply PPV/NPV generalize if prevalence shifts across sites/settings
**TPR / Sensitivity / Recall**	Fraction of true positives correctly detected	Clinically intuitive; aligns with "miss" risk (FN)	Can look "good" while FP burden is unacceptable; threshold-dependent	Screening/triage contexts where missing disease is high harm	Avoid interpreting without specificity/PPV and the intended operating threshold

**TNR / Specificity / Selectivity**	Fraction of true negatives correctly rejected	Clinically intuitive; reflects unnecessary alarms/workup	Can look “good” while sensitivity is inadequate	Confirmation contexts; avoiding unnecessary follow-up/testing	Don’t use alone to claim clinical safety—pair with sensitivity and prevalence
**FPR (fall-out)**	Fraction of negatives incorrectly labeled positive	Directly reflects alarm burden	Often misunderstood; depends on threshold; may understate burden when negatives are large	When estimating downstream work, false alarms, unnecessary follow-up	Alone doesn’t capture miss risk (FNs)
**FNR (miss rate)**	Fraction of positives incorrectly labeled negative	Directly reflects missed cases	Can be minimized by lowering threshold, which can explode FPR	When missed disease is critical (safety-oriented assessment)	Alone ignores downstream burden and overtreatment risk
**PPV / Precision**	Among predicted positives, fraction truly positive	Answers the clinician’s “If model says positive, how often is it true?”	Strongly prevalence-dependent; can drop sharply in low-prevalence deployment	High-stakes “rule-in” decisions; estimating follow-up yield	Don’t transport PPV across sites without accounting for prevalence/spectrum shift
**NPV**	Among predicted negatives, fraction truly negative	Useful for “rule-out” reassurance	Also prevalence-dependent; can be inflated when disease is rare	Triage/rule-out pathways and patient reassurance contexts	Don’t interpret without prevalence and the clinical consequences of false negatives
**Accuracy**	Fraction correctly classified overall	Easy to understand; single-number summary	Misleading with class imbalance; hides FP vs FN trade-offs	Balanced datasets or when classes and costs are similar	Avoid for rare disease screening without additional metrics
**FDR**	Among predicted positives, fraction that are false positives	Directly expresses “how many alarms are wrong”	Prevalence- and threshold-sensitive; can be unintuitive to some readers	Reporting false-alarm rate in positive calls; lab/triage workflow impact	Don’t use without PPV/threshold description; can confuse if not defined clearly
**FOR**	Among predicted negatives, fraction	Directly expresses “how many negatives	Prevalence- and threshold-sensitive	When assessing safety of negative	Needs careful clinical framing; not a

	that are false negatives	are actually missed disease”		predictions (miss risk among “ruled out”)	replacement for sensitivity/NPV
**LR+ (Positive likelihood ratio)**	How much a positive result increases odds: TPR/FPR	Clinically interpretable via Bayes; supports decision thresholds	Can be unstable with small denominators; still depends on operating point	Diagnostic testing analogies; translating test results into post-test odds	Not meaningful without clearly defined threshold and population
**LR− (Negative likelihood ratio)**	How much a negative result decreases odds: FNR/TNR	Useful for “rule-out” strength	Same stability/threshold issues as LR+	Rule-out contexts; connecting to post-test probability	Avoid if TNR or TPR near extremes with small samples (unstable estimates)
**DOR (Diagnostic Odds Ratio)**	Single-number odds summary combining sensitivity & specificity	Compact; compares discriminatory ability in one value	Loses directionality of errors (FP vs FN); can hide clinically unacceptable trade-offs	Quick comparative summaries across models/tests	Do not use alone for clinical claims; must accompany operating-point metrics
**Fβ score (incl. F1 when β=1)**	Weighted harmonic mean of precision and recall; β emphasizes recall	Useful under imbalance; tunable to reflect FN vs FP importance	Can conceal whether errors are mostly FP or FN; depends on threshold/prevalence	When you must balance precision/recall (e.g., rare disease detection)	Avoid as the only headline metric; always also report component rates/PPV/NPV
**Micro-averaged precision/recall/F1**	Aggregate TP/FP/FN across classes then compute metrics	Reflects per-sample performance; good when class frequency matches deployment	Dominated by majority class; can mask rare-class failures	When overall case-level performance is primary and class mix is realistic	Use caution for rare diagnoses where minority-class errors are clinically critical
**Macro-averaged precision/recall/F1**	Compute per-class metric then average equally across classes	Highlights minority-class performance; fairness across classes	Can understate performance on common classes; may not reflect real workload	When consistent performance across all disease subtypes is required	If clinical importance is proportional to prevalence (not always equal), interpret carefully
**Weighted-averaged precision/recall/Fβ**	Per-class metrics weighted by class support	Aligns with real-world class distribution; useful “overall” metric	Can still mask poor minority-class performance	When deployment class proportions are known/representative	Don’t rely on weighted averages alone if rare classes are high-risk clinically

**Confidence intervals (general concept)**	Quantifies uncertainty around estimated metrics	Supports statistically defensible comparisons; improves interpretability	Often omitted; wrong method can mislead	Any clinical AI evaluation (especially with limited sample size)	Don't present point estimates without uncertainty in high-stakes claims
**Z-based CI (large n / known σ)**	CI using normal approximation	Simple; common	Invalid if assumptions fail; poor for small n/extreme proportions	Large samples where approximation is justified	Small sample sizes; rare events; extreme metrics near 0 or 1
**t-based CI (small n / unknown σ)**	CI using t distribution	Handles unknown variance; standard approach	Still assumption-sensitive; not ideal for proportions/imbalance extremes	Small-sample mean estimates (as described)	For classification proportions (precision/recall/Fβ), analytical formulas can be unreliable
**Bootstrap CI (percentile method)**	Resampling-based CI for metrics like F1	Works when analytic formulas are unreliable; flexible	Must respect data structure (e.g., patient-level clustering); computational cost	Precision/recall/Fβ uncertainty; small or skewed datasets	Don't bootstrap naïvely if samples are correlated (e.g., multiple images per patient)
**Section 2.2 — Multi-label classification models**					
**Metric / Measure**	**Definition**	**Pros**	**Cons**	**Appropriated Use**	**Unappropriated Use**
**Hamming Loss**	Fraction of labels incorrectly predicted (averaged per label)	Simple; penalizes each label error equally; works with many labels	Treats all labels as independent; ignores label co-occurrence; may underestimate clinical impact	Problems where each label has similar importance (e.g., multiple findings reported independently)	When label dependencies or clinical severity differ substantially
**Subset Accuracy (Exact Match Ratio)**	Fraction of samples where *all* labels are correctly predicted	Very strict; reflects full diagnostic correctness	Overly pessimistic; drops sharply as label count increases	Small label sets; tasks where complete correctness is mandatory	Large label spaces; exploratory or assistive systems
**Jaccard Index (Intersection over Union)**	Overlap between predicted and true label sets	Intuitive; balances false positives and negatives	Sensitive to rare labels; ignores label importance	Multi-pathology detection; overlapping diagnoses	When labels have very different clinical weights

**Precision (multi-label)**	Proportion of predicted labels that are correct	Reflects false-positive burden	Ignores missed labels; prevalence-dependent	Workflows sensitive to over-diagnosis	Stand-alone reporting without recall
**Recall (multi-label)**	Proportion of true labels correctly detected	Reflects missed findings	Ignores false positives	Safety-critical detection tasks	Stand-alone reporting without precision
**F1 / Fβ (multi-label)**	Harmonic mean of precision and recall	Balances FP/FN trade-off; tunable (β)	Can hide whether errors are FP- or FN-driven	Rare disease detection; imbalanced label sets	As a single headline metric without components
**Micro-averaged Precision/Recall/F1**	Aggregates label decisions across all samples	Reflects overall system behavior; stable	Dominated by frequent labels	When label frequency mirrors deployment	When rare labels are clinically critical
**Macro-averaged Precision/Recall/F1**	Equal weight to each label	Highlights rare labels; fairness-oriented	Can over-emphasize clinically minor labels	When each diagnosis is equally important	When clinical impact differs strongly by label
**Weighted-averaged Precision/Recall/F1**	Label metrics weighted by prevalence	Matches real-world workload	Masks minority-label failures	Known and stable label prevalence	Safety-critical rare conditions
**Label Cardinality**	Average number of labels per instance	Describes task complexity	Descriptive only; not a performance metric	Characterizing datasets	Performance claims
**Label Density**	Label cardinality normalized by total labels	Enables dataset comparison	Dataset-specific; no direct clinical meaning	Comparing multi-label datasets	Model evaluation
**Ranking Loss**	Fraction of label pairs incorrectly ranked	Uses ranking information; threshold-free	Hard to interpret clinically	Risk-ranking or prioritization tasks	Binary decision pipelines
**Coverage Error**	How far down ranked labels must be searched to include all true labels	Evaluates ranking depth	Not intuitive clinically	Decision-support with ranked outputs	Hard classification use cases
**One-error**	Whether top-ranked label is incorrect	Focuses on first suggestion	Ignores remaining labels	Assistive systems showing one key finding	Full diagnostic reporting
**Label imbalance measures**	Distribution skew across labels	Highlights bias risk	Descriptive only	Planning evaluation strategy	Performance reporting

**Per-label confusion matrices**	Error structure per diagnosis	Clinically interpretable	Increases reporting complexity	Safety-critical multi-pathology systems	When label count is very large without summarization
**Bootstrap CI (multi-label metrics)**	Uncertainty of complex metrics	Distribution-free; flexible	Computational cost; dependence issues	Small samples; complex metrics (F1, Jaccard)	Correlated samples without cluster-aware resampling
**Section 3 — Evaluation Representation Curves**					
**Curve / Plot / Representation**	**Definition**	**Pros**	**Cons**	**Appropriated Use**	**Unappropriated Use**
**ROC Curve (Receiver Operating Characteristic)**	Plots True Positive Rate vs False Positive Rate across thresholds	Widely used; threshold-independent view; easy model comparison	Can appear optimistic for imbalanced/rare disease tasks; may hide low PPV; does not reflect prevalence	Diagnostic discrimination reporting; comparing classifiers; general binary evaluation	Rare disease screening if used alone; should be paired with PR curve and PPV/NPV
**AUC-ROC (Area Under ROC Curve)**	Single-number summary of ROC discrimination	Simple comparison; interpretable ranking performance	May not reflect clinically relevant operating point; not directly tied to clinical utility	General model comparison, early benchmarking	Clinical deployment decisions without calibration/threshold analysis
**Precision–Recall (PR) Curve**	Precision vs recall across thresholds	More informative for imbalanced datasets; focuses on positive class performance	Less intuitive for some clinicians; depends on prevalence	Rare disease detection, screening, triage tasks	Balanced tasks where ROC is sufficient; comparisons across datasets with different prevalence
**Average Precision (AP)**	Area-like summary of PR curve	Compact summary for PR performance	Not identical to AUC; can vary with prevalence and interpolation	Comparing models in imbalanced datasets	Cross-site comparisons without considering prevalence differences
**DET Curve (Detection Error Trade-off)**	Plots False Negative Rate vs False Positive Rate (often log scale)	Highlights trade-offs at low error rates; useful for stringent diagnostic settings	Less familiar to medical audience; harder to interpret	High-sensitivity or high-specificity tasks; security-like detection tasks adapted to medical AI	General reporting in clinical papers unless clearly explained

**Calibration Curve / Reliability Diagram**	Compares predicted probability to observed event rate	Directly evaluates probability accuracy; clinically meaningful	Needs sufficient sample size; sensitive to binning strategy	Risk prediction models; decision support tools using probabilities	Pure classification tasks where only labels are used (but still helpful)
**Brier Score Curve / Calibration error visualization**	Summarizes probabilistic prediction error	Captures both calibration and discrimination	Harder to interpret clinically as a single number	Comparing probabilistic models; calibration evaluation	Interpreting without calibration plot or clinical threshold context
**Cumulative Gain Curve**	Shows fraction of true positives captured when targeting top-ranked predictions	Useful for prioritization and screening; intuitive for "top-N" evaluation	Depends on ranking quality; less standard in medical reporting	Triage systems, prioritizing high-risk cases	When clinical decision requires explicit probability threshold
**Lift Curve**	Shows improvement over random selection at different population fractions	Communicates operational benefit (workload reduction)	Can mislead if baseline prevalence differs; not universally familiar	Resource allocation; screening programs; radiology prioritization	Cross-site comparisons if prevalence differs significantly
**KS Curve / Kolmogorov–Smirnov plot**	Measures maximum separation between score distributions	Simple discrimination measure	Rarely used in clinical diagnostics; interpretation may confuse reviewers	Quick discrimination check	Should not replace ROC/PR reporting
**Decision Curve Analysis (DCA) Curve**	Net benefit across threshold probabilities	Directly links model to clinical decision utility	Requires clear assumptions about threshold probabilities and harm-benefit trade-offs	Clinical decision support; risk-based interventions	When clinical action thresholds are unknown or unrealistic
**Cost Curve / Expected cost plot**	Expected cost across thresholds based on cost ratios	Incorporates clinical cost weighting	Requires explicit cost assumptions; subjective	When FP/FN costs can be reasonably estimated	If cost assumptions are speculative or not justified
**Learning Curve**	Performance vs training sample size	Helps judge underfitting/overfitting and data sufficiency	Depends on dataset splitting strategy; can be noisy	Model development planning; dataset size justification	Direct clinical reporting of diagnostic accuracy

**Validation Curve**	Performance vs hyperparameter value	Helps model tuning and selection	Not clinically relevant; may be confusing in review papers	Algorithm development and optimization	Clinical translation sections unless simplified
**Threshold Performance Curve**	Metric value vs decision threshold	Helps choose clinically appropriate threshold	Requires clinical definition of acceptable trade-off	Selecting operating points for deployment	Reporting without linking to clinical consequences
**Section 4 — Bias, Fairness, and Robustness Assessment**					
**Metric / Approach**	**Definition**	**Pros**	**Cons**	**Appropriated Use**	**Unappropriated Use**
**Bias analysis (dataset bias identification)**	Whether training/test data are representative of the intended clinical population	Essential for trust; highlights sampling imbalance	Requires demographic metadata; bias may be hidden in unmeasured variables	Early dataset preparation; pre-deployment validation	If demographic data unavailable, conclusions about fairness may be incomplete
**Subgroup performance reporting**	Performance differences across groups (sex, age, ethnicity, scanner type, hospital site)	Clinically interpretable; directly addresses equity	Small subgroup sample sizes cause unstable estimates; multiple testing risk	Diagnostic AI deployment; regulatory reporting; multi-site studies	Avoid strong subgroup conclusions if confidence intervals are wide
**Stratified confusion matrices**	Error breakdown (TP/TN/FP/FN) within each subgroup	Transparent; supports safety analysis	Requires adequate subgroup size	Clinical safety review; fairness evaluation	Not feasible for many subgroups without aggregation strategy
**Difference in sensitivity/specificity across subgroups**	Whether miss rate or false alarm rate differs by subgroup	Clinically meaningful; relates to harm	May reflect prevalence differences rather than model bias	Screening/triage tools; safety-critical use	Must interpret with prevalence and spectrum effects
**Demographic parity / statistical parity**	Whether positive prediction rates are equal across groups	Simple fairness criterion	Often clinically inappropriate because disease prevalence differs by group	Administrative fairness auditing	Medical diagnostics where prevalence is biologically unequal
**Equalized odds**	Whether TPR and FPR are similar across groups	Harm-oriented fairness metric	Difficult to satisfy simultaneously with calibration; may reduce overall accuracy	High-stakes clinical decision support	Requires large samples and stable subgroup estimates

**Equal opportunity**	Whether TPR (sensitivity) is similar across groups	Focuses on missed disease equity	Ignores false-positive burden differences	Screening systems where missed cases are unacceptable	If false positives are also harmful (e.g., invasive follow-up)
**Predictive parity**	Whether PPV is equal across groups	Clinically interpretable for rule-in decisions	PPV depends on prevalence; equality may not be achievable	When positive results trigger costly interventions	Comparisons across groups with different prevalence without adjustment
**Calibration across subgroups**	Whether predicted probabilities match observed risk in each subgroup	Critical for risk-based decisions; supports safe probability interpretation	Requires enough subgroup data; may appear "good" with coarse binning	Risk prediction, prognosis models, clinical decision support	Avoid over-interpreting with limited subgroup size
**Disparate impact ratio**	Ratio of positive prediction rates between groups	Simple screening metric	Not clinically grounded; can flag expected prevalence differences	Initial fairness screening	Not sufficient for clinical fairness conclusions
**Fairness through awareness / clinical relevance review**	Whether protected attributes correlate with disease mechanism or confounders	Clinically realistic fairness framing	Requires domain expertise; not a numerical metric	Medical AI ethics discussions	Purely quantitative evaluation without clinical reasoning
**Robustness to noise / perturbation testing**	Whether predictions remain stable under noise, artifacts, missing data	Reflects real-world imaging and data quality	Must define realistic perturbations; may not generalize	Medical imaging, wearable data, EHR with missingness	Unrealistic perturbations can mislead robustness claims
**Sensitivity analysis (input variation)**	How output changes when input is slightly changed	Detects brittle behavior	Can be computationally expensive	Imaging AI; lab-value-based prediction models	Not meaningful if perturbations are not clinically plausible
**Out-of-distribution (OOD) detection / shift detection**	Whether model identifies cases unlike training distribution	Improves safety; supports human escalation	No universal standard; difficult evaluation	Multi-site deployment; new devices; new populations	OOD methods may fail silently; needs validation
**Stress testing across acquisition settings**	Performance across scanner types, protocols, institutions	Directly tests external validity	Requires multi-site datasets; costly	Radiology, pathology, ECG/EEG AI	Single-site studies cannot claim broad generalizability

**Adversarial robustness evaluation**	Model vulnerability to worst-case perturbations	Highlights security vulnerabilities	Some adversarial attacks are unrealistic clinically	Safety-critical environments; regulatory interest	Over-emphasizing adversarial tests can distract from real-world shift issues
**Uncertainty estimation (confidence/entropy-based)**	How confident the model is in its prediction	Helps triage uncertain cases to clinicians	Uncertainty may be miscalibrated	Clinical decision support; "human-in-the-loop" systems	Not sufficient alone to guarantee safe predictions
**Confidence interval reporting for subgroup metrics**	Uncertainty of fairness/robustness metrics	Prevents overclaiming; supports transparent reporting	Requires adequate subgroup sample sizes	Fairness reporting and deployment readiness	Avoid claiming bias without statistically meaningful evidence
**Bias mitigation strategies (reweighting, resampling, augmentation)**	Reducing imbalance and bias during training	Practical; can improve subgroup performance	May reduce overall accuracy; may create new bias	Pre-deployment model refinement	Must validate carefully to avoid unintended harm
**Threshold adjustment per subgroup**	Adjusting decision threshold to meet fairness targets	Improves sensitivity or PPV equity	Ethically and clinically controversial; complicates deployment	Some screening programs with clear goals	May violate clinical consistency and regulatory expectations
**Model auditing / governance framework**	Periodic review of performance and bias post-deployment	Aligns with clinical safety culture	Requires infrastructure and monitoring data	Real-world deployment, hospital AI governance	Not feasible in purely retrospective research papers without deployment plan
**Section 5 — Performance Metrics and Loss Functions**					
**Metric / Measure**	**Definition**	**Pros**	**Cons**	**Appropriated Use**	**Unappropriated Use**
**RMSE (Root Mean Square Error)**	Average magnitude of prediction error with larger errors penalized more	Sensitive to large errors; widely used; useful when large deviations are especially undesirable	Not robust to outliers; units depend on target scale; can be hard to interpret clinically without context	Regression-style clinical prediction (lab values, continuous risk scores, biomarker prediction)	When outliers dominate or when MAE is more clinically interpretable

**MAE (Mean Absolute Error)**	Average absolute difference between predicted and true values	Robust vs RMSE; directly interpretable in original units	Treats all errors equally (may under-penalize large clinical errors)	Continuous outcomes where "typical error" matters (e.g., BP prediction)	If large errors carry disproportionately high harm
**MSE (Mean Squared Error)**	Average squared error; strongly penalizes large errors	Smooth optimization objective; emphasizes large deviations	Very sensitive to outliers; not directly interpretable (squared units)	Model training and regression benchmarking	Human-facing reporting without also giving MAE/RMSE for interpretability
**Huber Loss**	Hybrid: behaves like MSE for small errors, MAE for large errors	Balances stability and robustness; less sensitive to outliers than MSE	Requires tuning the transition parameter; can be less intuitive	Training robust regression models in noisy clinical data	Reporting alone as an evaluation metric without clearer clinical error summaries
**Shannon entropy (prediction uncertainty concept)**	Uncertainty of predicted probability distribution	Interpretable as "model uncertainty"; useful for triage/escalation	High entropy ≠ wrong; depends on calibration and data distribution	Uncertainty-aware diagnostic support; flagging ambiguous cases	Using entropy as a "performance metric" by itself
**Cross-entropy loss**	Probability mismatch between true labels and predicted probabilities	Standard for probabilistic classifiers; supports stable training	Optimizing cross-entropy doesn't guarantee calibration or clinical utility	Training deep learning classifiers that output probabilities	Using it alone to claim clinical diagnostic performance
**KL divergence (Kullback–Leibler divergence)**	Difference between two probability distributions	Useful for distribution comparisons and information-theoretic framing	Not symmetric; can be unstable with zeros; interpretation needs care	Comparing predicted vs reference distributions; shift-type analyses	Presenting as a standalone "clinical performance" metric without explanation
**Predicted correlation (general)**	Association between predicted and true continuous outcomes	Simple summary of monotonic/linear association	High correlation can coexist with large absolute errors; not a full performance metric	Supplementary evaluation for continuous predictions	Replacing error metrics (MAE/RMSE) with correlation alone
**Pearson correlation**	Linear association strength	Familiar; useful when relationship is approximately linear	Sensitive to outliers; misses monotonic nonlinear patterns	Continuous outcomes with linear	Nonlinear monotonic relationships; heavy-tailed noise

				relationship assumptions	
**Spearman rank correlation**	Monotonic association using ranks	Robust to outliers; works with monotonic nonlinear relations	Less informative about absolute error; can tie heavily in discrete data	Ordinal or monotonic clinical relations; non-normal distributions	Using alone for clinical accuracy claims
**Kendall's tau**	Rank-based concordance between paired observations	Robust; interpretable with ties; good for ordinal biomedical measures	Can be lower magnitude than Spearman; less familiar to some readers	Ordinal ratings (severity grades), tied values, small-to-moderate samples	As the sole reported association statistic without explanation
**Balanced Accuracy**	Average of sensitivity and specificity	More reliable under class imbalance than accuracy	Still threshold-dependent; may hide PPV/NPV effects	Imbalanced diagnostic classification (rare disease screening)	Alone, without sensitivity/specificity and prevalence context
**Jaccard Index (classic / binary sets)**	Overlap between predicted and true sets (intersection/union)	Intuitive; widely used for set similarity (including label sets)	Sensitive to rare events; ignores clinical importance weights	Comparing presence/absence of conditions, findings, or label sets	When features/events have different clinical weights but treated equally
**Weighted Jaccard (discrete weighted elements)**	Jaccard similarity that incorporates weights/importance	Can reflect clinical relevance/rarity	Requires defensible weighting scheme; can be subjective	When some findings/diagnoses should count more than others	If weights are arbitrary or not clinically justified
**Multiset / addition-based Jaccard**	Similarity when elements can repeat (counts/frequency)	Captures intensity/frequency (e.g., repeated events)	More complex; interpretability decreases	Repeated clinical events (visits, admissions, symptom frequency)	When only presence/absence matters (simple binary labels)
**Continuous Jaccard (continuous sets/vectors)**	Jaccard-like similarity for continuous representations	Works for continuous biomedical features/embeddings	Less familiar; depends on representation and scaling	Comparing continuous clinical vectors, embeddings, modern ML representations	If readers need classic interpretability; requires careful explanation

**MCC (Matthews Correlation Coefficient)**	Confusion-matrix-based correlation-like score for binary classification	Strong under imbalance; uses all TP/TN/FP/FN; symmetric	Can be undefined in edge cases (degenerate confusion matrices); less intuitive	Binary diagnostic tasks with imbalance; robust single-number summary	Reporting alone without confusion-matrix rates; edge cases with zero rows/cols
**Hinge loss (binary)**	Margin-based loss used in SVM-like classifiers	Convex; supports large-margin separation	Not probabilistic; outputs not naturally calibrated	Training margin-based classifiers; optimization discussions	Clinical probability interpretation without calibration mapping
**Multiclass hinge loss (extension)**	Margin-based loss generalized to multi-class settings	Extends hinge loss beyond binary; useful in multiclass optimization	Same limitations as hinge loss: not probabilistic by default	Multiclass classification training/optimization	Using as a clinical performance metric rather than a training objective
**Cohen's kappa (inter-rater reliability)**	Agreement beyond chance between raters/labelers	Clinically meaningful for label quality; common in medical annotation	Influenced by prevalence and bias; can be misread as "accuracy"	Assessing reference standard reliability (multiple clinicians/annotators)	Using alone to claim model performance; always clarify it's an agreement measure
**Cohen's kappa (as classifier metric)**	Kappa computed from confusion matrix as a performance index	Offers chance-corrected agreement-style score	Shares kappa limitations; can disagree with MCC/accuracy	Supplemental classifier metric (with MCC/BA/PPV/NPV)	As headline metric without showing confusion matrix and prevalence context
**Section 6 — Cross-Validation**					
**Method / Approach**	**Definition**	**Pros**	**Cons**	**Appropriated Use**	**Unappropriated Use**
**Hold-out validation (train/test split)**	Performance on a single unseen subset	Simple; fast; easy to explain	High variance; results depend heavily on one split	Preliminary benchmarking; large datasets	Small datasets; high-stakes clinical claims
**k-fold cross-validation**	Average performance across k data partitions	Reduces variance vs single split; widely used	Can still leak information if data are correlated	General model evaluation during development	Patient-level correlation unless folds are grouped
**Stratified k-fold cross-validation**	k-fold CV preserving class proportions	Better for imbalanced clinical datasets	Still ignores patient clustering unless stratified carefully	Diagnostic classification with class imbalance	When multiple samples per patient exist

**Repeated k-fold cross-validation**	Stability of performance across multiple random splits	More reliable estimates; supports uncertainty assessment	Higher computational cost	Small-to-moderate datasets; reporting mean ± variability	Extremely large datasets with limited compute
**Leave-One-Out Cross-Validation (LOOCV)**	Performance when each sample is tested individually	Maximizes training data usage	High variance; computationally expensive; optimistic bias	Very small datasets; exploratory analysis	Clinical performance claims; correlated samples
**Monte Carlo cross-validation (random subsampling)**	Performance across many random splits	Flexible; easy to implement	Test sets may overlap; not fully exhaustive	Robustness checks; uncertainty estimation	Strict regulatory validation
**Nested cross-validation**	Unbiased model selection + performance estimation	Prevents optimistic bias from hyperparameter tuning	Computationally expensive; complex	Algorithm comparison; fair benchmarking	Routine reporting without hyperparameter tuning
**Grouped cross-validation**	Prevents leakage across related samples (e.g., patients)	Clinically correct; avoids overly optimistic results	Requires grouping metadata	Medical imaging; EHR with repeated measures	Ignoring grouping when samples are correlated
**Leave-One-Group-Out CV**	Generalization across groups (e.g., hospitals)	Directly tests external validity	Requires multiple groups; high variance	Multi-site clinical studies	Single-center datasets
**Time-series cross-validation**	Temporal generalization to future data	Respects chronology; realistic	Less data-efficient; harder to implement	Longitudinal EHR; monitoring data	IID assumptions; shuffled data
**Blocked time-based CV**	Performance across contiguous time blocks	Detects temporal drift	Sensitive to window size	Deployment readiness assessment	Small datasets with sparse time points
**Cross-validation with bootstrapping**	Stability and uncertainty of metrics	Distribution-free; flexible	Can violate independence assumptions	CI estimation for complex metrics	Clustered data without patient-aware resampling
**Patient-level cross-validation**	Ensures patient independence	Clinically essential	Reduces effective sample size	Imaging, waveform, longitudinal data	Sample-level CV in medical datasets
**Cross-validation for model comparison**	Statistical comparison of algorithms	Enables fair benchmarking	Requires correct statistical tests	Selecting between candidate models	Without correction for dependency

**Internal validation (general)**	Performance within development dataset	Necessary first step	Not evidence of generalizability	Model development	Clinical deployment claims
**Internal–external cross-validation**	Performance across subsets acting as external sites	Strong test of transportability	Requires multi-site data	Multi-center studies	Single-center datasets
**External validation (separate dataset)**	Performance on independent population	Gold standard for generalization	Data availability; costly	Pre-deployment clinical evaluation	Early development without sufficient data
**Prospective validation (linked to CV)**	Real-time performance on future cases	Highest clinical relevance	Logistically complex	Deployment readiness	Purely retrospective studies
**Cross-validation reporting transparency**	Reproducibility and trust	Improves interpretability	Often omitted	Any clinical AI study	Opaque validation pipelines
**CI reporting across CV folds**	Uncertainty of performance estimates	Prevents overclaiming	Requires correct aggregation	Clinical reporting	Single-point estimates only
**Section 7 — Computational Efficiency**					
**Metric / Measure**	**Definition**	**Pros**	**Cons**	**Appropriated Use**	**Unappropriated Use**
**Inference Latency**	Time required for a model to generate a prediction	Directly reflects real-time usability; clinically important for time-sensitive settings	Depends on hardware, batch size, optimization; may be misleading if not standardized	Emergency triage, ICU decision support, real-time radiology prioritization	Reporting without specifying hardware and environment
**Throughput**	Number of predictions processed per unit time	Reflects scalability for high-volume workflows	Can conflict with latency; depends on batch size	Large-scale screening programs; high-volume radiology pipelines	Comparing models without equal hardware or batch conditions
**Computational Complexity (Big-O)**	Theoretical growth of runtime with input size	Useful for theoretical comparison; indicates scaling behavior	Does not reflect real-world runtime; ignores hardware acceleration	Algorithm design and scalability discussion	Clinical deployment decisions without benchmarking
**Memory Usage (RAM/VRAM consumption)**	Amount of memory required during training/inference	Important for deployment	Highly platform-dependent; varies with implementation	Embedded devices, point-of-care systems,	Comparing without stating input size and model version

		feasibility on hospital hardware		limited-resource clinics	
**Model Size (number of parameters / storage size)**	Storage and transfer requirements	Helps estimate deployability and update speed	Does not always correlate with speed or accuracy	Mobile/edge deployment; hospital IT constraints	Assuming smaller always means faster or safer
**Training Time**	Time needed to train the model	Indicates development cost and feasibility of retraining	Depends on compute resources; not always clinically relevant	Iterative development; continual learning planning	Using it as a deployment metric (inference matters more clinically)
**Energy Consumption**	Energy used for training or inference	Relevant for sustainability and hospital infrastructure	Hard to measure consistently; depends on hardware	Large-scale model training; sustainability reporting	Small-scale studies where measurement is unreliable
**Power Consumption**	Instantaneous power draw during operation	Useful for edge devices and mobile clinical tools	Varies with hardware and load	Wearables, bedside devices, portable diagnostic systems	Pure cloud deployment comparisons
**Carbon Footprint / CO_2 emissions**	Environmental cost of training/inference	Supports responsible AI reporting	Estimates depend on energy source assumptions	Large models; institutional reporting; policy alignment	Over-emphasizing when clinical safety is primary focus
**Hardware Requirements (CPU/GPU/TPU constraints)**	Minimum compute platform needed	Directly affects adoption and feasibility	Often underreported; varies by optimization	Deployment planning in hospitals/clinics	Reporting without specifying exact system settings
**Scalability (horizontal/vertical scaling capacity)**	Ability to handle increased workload	Supports deployment in large networks	Requires infrastructure assumptions	National screening programs, multi-hospital networks	Single-center feasibility without realistic workload simulation
**Real-time feasibility assessment**	Whether model can meet workflow time constraints	Clinically interpretable; improves translation	Needs realistic workflow benchmarks	ICU, emergency imaging, surgical decision support	Purely retrospective studies without runtime measurement
**Cost-efficiency (compute cost per inference)**	Operational cost of running the model	Relevant for health system adoption	Depends on cloud provider and local IT cost	Health system budgeting and feasibility	Early research papers without deployment context

**Statistical efficiency (data efficiency)**	How much data needed to reach stable performance	Important for rare diseases and small datasets	Hard to generalize; depends on task complexity	Rare disease AI development	Not a substitute for external validation
**Algorithmic efficiency reporting**	Transparency about compute and runtime	Improves reproducibility and fairness of comparisons	Often missing in publications	Benchmarking studies and real-world deployment papers	Comparing models without equal compute budgets
**Section 8 — Statistical Tests of Significance**					
**Test / Approach**	**Definition**	**Pros**	**Cons**	**Appropriated Use**	**Unappropriated Use**
**p-value (general concept)**	Probability of observing results at least as extreme under the null hypothesis	Standard inferential tool; widely understood	Misinterpreted as effect size or clinical relevance; sensitive to sample size	Supporting evidence for comparisons	Should not replace effect sizes and confidence intervals
**Confidence intervals (CI)**	Range of plausible values for a metric or difference	Clinically interpretable uncertainty; supports reproducibility	Depends on assumptions and method	Reporting diagnostic metrics, subgroup analyses	Reporting only point estimates in clinical claims
**Paired t-test**	Whether mean difference between paired measurements is zero	Simple; powerful when assumptions hold	Requires approximate normality of differences; not robust to outliers	Comparing two models on the same subjects or folds	Non-normal differences or heavy-tailed distributions
**Independent t-test**	Whether two independent group means differ	Common; interpretable	Assumes normality and similar variances	Comparing continuous outcomes between groups	Skewed distributions or unequal variance without correction
**ANOVA (Analysis of Variance)**	Whether mean differs across ≥3 groups	Efficient multi-group comparison	Assumptions: normality, homoscedasticity; only indicates at least one difference	Comparing multiple algorithms or patient groups	Non-normal distributions without transformation or nonparametric alternative
**Welch ANOVA**	ANOVA variant robust to unequal variances	Better when variance differs	Still assumes approximate normality	Comparing groups with unequal variance	Extremely skewed distributions

**Post-hoc tests (Tukey, etc.)**	Identifies which groups differ after ANOVA	Controls family-wise error	Multiple comparisons inflate false positives if misused	Multiple model comparisons after ANOVA	Reporting without stating correction method
**Mann–Whitney U test**	Whether one distribution tends to have larger values than another	Nonparametric; no normality assumption	Tests distribution shift, not strictly medians; less power if normal	Comparing continuous metrics (e.g., errors) between two independent groups	Paired samples (use Wilcoxon instead)
**Wilcoxon signed-rank test**	Whether paired differences are symmetrically centered around zero	Nonparametric paired alternative	Assumes symmetric differences; less power than t-test if normal	Comparing paired model performance on same patients	Strongly asymmetric distributions or many ties
**Kruskal–Wallis test**	Whether ≥3 independent groups differ (rank-based)	Nonparametric ANOVA alternative	Does not specify which groups differ; requires post-hoc tests	Comparing multiple algorithms or groups when non-normal	Paired/repeated measures designs
**Chi-square test**	Association between categorical variables	Simple; widely used	Requires sufficient expected cell counts; ignores paired structure	Comparing class distributions; diagnostic category associations	Small samples or sparse contingency tables
**Fisher's exact test**	Exact test for association in small contingency tables	Works with small sample sizes	Computationally expensive for large tables; conservative	Small-sample diagnostic studies; rare events	Large datasets where chi-square is sufficient
**McNemar test**	Difference between paired binary outcomes	Ideal for comparing two classifiers on same cases	Requires paired design; limited to binary outcomes	Comparing two diagnostic classifiers on same dataset	Independent samples or multi-class without adaptation
**Mid-p McNemar**	Less conservative variant of McNemar	Better power in some settings	Less commonly reported; must justify	Small-sample paired classifier comparisons	When reviewers require strict exact test
**Permutation tests**	Significance by shuffling labels to create null distribution	Few distribution assumptions; flexible	Computational cost; needs correct exchangeability	Comparing model metrics when assumptions fail	Non-exchangeable data (time-series dependence)

**Bootstrap hypothesis testing**	Significance by resampling data	Works for complex metrics (AUC, F1)	Must respect clustering (patient-level); computational cost	Medical imaging, small datasets, complex metrics	Naive bootstrapping with correlated samples
**Bonferroni correction**	Adjusts alpha for multiple testing	Simple; conservative control of family-wise error	Overly conservative; increases false negatives	Few comparisons; safety-critical conclusions	Many comparisons where power is needed
**Holm–Bonferroni correction**	Stepwise improvement over Bonferroni	Less conservative than Bonferroni	Still can be strict	Multiple subgroup or model comparisons	Very high-dimensional testing
**False Discovery Rate (FDR) control (Benjamini–Hochberg)**	Controls expected proportion of false positives	Higher power than Bonferroni; standard in high-dimensional studies	Does not control family-wise error; must report q-values	Genomics, radiomics, large feature testing	Claims requiring strict control of any false positive
**Nadeau & Bengio corrected t-test**	Corrected significance test for repeated CV estimates	Accounts for dependency between folds	Still approximation; requires correct setup	Comparing two models under repeated CV	Using plain t-test on correlated folds
**Dietterich 5×2cv test**	CV-based paired test for model comparison	Designed for classifier comparison; reduces variance	Limited to two models; still debated	Benchmarking two classifiers	Multi-model comparisons without correction
**Effect size reporting (Cohen's d, mean difference)**	Magnitude of difference, not just significance	Clinically meaningful interpretation	Often omitted; depends on scale	Any clinical AI comparison	Reporting only p-values
**Clinical significance vs statistical significance**	Whether difference matters clinically	Aligns with real-world impact	Requires domain judgment	Diagnostic deployment and guideline compliance	Overclaiming based solely on small p-values

Section 9 — Clinical Requirements for Diagnostic AI Evaluation

Domain / Requirement	Definition	Pros	Cons	Appropriated Use	Unappropriated Use
**Lifecycle trust principles (WHO AI for health)**	Clinical AI must be evaluated beyond performance: safety, equity, accountability, oversight	Provides credible clinical framing; reviewer-approved	Too broad if not connected to measurable evaluation steps	As the conceptual foundation for evaluation and reporting	Avoid citing WHO alone as proof of effectiveness

**Intended use definition**	Clarifies population, setting, users, and decision point (screening, triage, second reading, confirmation)	Makes evaluation clinically meaningful; prevents misuse	Authors often state intended use vaguely; mismatch with dataset	Always required for clinical diagnostic AI studies	Avoid general claims of "diagnostic tool" without specifying workflow
**Workflow role classification (screening vs triage vs second read)**	Different workflows tolerate different error profiles	Prevents misleading conclusions; improves safety interpretation	Requires explicit discussion of consequences of FP/FN	Radiology triage, pathology decision support, screening tools	Do not assume one operating point fits all workflows
**Clinical error consequence framing**	FP/FN translate to overtreatment vs missed diagnosis	Converts metrics into patient safety relevance	Requires clinical reasoning; not purely statistical	High-stakes diagnostic AI deployment discussion	Avoid if not supported by intended use and context
**Clinical validity interpretation of metrics**	Confusion matrix, CIs, prevalence effects, and calibration must be explained clinically	Helps clinician/regulator understanding	Can become "narrative" if not tied to evidence	Clinical diagnostic papers and review frameworks	Avoid abstract interpretation without examples or thresholds
**Prevalence dependence of PPV/NPV**	Predictive values change across institutions even if sensitivity/specificity remain stable	Important for transportability; avoids false claims	Often ignored in single-site studies	Multicenter generalization and deployment planning	Avoid claiming PPV/NPV are stable across populations
**Calibration evidence requirement**	Probabilities must match real risk if used for decision-making	Essential for clinical risk stratification	Calibration is often poor even when AUC is high	Prognostic models, decision-support systems	Pure label-only tools may not require full probability calibration
**Ground truth / reference standard definition**	Defines what "correct diagnosis" means and how labels were obtained	Central to credibility; aligns with STARD-AI	Clinical truth is often uncertain; imperfect reference standards	Diagnostic accuracy research, imaging AI	Avoid "gold standard" language unless truly justified

**Reference standard uncertainty**	Labels may contain error from imperfect tests, follow-up gaps, or clinical disagreement	Prevents misleading model blame; improves fairness interpretation	Hard to quantify; can be ignored in retrospective datasets	Clinical diagnostic datasets, radiology labeling	Do not treat labels as error-free ground truth
**Inter-rater variation / agreement analysis**	Measures variability among clinicians/annotators	Adds transparency; supports reliability claims	Can be misused as performance metric	Radiology, pathology, ECG interpretation	Not sufficient to validate a model's clinical performance
**Label noise correlated with demographics**	Bias in diagnosis may differ by subgroup (e.g., underdiagnosis)	Addresses fairness in realistic clinical terms	Difficult to prove without deep data	Equity analysis; subgroup evaluation	Avoid strong causal claims without evidence
**Generalizability across sites**	Performance must hold across institutions, scanners, coding systems	Key clinical adoption requirement	Requires external data access; often missing	Multi-site imaging AI, EHR-based prediction	Single-site studies cannot claim broad clinical readiness
**Temporal validation**	Tests stability over time as practice and populations evolve	Detects drift risk; deployment-relevant	Often neglected; requires time-separated datasets	Real-world clinical implementation planning	Not possible if dataset lacks time metadata
**Distribution shift (dataset shift)**	Data patterns change across sites/time due to devices, protocols, coding	Explains why retrospective results may fail	Often discussed but not tested	Deployment risk analysis, monitoring planning	Avoid claiming robustness unless tested
**Grouped validation / leakage prevention**	Prevents training/testing on correlated samples (same patient, same hospital)	Prevents optimistic bias; clinically essential	Requires careful dataset design	Imaging, EHR, repeated measures	Sample-level CV without patient grouping is unsafe
**Stratified validation for fairness**	Ensures stable evaluation across subgroups	Improves equity assessment	Subgroup sizes may be too small	Bias auditing; demographic evaluation	Avoid overinterpreting subgroup differences without CIs
**Robustness testing under realistic conditions**	Evaluate performance under missing data, poor image quality, rare cases	Reflects real clinical environment	Perturbations must be clinically plausible	Medical imaging quality variation, EHR incompleteness	Unrealistic stress tests can mislead reviewers

**Clinical robustness as failure-mode discovery**	Robustness evaluation identifies boundaries where model fails	Strong safety framing; aligns with ISO risk logic	Requires careful reporting of limitations	Pre-deployment safety evaluation	Avoid claiming robustness if only internal validation performed
**Statistical significance testing for model comparison**	Ensures improvements are not due to chance, leakage, or overfitting	Evidence-based medicine compatible	p-values may exaggerate trivial differences	Comparing baseline vs proposed models	Avoid using significance alone without effect size
**Paired designs and nonparametric testing**	Same patients under different models require paired tests	Correct methodological practice	Often ignored in ML literature	Head-to-head model comparisons	Do not apply independent tests to paired outcomes
**Multiple comparison correction**	Controls false positives when testing many metrics/subgroups	Prevents inflated claims	Can reduce power; must justify method	Many metrics, thresholds, subgroup analyses	Avoid uncorrected "p-hacking" style reporting
**Prospective evaluation requirement**	Model should be tested on future cases in real workflow conditions	Strongest evidence of clinical readiness	Expensive and slow; requires clinical coordination	Deployment readiness evaluation	Not possible in early-stage retrospective-only studies
**Silent deployment / shadow mode testing**	Model runs in real environment without affecting care	Detects drift and calibration issues safely	Requires IT integration; ground truth may lag	Pre-deployment monitoring and risk assessment	Does not directly prove improved patient outcomes
**Interventional prospective evaluation**	AI output changes clinician decisions and outcomes	Measures real clinical utility	Complex; requires trial design	Clinical trials, workflow impact studies	Not feasible without governance and ethical approval
**Automation bias risk**	Clinicians may over-trust AI recommendations	Addresses real safety issue	Hard to measure retrospectively	Human factors evaluation, prospective trials	Avoid claiming "improves outcomes" without measuring behavior
**Alert fatigue / workflow overload**	Excess false alarms can burden staff	Clinically practical; deployment critical	Requires operational workflow metrics	Triage and screening tools	Not visible in offline evaluation alone
**Multicenter validation**	Independent cohorts across institutions	Strongest evidence of external validity	Data-sharing challenges	High-risk diagnostic AI	Single-site performance cannot replace multicenter evidence

**Site-stratified reporting**	Reporting per-site results, not pooled averages only	Reveals hidden failure modes	Increases reporting burden	Multicenter studies	Avoid hiding site failures with pooled metrics
**Post-deployment monitoring plan**	Evaluation continues after deployment (drift + performance tracking)	Addresses lifecycle safety	Requires infrastructure; labels may be delayed	Real-world deployment; regulatory compliance	Not meaningful if no monitoring mechanism exists
**Data drift monitoring**	Track input distribution changes over time	Early warning for failure	May not directly reflect performance	Continuous monitoring systems	Drift detection without revalidation may be incomplete
**Performance drift monitoring**	Track degradation of accuracy/calibration over time	Directly safety-relevant	Needs ground truth follow-up	Hospitals, registries, post-market surveillance	If outcome labels are unavailable or delayed
**Subgroup-aware surveillance**	Monitoring separately by demographic groups	Prevents hidden inequity	Requires protected attribute data	Fairness governance	Not feasible if demographic data cannot be collected ethically
**ISO/IEC governance standards (42001, 23894)**	Treat evaluation as ongoing risk management and accountability	Reviewer-friendly; structured governance	Standards are broad; must connect to evaluation actions	Institutional deployment and policy alignment	Avoid presenting ISO compliance as proof of clinical effectiveness
**Risk management framing (ISO 14971)**	FP/FN mapped to hazards, severity, likelihood, and mitigations	Strong clinical safety logic; very convincing	Requires structured risk analysis	Medical device-like AI, regulated tools	Overkill for purely exploratory research papers
**Quality management traceability (ISO 9001)**	Document processes, dataset creation, evaluation pipelines	Supports reproducibility and auditing	Adds documentation burden	Clinical translation and deployment	Not necessary for early exploratory prototypes
**Transparent reporting standards (STARD-AI, TRIPOD-AI)**	Require detailed reporting of dataset, reference standard, evaluation design	Strong credibility; reviewer-approved	Requires careful compliance; may reveal limitations	Diagnostic accuracy studies and prediction models	Avoid superficial "we followed STARD-AI" without actual compliance
**PROBAST / PROBAST-AI**	Risk of bias assessment tool for prediction models	Adds systematic bias evaluation	Requires expertise and structured reporting	Prediction modeling and clinical review papers	Not a replacement for validation

**CONSORT-AI / SPIRIT-AI**	Reporting standards for AI clinical trials	Strengthens prospective evidence credibility	Applies mainly to interventional studies	AI trials and prospective evaluation studies	Not applicable to purely retrospective validation papers
**Deployment readiness (technical feasibility)**	Includes latency, IT integration, stable input handling	Makes evaluation realistic	Often ignored in academic AI	Implementation planning	Not proven by offline performance metrics
**Human oversight definition**	AI is decision-support; clinicians must remain accountable	Aligns with WHO; reduces safety concerns	Must be operationally defined	High-risk diagnostic support	Avoid claiming "autonomous diagnosis"
**Cybersecurity and privacy governance (ISO/IEC 27001)**	Secure handling of data, models, access, updates	Increasingly important; reduces risk	Often treated superficially	Hospital deployment and clinical systems	Not a performance metric; should not be mixed with accuracy claims
**Supply chain security (SBOM, NIST SP 800-161)**	Tracks dependencies and software integrity	Reduces risk of compromise	Complex for academic settings	Deployment and regulated systems	Not essential for retrospective evaluation papers
**TRIAGE-aligned lifecycle evidence pipeline**	Intended use → validity → generalization → calibration → robustness/fairness → statistics → reporting → monitoring	Excellent narrative structure; reviewer-pleasing	Needs careful linking to earlier sections	Organizing the full paper and conclusion	Avoid overstating TRIAGE as a "validated standard" unless supported
